# **LipiDetective:** a deep learning model for the identification of molecular lipid species in tandem mass spectra

**DOI:** 10.1093/bib/bbag378

**Published:** 2026-07-27

**Authors:** Vivian Würf, Nikolai Köhler, Florian Molnar, Lisa Hahnefeld, Robert Gurke, Michael Witting, Josch K Pauling

**Affiliations:** LipiTUM, TUM School of Life Sciences, Technical University of Munich, Maximus-von-Imhof-Forum 3, Freising 85354, Germany; LipiTUM, TUM School of Life Sciences, Technical University of Munich, Maximus-von-Imhof-Forum 3, Freising 85354, Germany; LipiTUM, TUM School of Life Sciences, Technical University of Munich, Maximus-von-Imhof-Forum 3, Freising 85354, Germany; Goethe University Frankfurt, Institute of Clinical Pharmacology, Faculty of Medicine, Theodor Stern-Kai 7, Frankfurt am Main 60590, Germany; Fraunhofer Institute for Translational Medicine and Pharmacology (ITMP), and Fraunhofer Cluster of Excellence for Immune Mediated Diseases (CIMD), Sandhöfer Allee 1, Frankfurt am Main 60528, Germany; Goethe University Frankfurt, Institute of Clinical Pharmacology, Faculty of Medicine, Theodor Stern-Kai 7, Frankfurt am Main 60590, Germany; Fraunhofer Institute for Translational Medicine and Pharmacology (ITMP), and Fraunhofer Cluster of Excellence for Immune Mediated Diseases (CIMD), Sandhöfer Allee 1, Frankfurt am Main 60528, Germany; Metabolomics and Proteomics Core, Helmholtz Zentrum München, Ingolstädter Landstraße 1, Neuherberg 85764, Germany; Chair of Analytical Food Chemistry, TUM School of Life Sciences, Technical University of Munich, Maximus-von-Imhof-Forum 2, Freising 85354, Germany; LipiTUM, TUM School of Life Sciences, Technical University of Munich, Maximus-von-Imhof-Forum 3, Freising 85354, Germany; Institute for Clinical Chemistry and Laboratory Medicine, University Hospital and Faculty of Medicine Carl Gustav Carus of the Dresden University of Technology, Fetscherstraße 74, Dresden 01307, Germany

**Keywords:** lipidomics, lipid identification, mass spectrometry, deep learning, transformer neural network

## Abstract

Confidently identifying lipids in samples is a prerequisite for understanding their many functions in health and disease. However, accurate molecular lipid species identification via tandem mass spectrometry remains challenging. Most current approaches match measured spectra against an in-house reference library, which hinders the comparability of results. To address this challenge, the transformer model LipiDetective was developed and trained on a dataset of spectra from lipid standards, databases, and publications. Learning the characteristic lipid fragmentation patterns, LipiDetective can accurately annotate molecular lipid species in tandem mass spectra independently of the experimental setup. Integrated gradients reveals that LipiDetective focuses on peaks matching known fragments, making its predictions humanly interpretable. Therefore, LipiDetective offers a data-driven approach for molecular lipid species identification that may improve the comparability of annotations across different laboratories and analysis workflows.

## Introduction

Lipids have many important functions in biological systems; they serve as energy and heat sources, are involved in cell signaling, and act as the main structural elements of biological membranes [1]. To fulfill all these roles, they exhibit an enormous structural variety. As of July 2026, the lipid maps structure database (LMSD) [2] contained 28 481 unique curated lipid structures. Tandem mass spectrometry (MS2) can be employed to characterize lipidomes down to their molecular lipid species composition, meaning the acyl composition. In MS2, lipid molecules are fragmented, and the resulting ion fragments are recorded to produce a mass spectrum that is used to deduce structural information [3, 4].

Most approaches for identifying lipids in mass spectra currently rely on comparing the measured MS2 spectra to an in-house database of fragmentation spectra using a custom software pipeline [5]. Different software tools may use varying approaches for the preprocessing and identification steps, which, combined with the use of divergent spectral databases, leads to low reproducibility and comparability between laboratories. This issue is exacerbated by the many varying parameters in the experimental setup, such as collision energy, polarity mode, and type of mass spectrometer. In 2017, the lipid composition of the human plasma standard reference material 1950 was measured by 31 diverse laboratories, each employing their custom lipidomics workflow [6]. Across all laboratories, a total of 1527 unique lipids were identified on a sum composition level. However, only 339 lipids were reported by five or more laboratories.

This means that new and innovative approaches must be explored to improve comparability between results. Here, deep learning (DL) offers a promising alternative to spectrum matching approaches. DL has already been applied to a variety of tasks in mass spectrometry (MS) data processing, such as noise filtering, peak detection, and metabolite annotation [7]. However, recent machine learning approaches in mass spectrometry have primarily focused on small-molecule metabolomics and related structural elucidation tasks. For example, SIRIUS with CSI:FingerID [8] applies machine learning to predict molecular fingerprints for candidate ranking, CFM-ID [9] models fragmentation processes to facilitate the generation of predicted spectral reference libraries, and MSNovelist [10] employs an encoder–decoder network for *de novo* structure generation. While highly successful in small-molecule metabolomics, these approaches are not specifically tailored to lipidomics challenges such as standardized lipid shorthand reporting, combinatorial fatty acyl composition, and high molecular masses. The main argument for applying DL to the task of lipid identification from mass spectra is that a neural network will be able to learn and abstract the characteristic fragmentation pattern of each lipid species. Ideally, the neural network is then able to identify lipids independent of collision energy or other experimental setups and even generalize the learned lipid fragmentation patterns to unseen lipid species.

This motivated the development of LipiDetective, a transformer model that can identify lipids in MS2 spectra on a molecular lipid species level. Developing such a DL model comes with unique challenges, many of which center around the data on which the model is trained. Sufficient high-quality data must be available, meaning high-resolution spectra of confidently identified molecular lipid species. Unfortunately, this kind of data is still challenging to obtain. The dataset for training LipiDetective consists of spectra generated from measurements of chemical reference standards at varying collision energies specifically for this project, as well as raw data available in publications and databases. Even with this relatively small training dataset of 268 720 spectra, LipiDetective was able to learn lipid fragmentation patterns independently of the experimental setup. Model interpretability methods such as integrated gradients and visualization of attention weights show that LipiDetective learns to associate peaks with certain lipid substructures. This means that it does not have to rely on matching the overall spectra but can identify relevant lipid components from which it composes the final prediction. This allows it to even identify lipid species for which it has never previously encountered a spectrum.

LipiDetective provides a foundation for the community to build on and expand to further utilize the potential of DL for lipidomics. Rather than replacing established rule-based or library-driven identification pipelines, LipiDetective is intended to complement existing workflows. Spectral library matching is inherently limited to compounds present in the reference database and requires close correspondence between library and query spectra in terms of instrument type, collision energy, and other acquisition parameters. In contrast, LipiDetective learns fragmentation patterns from data rather than matching against individual reference spectra, which allows it to generalize more readily across experimental conditions. Rule-based approaches, although powerful, depend on instrument-specific fragmentation rules and curated knowledge that may overlook novel or unexpected fragment patterns. In contrast, LipiDetective learns informative fragment relationships directly from data without relying on predefined rules. Trained on spectra acquired across multiple instruments, collision energies, and data sources, LipiDetective aims to achieve robustness across experimental conditions to support cross-laboratory comparability. Because it operates directly on MS2 spectra, it reduces variability introduced by heterogeneous preprocessing pipelines.

Accordingly, LipiDetective could be useful as an additional validation layer within existing workflows to rank candidate annotations by prediction confidence and flag inconsistencies with rule- or library-based assignments. This makes it particularly valuable for large-scale lipidomics studies with incomplete libraries, varying experimental conditions, or the need for standardized molecular lipid species annotations. As annotations by a single software can often lead to a high rate of false positive identifications [11], a DL model that is able to identify molecular lipid species from fragment spectra in a fast, automated, and accurate manner can serve as an additional means of validation. Improving data quality and confidence in lipid identifications will enhance the comparability of results across different laboratories, thereby facilitating progress in the field of lipidomics.

## Materials and methods

### Datasets and data preparation

The training dataset comprises 268 731 MS2 spectra from six independent sources covering 2303 different molecular lipid species belonging to 31 different lipid classes. Additionally, 2002 MS2 spectra from blank measurements that do not show any target lipid species were added. This was done with the intention that the model would learn to predict nothing if no lipid species was present in a spectrum by directly returning the end-of-sequence token. Each source underwent dataset-specific preprocessing and quality filtering before being merged into a single Hierarchical Data Format 5 (HDF5) file [12] using h5py [13] (Table S1.1; detailed processing steps and retained spectra per source are described in Supplementary 1).

#### GNPS database

The database Global Natural Products Social (GNPS) Molecular Networking [14] was searched for publicly available datasets containing MS2 spectra of lipids. As of February 2026, there are three datasets available for download as JSON files that are focused on lipids.

The **PNNL** dataset is composed of 46 724 MS2 spectra from 1790 lipids measured by Thomas Metz’s group at the Pacific Northwest National Lab (PNNL) [15, 16]. The spectra were collected in positive and negative ionization modes using varying collision energy. The **HCE** dataset consists of 116 spectra measured in negative mode of lipids extracted from human corneal epithelium cells [17]. The **IOBA-NHC** dataset contains 197 MS2 spectra of lipids from human conjunctival cells (IOBA-NHC cell line) with 106 measured in negative mode and 91 in positive mode [18]. The HCE and IOBA-NHC libraries were generated by the Olivier Laprévote Lab from the Université de Paris.

All spectra were converted to Lipid Shorthand Nomenclature and filtered for basic spectral quality, see Supplementary 1. After preprocessing and removal of nonlipid or ambiguous entries, the three libraries were merged into a single GNPS dataset comprising 46 648 spectra (Table S1.2).

#### MITOMICS dataset

The mitochondrial orphan protein multi-omic CRISPR screen (MITOMICS) dataset was generated to establish a functional compendium of human mitochondrial proteins [19]. For this purpose, the proteome, metabolome, and lipidome of >200 CRISPR-mediated HAP1 cell knockout lines were profiled using MS. The raw files and corresponding mzML files are available in the MassIVE data repository under accession number MSV000086685. Across all cell lines, the publication identified 1349 lipids belonging to over 30 lipid classes using the LipiDex software [20].

The identifications were matched to the corresponding spectra in the 882 lipidomics mzML files using the provided retention time and precursor masses. Additionally, LMSD was queried to retrieve the exact mass of the identified lipid. As the adduct was not provided with the identification, it had to be manually determined as it is a component of the prediction output. For this purpose, a list of possible adducts was compiled from the LipiDex instructions, and using the exact mass as well as the precursor mass, the corresponding adduct was determined. While this approach cannot fully exclude residual ambiguity, it represents a pragmatic mass-based adduct inference strategy under a constrained set of biologically plausible adducts and was applied consistently across the dataset. A more detailed ambiguity analysis can be found in Supplementary 1.

#### Thermo

The Thermo dataset consists of measurements from a study published in 2023 on the effects of different storage conditions on lipid stability in mice tissue homogenates [21]. Samples from multiple tissues, including the liver, spleen, kidney, and heart, were measured using a Thermo Orbitrap Exploris 480 and normalized, stepped high-energy collisional dissociation (HCD) with 15, 30, and 50 eV. The MS2 spectra acquisition was data-dependent and with a mass resolution of 15 000. The lipids were identified on a sum species level utilizing the Compound Discoverer 3.1 from Thermo Fisher Scientific with the LipidBlast VS68 positive and negative libraries.

Building on this sum species identification in the study, the Alex$^{123}$ database [22] was used to match peaks in the MS2 spectra to known fragments to elucidate the fatty acid composition. At least three fragment peaks had to match to qualify as a possible identification. In the case where multiple isomeric lipid species were possible matches, the species whose matching peaks had the highest summed intensity was chosen.

Nevertheless, we acknowledge that this fragment-based upgrading from sum-level to molecular species annotation may introduce residual uncertainty, particularly for isomeric lipids with overlapping or nonspecific fragment ions. While the three-peak criterion reduces spurious assignments, it does not guarantee unique identification in all cases. The procedure was applied uniformly across the dataset, and spectra with insufficient fragment support were excluded to minimize erroneous annotations, see Supplementary 1 for more details.

#### Phospholipid standards

A reference dataset composed of single phospholipid standard measurements was generated for this study using shotgun MS2 with electrospray ionization (ESI) as an ionization technique. It contains MS2 spectra of 54 different phospholipid standards measured in positive and negative mode, except for phosphatidylglycerol (PGs), which were only measured in negative mode (Supplementary Table S1.8). Collision-induced dissociation (CID) was applied using stepped collision energies. The same standards were measured using mass spectrometers from three different instruments: Agilent 6560 IM QTOF-MS, Sciex X500R QTOF-MS, and Bruker maXis UHR-TOF-MS. Lipid standards were obtained from Avanti Polar Lipids and dissolved in an appropriate solvent. Stock solutions were diluted in methanol/isopropanol/chloroform (1/1/1, v/v/v) with 7.5 mM ammonium formate. Lipid were infused using a syringe-pump at a flow of 500 $\mu$l/h. MS2 spectra were collected in an multiple reaction monitoring-type acquisition with different collision energies. The mass-to-charge (m/z) values of different adducts covered were isolated and fragmented with collision energies from 10 to 50 eV in 2.5 eV steps. Data were exported as .mzML using MSConvert [23].

### Data preparation

#### Preprocessing

All profile spectra underwent smoothing, baseline correction, and peak picking, for which the pyOpenMS [24] library was used. Additionally, base peak normalization was performed for all spectra. This means that all peak intensities are divided by the intensity of the respective base peak, which is the peak with the highest intensity in the individual spectrum. Thus, the normalized intensities in every spectrum lie between zero and one. As the maximum precursor mass of the heaviest lipid in the dataset was $\sim$1520 m/z, all MS2 spectra were trimmed to an m/z range of 50–1600 m/z. The varying nomenclatures in the different data sources were manually adjusted to match the updated lipid shorthand notation [25].

Isomers tend to coelute in most methods leading to mixed spectra. Additionally, the ionization efficacy changes based on chain length and number of double bonds. The combination of these two effects means that the sn-position of the fatty acid side chains cannot be determined reliably solely based on an MS2 spectrum generated via CID. Therefore, fatty acids were always listed in order of increasing carbon chain length. In case a lipid contained multiple fatty acids with the same carbon chain length, they were sorted based on their number of double bonds in ascending order.

#### Noisy spectra filtering

An additional filtering step was performed for the phospholipid standard measurements to exclude particularly noisy spectra that either do not show any target lipid or have a very low signal-to-noise ratio (Supplementary Fig. S1.2). In general, the median intensity seems to remain similar between noisy and high-quality spectra, whereas the base peak tends to have a much lower intensity in the noisy spectra. This characteristic was used to estimate the noise level of a spectrum by dividing the median intensity by the base peak intensity. To generate the high-quality standards dataset, a threshold of 0.01 base peak normalized median intensity was chosen, and any spectra with a higher score were excluded (see Supplementary 1 for more details).

### LipiDetective architecture and training

**Figure 1:**
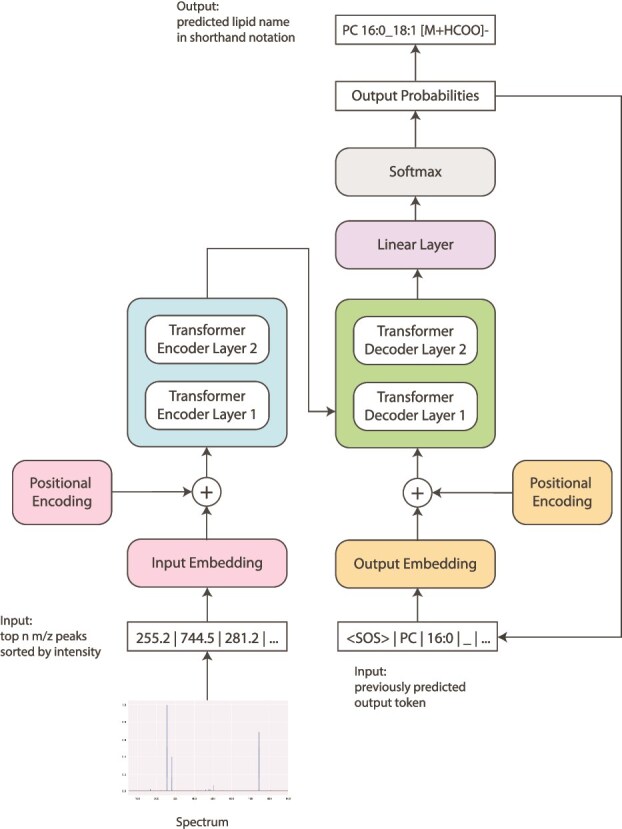
LipiDetective architecture based on the transformer model with an encoder–decoder structure.

LipiDetective is an adaptation of the transformer model [26] and approaches the problem of lipid identification as a sequence-to-sequence task. In this case, the input sequence is a list of the top n m/z values, ordered by decreasing intensity. The output sequence is the name of the molecular lipid species in shorthand notation, including the adduct present in the spectrum (Fig. 1). LipiDetective was implemented by modifying the transformer module provided by PyTorch [27]. To prepare the spectrum as input for the model, the m/z value of each input peak is truncated to a user-specified decimal point and then matched to its corresponding embedding vector. For the results presented here, n was set to 30, meaning that only the 30 most intense peaks of each spectrum are provided to the model. All MS$^{2}$ spectra are truncated to an m/z range of 50–1600, and accordingly, the maximum m/z value is fixed at 1600. After creating the input embedding, the positional encoding is then generated. Since the peaks have been ordered by decreasing intensity, the positional encoding incorporates how high peaks are in relation to each other without relying on their explicit values. After adding the positional encoding to each peak embedding, they are passed into the encoder layers of the transformer. The LipiDetective encoder consists of a standard two-layer PyTorch TransformerEncoder, each layer comprised of multi-head self-attention with four attention heads and a feedforward sublayer (dim_ffn=256) with source padding masks applied to ignore zero-padded peaks.

To prepare the output label during training, the name of each lipid species is parsed into a sequence of tokens using regular expressions that extract the lipid class, fatty acid chains, bond modifiers, functional groups, and adduct. Each token is drawn from a fixed vocabulary of 243 entries, and the sequence is framed by start-of-sequence and end-of-sequence markers. The token indices are mapped to learned embedding vectors and combined with sinusoidal positional encodings before being fed into the decoder. The LipiDetective decoder is a standard two-layer PyTorch TransformerDecoder that mirrors the encoder architecture. Each decoder layer applies padding masks to ignore both zero-padded peaks in the encoder output and padding tokens in the target sequence. During training, the decoder uses teacher forcing, receiving the ground-truth token sequence as input, and is optimized using cross-entropy loss computed over all token positions. A final linear layer projects the decoder output to the vocabulary size, yielding the predicted lipid species name and adduct for each input spectrum. At inference time, tokens are generated autoregressively using either greedy or beam search decoding.

Hyperparameter tuning was performed with Ray Tune [28] to find the optimal values for learning rate (0.004), batch size (512), dropout (0.1), embedding size (32), number of heads (4) and layers (2), size of the fully connected linear layer (256), number of input peaks (30), and decimal point cutoff (1 decimal place) with detailed results provided in Supplementary 3.

### Performance evaluation

#### Validation splits

The general approach for all validation splits was to select a set of lipid species, take all their corresponding spectra in the dataset, and exclusively assign them to the validation set. Assuring that the model has never seen any spectra of these species before prevents leakage, as technical or biological replicates of the same lipid species never appear in both sets, and allows for rigorous testing of the model’s generalization ability. Three random splits with different random seeds were created using the aforementioned approach to evaluate the influence of class imbalance on performance. An additional four splits were created in which all lipid species with a similar number of spectra (10, 100, 500, and 1000) were assigned to the validation set so that they would influence the validation accuracy roughly the same. Finally, separate splits were created for each lipid class by randomly selecting a number of species for a class to assign to the validation set. This was done for all lipid classes except ethanolaminephosphorylceramides (EPC) and sulfatides (SHexCer), as there were only two spectra each with these classes in the dataset. Comparing the performance on these 36 different splits should allow for a reasonable estimation of the model’s accuracy in general, as well as for over- and underrepresented lipid classes. Hyperparameter tuning was performed on the 100 spectra validation split as it has a higher lipid class diversity than the 500 and 1000 splits and contains more lipid species with common fatty acid side chain compositions than the 10 split. After determining the optimal hyperparameter settings (Supplementary Table S3.1), new instances of the model were trained from scratch for each of the different validation splits.

#### Accuracy metrics

Two accuracy measurements were implemented to evaluate LipiDetective’s performance. The first one represents total prediction accuracy and compares each predicted token with its corresponding counterpart in the label. Only if all tokens match will the prediction be classified as correct. However, this does not indicate how close the prediction was to the label. For this purpose, the lipid component accuracy was implemented, calculated by dividing the number of correctly predicted tokens by the total tokens. For example, the label PC 16:0_18:1 [M+HCOO]- consists of four components: the headgroup PC, the first side chain 16:0, the second side chain 18:1, and the adduct [M+HCOO]- (see Table 1). Suppose the model predicts PC 16:0_18:0 [M+HCOO]-, where three out of four components match, it would achieve a lipid component accuracy score of 3/4 = 75%. This provides another useful measure to evaluate how close the model’s predictions are on average to the label.

**Table 1:** Example prediction with three out of four components matching the label leading to a lipid component accuracy score of 75 %.

Label	PC	16:0	18:1	[M+CHOO]-
**Prediction**	PC	16:0	$\textcolor{orange}{18:0}$	[M+CHOO]-
**Match**	✓	✓	$\times$	✓

#### Interpretability

To understand the impact of individual features on LipiDetective’s predictions, the integrated gradients [29] method was employed as provided by the Captum [30] library. Furthermore, the spectrum embeddings and attention weights generated by the model during inference were extracted and analyzed. Dimensionality reduction using UMAP [31] was performed on the spectrum embeddings for visualization.

## Results and discussion

### Unbalanced lipid class distribution in training data poses a challenge for model evaluation

**Figure 2:**
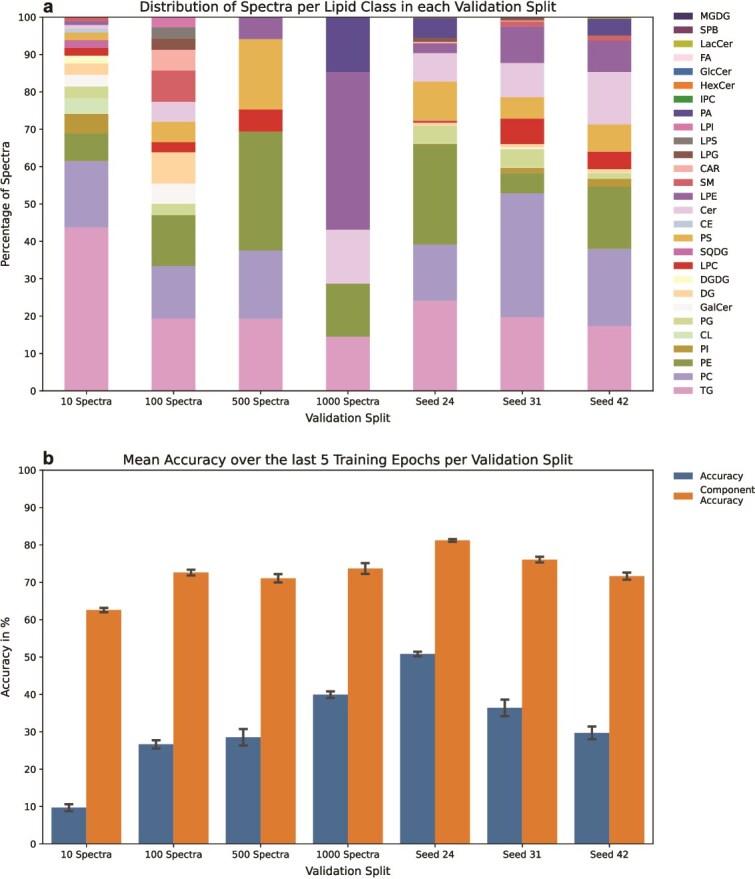
(a) Comparison of lipid class distribution in different validation splits. (b) Total prediction accuracy and lipid component accuracy of the tuned transformer model for each validation split. Bars represent the mean value computed over the final five training epochs, and error bars indicate the corresponding standard deviation.

The distribution of lipid classes in the training data (Supplementary Fig. S1.3A) is quite unbalanced, with over 50% of the spectra belonging to phosphatidylcholine (PC), phosphatidylethanolamine (PE), or triacylglycerol (TG) species. Additionally, the composition of lipid classes differs widely between the different sources (Supplementary Fig. S1.3B). Even though these data imbalance are expected, it poses a challenge when trying to accurately quantify the model’s performance. DL models generally require large amounts of data to perform well and tend to favor the majority class when using an imbalanced dataset for training. Therefore, the model is expected to perform much better on the highly prevalent lipid classes than on the ones represented by only a few spectra in the training data. This has to be taken into account when trying to evaluate the overall accuracy of its predictions. When splitting the dataset randomly, the model could achieve a high average accuracy if it predicts the abundant lipid classes correctly, even if it completely fails on the rare lipid species, as only a few spectra of the latter would be included in the validation data. For this reason, multiple splits were generated using different strategies (Fig. 2a).

The model achieves an average accuracy of $\sim$87% on the training data. However, its performance varies considerably across the validation splits. The random splits’ total prediction accuracy ranged from 29.7% to 50.8% (Fig. 2b). For spectra count splits, accuracy increased with the number of spectra, from 9.7% (10 spectra) to 39.9% (1000 spectra). This may be due to reduced complexity in validation splits with higher spectra numbers, which contain fewer lipid species and, consequently, fewer different classes and fatty acids (Supplementary Table S4.1). Additionally, lipid species represented with more spectra likely have common fatty acid compositions. Having seen a particular fatty acid more often makes it easier for the model to recognize. Lipid component accuracy is significantly higher than total prediction accuracy, averaging 60%–80%. This indicates that the model does not predict random lipids but is still able to identify, on average, more than half of the lipid component tokens correctly. This is confirmed by looking at the actual predictions for the validation dataset, which often differ by just one double bond, such as DG 18:1_18:2 [M+NH4]+ instead of DG 18:1_18:1 [M+NH4]+ (Supplementary Table S4.2).

**Figure 3:**
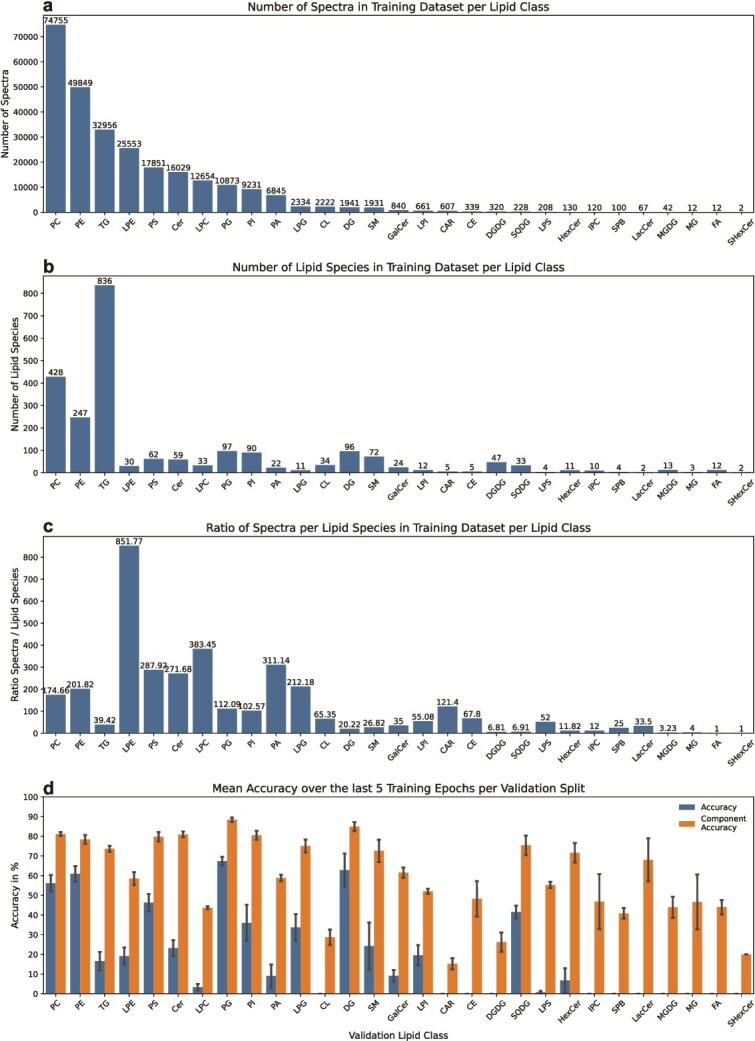
(a) Number of spectra per lipid class in the complete training dataset. (b) Number of molecular lipid species per lipid class in the training dataset. (c) Ratio of spectra/lipid species. (d) Mean validation accuracies per lipid class and corresponding standard deviations over the final five training epochs.

For the individual lipid class validation split, the model achieves the highest total prediction accuracy for PG, followed by PE, PC, and phosphatidylserine (PS) (Fig. 3). The strong performance on PG may be due to it only appearing in negative ion mode in the training dataset, thereby reducing a source of variation. In addition, the dataset contains a much smaller number of PG species (97) compared with PC (428) and PE (247). However, accuracy does not clearly correlate with spectra count per lipid species. Notably, the model performs exceptionally well on diacylglycerol (DG) despite its lower representation, likely transferring a pattern learned from a different lipid class onto the DG spectra. Mispredictions in the last training epoch often show TG with the same fatty acids as the correct DG label with one fatty acid being duplicated (Supplementary Table S4.3). Given their spectral similarity, the model may learn relevant features for DGs from the TG spectra. Increasing the number of DG spectra in the training data could help it learn to pay closer attention to the precursor peak to better distinguish between these classes.

Overall, the lipid class composition of the validation dataset significantly impacts model performance. Nevertheless, the results are promising, as LipiDetective can achieve high accuracies when identifying lipid species it has never seen before. Traditional spectrum matching approaches cannot identify unknown lipids that are absent from the reference database. Rule-based methods offer another promising approach to this challenge [32]. However, fragmentation rules vary between tools due to differing data sources and instrument dependencies, introducing inconsistencies that hinder comparability. Though modern rule-based systems are improving, they still rely heavily on established information about lipid fragmentation, potentially overlooking novel informative peaks.

### Encoder spectrum embeddings show that LipiDetective generalizes lipid fragmentation patterns across different experimental conditions

**Figure 4:**
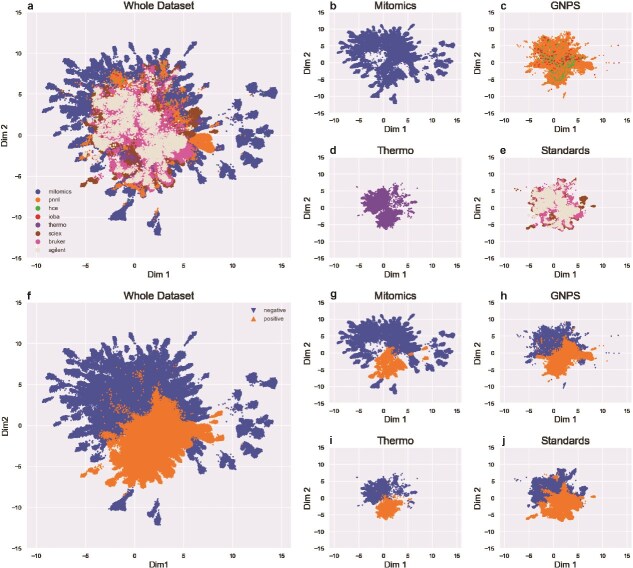
UMAP of spectrum embeddings of (a) the whole dataset and separated for each of the sources, (b) MITOMICS, (c) GNPS, (d) Thermo, and (e) the phospholipid standards colored by source. The same embeddings were also visualized colored by polarity for (f) the whole dataset and each of the sources (g) MITOMICS, (h) GNPS, (i) Thermo, and (j) the phospholipid standards.

One of the main goals of using a DL model is that the neural network learns to generalize lipid fragmentation patterns over different experimental conditions. This means the model could identify a lipid correctly, even if it has not previously seen a spectrum of this lipid with that exact collision energy or measured with that specific mass spectrometer. To further investigate the model’s abstraction abilities, the spectrum embeddings generated by the encoder were extracted for each lipid in the training dataset and visualized using UMAP (Fig. 4). Coloring the UMAP visualization by polarity shows that the model distinguishes between positive and negative measurement modes (Fig. 4f), as expected due to their distinct spectral peaks. However, embeddings from different sources overlap remarkably despite variations in lipid class composition (Fig. 4a). This is even more evident looking only at the phospholipid standard spectra (Fig. 4e), where the same lipid species were measured using mass spectrometers from three different manufacturers, Agilent 6560 IM QTOF-MS, Sciex X500R QTOF-MS, and Bruker maXis UHR-TOF-MS. While slight shifts exist, their embeddings largely overlap, suggesting that fragmentation pattern differences between instruments are not relevant enough for the model to differentiate between them.

This is also evident in the embeddings of single species, such as PC 16:0_18:1, the most prevalent lipid in the training dataset (Supplementary Fig. S4.1). Its embeddings form two main clusters corresponding to positive and negative modes, with spectra from different sources largely overlapping. A small separate cluster of Sciex spectra in negative mode appears closer to the positive mode spectra, likely due to differences in collision energies. Spectral comparisons suggest this outlier cluster represents spectra measured at low collision energy, where the precursor peak dominates, and fatty acid fragment peaks are minimal (Supplementary Fig. S4.2). This explains their proximity to positive mode spectra, which also lack fatty acid fragment peaks <400 m/z. Overall, the embeddings support the conclusion that LipiDetective achieves one of its primary goals: abstracting the lipid fragmentation patterns independently of the instrument or source. This means that the model does not need to see the spectrum of a lipid under the exact same experimental conditions to identify it correctly. This should allow for an increased comparability between measurements of the same sample by different laboratories.

Quantitative leave-one-source-out experiments on the Agilent, Sciex, and Bruker lipid standards datasets support this observation with LipiDetective achieving 78%–92% exact-match accuracy in zero-shot instrument transfer (Supplementary Fig. S4.9). Cross-instrument generalization is strongest for well-represented lipid classes, while accuracy depends on species coverage and dataset size of the held-out source.

### Integrated gradients show that Lipidetective considers well-known fragments in its prediction for the corresponding lipid component

**Figure 5:**
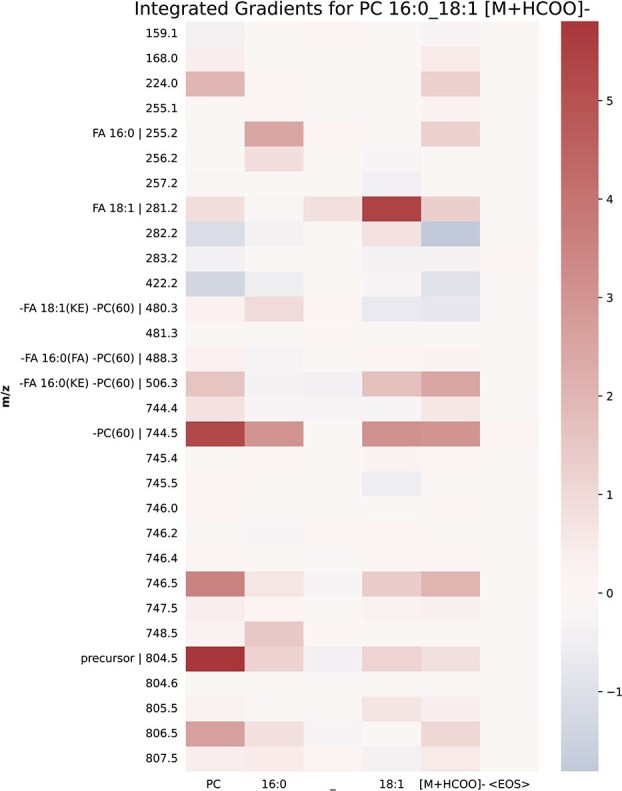
Integrated Gradients for a spectrum showing PC 16:0_18:1 [M+HCOO]-. Important peaks corresponding to known fragments in the Alex$^{123}$ database are annotated.

To provide more interpretability on the model’s prediction process, the integrated gradients algorithm was used to assign an importance score to each input feature with respect to each output token. Figure 5 shows these importance scores for the most frequently occurring lipid species in the training dataset, PC 16:0_18:1, measured in negative mode with the adduct [M+HCOO]-. The y-axis displays the input m/z values, which are annotated with expected fragments for this lipid species taken from Alex$^{123}$. The x-axis indicates the predicted output tokens.

Interestingly, the PC headgroup loss at 744.5 m/z as well as the precursor mass at 804.5 m/z achieve high importance scores for the prediction of the PC token. A human observer would also use these peaks as an initial indication of a PC in negative mode, supporting the hypothesis that the model evaluates the spectra in a manner similar to a human. Additionally, the model also considers isotopic peaks such as 746.5 m/z for the PC headgroup prediction. The peak at 255.2 m/z highly influences the prediction of 16:0 as the first fatty acid, which makes sense as the peak corresponds to the 16:0 fatty acid fragment. Similarly, the input peak at 281.2 m/z corresponding to the known fatty acid 18:1 fragment shows the highest importance score for the prediction of 18:1 as the second side chain. This indicates that LipiDetective has captured the pattern that certain peaks in a spectrum are characteristic of fragments that correspond to particular components of a lipid. There are some other peaks, such as 224.0 m/z, that are also relevant for the prediction but for which there is no annotation in the Alex$^{123}$ database. Understanding which molecule they might correspond to could reveal new information about the fragmentation processes of lipids and help create better reference spectra. Literature research reveals that this peak most likely corresponds to a fragment comprising the PC headgroup and glycerol backbone [33]. This shows that the model seems to primarily focus on human interpretable peaks that can be directly correlated to specific lipid fragments and corresponding isotopic peaks.

These importance scores could also be used to extract the most important peaks over all spectra the model trained on and create an *in silico* spectrum from them. For this purpose, the importance scores were calculated for each spectrum of a selected lipid species with a specific adduct. Then, they were summed over all output tokens and all spectra to create a single importance score for each input feature. The highest importance score was then set to one and used to scale the other scores accordingly. Finally, all input features that achieved <1 % of the maximum score were filtered out. Visualizing the remaining m/z values with the scaled importance scores as intensities results in a plot that looks incredibly similar to a mass spectrum with a medium-level collision energy (Supplementary Fig. S4.4). This is surprising as the model never sees the actual values of the peak intensities. The only indication it has of the intensities is by ordering the input vector with the m/z values by descending intensity. The *in silico* spectrum for PC 16:0_18:1 [M+HCOO]- generated using this method contains 24 m/z values, whereas most predicted spectra typically show fewer peaks. Additionally, it indicates the relevance of the peaks by providing the importance values as intensities.

Using integrated gradients showed unwanted biases in the dataset that affect the prediction. This needs to be considered when using LipiDetective to identify more uncommon lipids. To give the user a better indication of the confidence of the prediction, a function was implemented so that LipiDetective returns the top three predictions for a spectrum, including an overall probability score between zero and one for each prediction. For example, for the misprediction TG 16:0_22:6_22:6 [M+NH4]+, the model’s probability score reached only $\sim$0.4747, whereas it returns a score of 0.9970 for the correct prediction of the PC 16:0_18:1 [M+HCOO]- spectra in Fig. 5. This feature can serve as an additional quality check of the model’s prediction, allowing the user to determine their own confidence threshold.

### Visualization of attention weight matrices gives further insight into LipiDetective’s input processing

**Figure 6:**
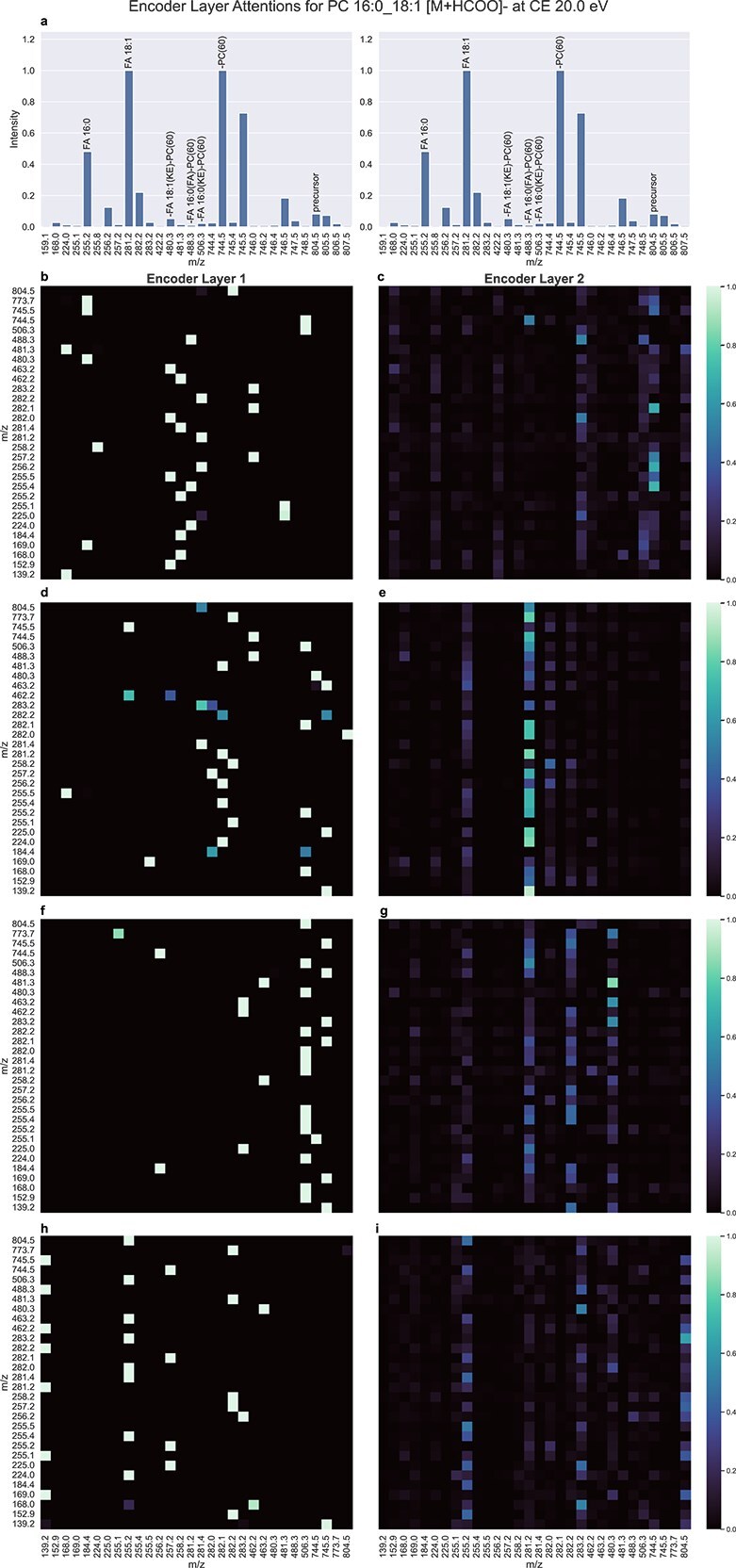
(a) Spectrum of PC 16:0_18:1 [M+HCOO]- at a collision energy of 20 eV. (b–i) Corresponding matrices of attention weights, with each row representing a different attention head and column 1 corresponding to the first and column 2 to the second encoder layer.

As the LipiDetective model is based on the transformer architecture, it utilizes attention, which can be used as an additional tool to improve its interpretability. High attention weights can hint at key inputs that provide contextual information for other peaks or even the whole spectrum. Figure 6 visualizes the attention for all four attention heads in both encoder layers for PC 16:0_18:1 measured in negative mode with adduct [M+HCOO]- at a collision energy of 20.0 eV. In the first encoder layer, most input peaks exhibit one high attention value focused on one other input peak. However, the attention weights in the second encoder layer are much more diffusely distributed across the input peaks. This could mean that the model focuses primarily on capturing local dependencies within the input data in the first encoder layer. Specific peaks exhibiting high attention to each other may indicate strong local correlations in the mass spectrum. Then, in the subsequent layer, the model integrates these local features to build a more global representation of the input, leading to a more diffuse attention pattern.

Making sense of the local dependencies between peaks is quite challenging. However, it is clear that each attention head of the second encoder layer seems to focus on a different aspect of the input spectrum. The first attention head focuses mainly on the headgroup, while the second concentrates on the fatty acid peaks. The third attention head also pays attention to the fatty acid peaks while also including the fragments representing the combined fatty acid and headgroup loss. The fourth attention head mainly focuses on the precursor mass. The peaks exhibiting high attention values in the second encoder layer show nearly none in the first encoder layer and vice versa. All this supports the theory that LipiDetective recognizes representative fragment peaks and then uses them in the second encoder layer to provide overall context for the other peaks in the mass spectrum.

## Conclusion

To our knowledge, LipiDetective represents the first transformer-based framework specifically designed to learn lipid fragmentation patterns for the identification of molecular lipid species in standardized lipid shorthand annotations from tandem mass spectra. Naturally, the novelty of this approach comes along with limitations. While the results above demonstrate the feasibility of transformer-based lipid identification from tandem mass spectra, several failure modes should be considered when applying LipiDetective in practice. Notably, many of these challenges, such as chimeric spectra, low signal-to-noise ratios, and instrument-dependent fragmentation, are not specific to LipiDetective but represent fundamental difficulties in computational lipid identification that affect rule-based and spectral library approaches alike.

LipiDetective’s data-driven approach is both its principal strength and its primary limitation. On one hand, it does not require manually curated fragmentation rules for each lipid class and adduct, and its compositional sequence generation theoretically enables generalization to novel lipid species by recombining known structural tokens, a capability that neither rule-based tools nor spectral library matching can provide. On the other hand, the model inherits the biases of its training data: it favors common fatty acids over rare ones, reflects the instrument types and adduct-class combinations present in training, and may propagate label uncertainty from spectra whose identifications could not be individually verified. In addition, given the relatively small training dataset, the model has likely not yet reached maximum performance. Expanding and diversifying the training set is therefore the most direct path to improving LipiDetective’s performance and is a priority for future versions. The quality of the training data should be enhanced by adding more lipid standard measurements, manually curated spectra and spectra of underrepresented lipid species and classes. As expanding the dataset is a time-intensive endeavor, LipiDetective already assists users by providing a confidence score between zero and one for each prediction, helping to assess reliability.

Nevertheless, interpretability analyses using integrated gradients showed that the model predominantly bases its predictions on well-established diagnostic fragments corresponding to lipid headgroups and fatty acid chains. This provides indirect validation that, despite potential label noise, the model learns chemically meaningful fragmentation patterns rather than spurious correlations. One planned improvement for the model is the integration of additional constraints during prediction. The beam search prediction process can be guided to ensure the predicted tokens generate a lipid species that always matches the precursor mass of the spectrum. This could be achieved by associating a mass equivalent with each token and maintaining a running sum of the total token mass for each beam throughout the prediction process. If the difference between the precursor mass and the calculated mass for a prediction surpasses a threshold value, the prediction would be discarded. This could eventually be combined with user-specified retention time ranges for certain lipid classes, so that only predictions with matching class tokens for the defined retention times are retained.

LipiDetective represents an opportunity for researchers to build upon as the pretrained model can easily be retrained on additional data, including specific lipid species or more uncommon lipid classes, to improve its performance for targets of interest. LipiDetective is also not limited to the lipid classes or adducts it has trained for. Further lipid class or adduct tokens can easily be added to the vocabulary file that LipiDetective uses. Updated models can then be saved and uploaded with the experimental data of a publication to provide reproducibility, allowing other laboratories to take advantage of such specialized models. Ideally, the training data would also be provided so that it will be possible to develop a model that was trained on a large corpus of spectra from many different laboratories using varying experimental setups. Another advantage of LipiDetective is its ability to perform predictions quickly. While the exact speed depends on the hardware, LipiDetective can typically annotate files containing around a hundred spectra in <5 s. With only $\sim$616 000 parameters, the saved models are highly compact, occupying $\sim$2.5 MB of memory. This low-latency prediction and minimal memory footprint make LipiDetective suitable for deployment directly on MS or attached computing devices. Real-time identification of compounds could be particularly beneficial in clinical diagnostics and other fields where immediate results are critical.

In conclusion, this study showcases the potential of DL models for molecular lipid identification directly from tandem mass spectra. At its current implementation, LipiDetective is intended to operate alongside, rather than in place of, established lipid identification pipelines. In practice, we envision it being used to assess the plausibility of molecular species assignments generated by spectral libraries or rule-based tools, particularly where identifications are ambiguous or reference spectra are unavailable. Discrepancies between LipiDetective predictions and conventional workflows may highlight spectra requiring manual inspection or additional validation, thereby improving overall robustness of lipid identification. While the current model’s accuracy is bounded by the size and diversity of its training data, LipiDetective is designed as an open foundation that improves as more reference spectra become available, and we invite the lipidomics community to contribute to expanding this resource. As training data grows to cover additional lipid classes, adduct types, and instrument platforms, data-driven approaches such as LipiDetective have the potential to enhance and complement established rule-based and spectral library tools in the lipidomics workflow to provide increased comparability between measurements by different laboratories.

**Key Points**

- LipiDetective is a transformer-based neural network trained to identify >2300 molecular lipid species from tandem mass spectra, representing the first application of deep learning to this challenge in lipidomics.
- The model learns lipid fragmentation patterns independently of the type of mass spectrometer, the collision energy, or other methodological variations, addressing reproducibility challenges between laboratories.
- LipiDetective displays early evidence of compositional generalization and has shown that it can correctly predict lipid species that it has never encountered during training, a capability unavailable to spectral library matching.
- The model focuses on biologically relevant peaks corresponding to known lipid fragments such as fatty acid chains or headgroups, similar to the way human experts analyze spectra, making its predictions interpretable.
- With only 616 000 parameters and the ability to annotate spectra in seconds, LipiDetective allows high-throughput analysis of large datasets.

## Supplementary Material

Supplementary-material_bbag378

## Data Availability

The LipiDetective source code can be accessed on GitHub via https://github.com/LipiTUM/lipidetective. All previously unpublished data will be made publicly available, with access details maintained in the referenced GitHub repository.
