# Supplementary material for: LipiDetective: a deep learning model for the identification of molecular lipid species in tandem mass spectra
Source: Brief Bioinform. 2026 Jul 27;27(4):bbag378. doi: 10.1093/bib/bbag378 (PMC13403187; doi:10.1093/bib/bbag378)
Supplement: Supplementary-material_bbag378 [file supplementary-material_bbag378.zip › LipiDetective_Supplement_1_bbag378.pdf]

Supplement 1: Training Dataset Composition and Processing

All spectra were base-peak normalized (intensity divided by maximum intensity), restricted to the 50-1600 m/z range, and required to contain at least 3 peaks. Lipid nomenclature was standardized to the Lipid Shorthand Nomenclature throughout, and fatty acid side chains were sorted by carbon count and degree of unsaturation for consistency.

Table S1.1: *Per-source breakdown of spectra counts, lipid species coverage, adduct types, and polarity distribution of the merged training dataset.*

Source	Spectra	Species	Exclusive Species	Adducts	Exclusive Adducts	Pos Spectra	Neg Spectra
agilent	9382	54	0	3	0	3882	5500
bruker	24412	54	0	3	0	12148	12264
sciex	46585	53	0	4	0	22940	23645
mitomics	113665	432	61	6	1	15085	98580
thermo	28039	936	522	4	0	12080	15959
hce	113	100	23	5	1	0	113
ioba	195	142	33	6	0	90	105
pnnl	46340	1631	1000	3	0	30198	16142
TOTAL	268731	2303	1639	9	2	96423	172308

S1.1 GNPS Spectral Libraries

Three sub-datasets from the GNPS (Global Natural Products Social Molecular Networking) platform were processed independently and merged. Because these are library spectra, the lipid identity was provided with each spectrum and no identification step was needed. The main processing effort was converting diverse naming conventions into the Lipid Shorthand Nomenclature.

The PNNL library, provided by the Pacific Northwest National Laboratory, contained 46,724 spectra (30,582 positive, 16,142 negative) from 1,790 lipid species annotated using the LIQUID tool. Non-lipid classes (CoQ variants, DGTSA; 376 spectra) and spectra with fewer than 3 peaks (8 spectra) were excluded, yielding 46,340 spectra. The IOBA-NHC library contained 197 spectra (91 positive, 106 negative) of lipids from human conjunctival cells. Two spectra of an unnamed compound were excluded, yielding 195 spectra. The HCE library contained 116 negative-mode spectra of lipids from human corneal epithelium cell lysate. Three spectra were excluded (an ambiguous wax ester entry and an unnamed compound), yielding 113 spectra. The three sub-datasets were concatenated without additional filtering into a combined GNPS dataset of 46,648 spectra (Table S1.2).

Table S1.2: *GNPS sub-datasets: spectra counts at each processing stage.*

Dataset	Raw spectra	Excluded	Final
PNNL	46,724	384	46,340
IOBA-NHC	197	2	195
HCE Cell Lysate	116	3	113
**GNPS Total**	**47,037**	**389**	**46,648**

S1.2 MITOMICS

Lipid identifications for the MITOMICS dataset were performed by the original authors using LipiDex, providing 1,189,135 identification entries with associated dot product scores and spectral purity values.

Processing occurred in two stages. First, the lipid nomenclature from LipiDex was converted to Lipid Shorthand Nomenclature. Lipid classes that did not fit into this system (specifically PE-NMe, PE-NMe2 (N-methylated phosphatidylethanolamines) and Methyl-PA) were removed (33,874 entries), leaving 1,155,261. The identifications were then filtered by spectral quality: a LipiDex DotProduct score $\geq$ 900 reduced the dataset to 643,316, and a spectral Purity threshold $\geq$ 90 further reduced it to 130,793 identifications. Adducts were determined by matching the observed precursor mass against the LIPID MAPS exact mass for each lipid plus each possible adduct form (tolerance ± 0.5 Da), removing entries without a matching adduct and yielding 105,817 identifications.

In the second stage, filtered identifications were matched to actual MS2 spectra in the raw mzML files by retention time (± 0.1 min) and precursor mass (± 0.5 Da). Ambiguous double matches were resolved by fragment matching. The final output contained 113,665 spectra-higher than the 105,817 identifications because multiple MS2 scans could match the same identification within the tolerance window (Table S1.3).

Table S1.3: *Mitomics dataset: spectra counts at each processing stage.*

Step	Identifications	Lost
Raw identifications	1,189,135	—
After nomenclature cleanup	1,155,261	33,874
After DotProduct $\geq$ 900	643,316	511,945
After Purity $\geq$ 90	130,793	512,523
After adduct matching	105,817	24,976
After mzML spectrum matching	**113,665**	—

S1.2.1 Quality filtering based on dot product and purity scores

Analysis of the dot product and purity score distributions across lipid classes revealed that purity was the dominant filter. Several major classes had a median purity of 0, indicating that co-isolated species overwhelmed the target precursor's fragments. Three lipid classes were completely eliminated: SM (33,243 $\rightarrow$ 0), SPB (1,041 $\rightarrow$ 0), and FAA (44 $\rightarrow$ 0). PC, the most abundant class (588,868 entries), retained only 4.4%. The low purity scores are most likely caused by co-elution and co-isolation in the complex biological matrix of 203 CRISPR-knockout cell lines measured by data-dependent acquisition. This class-level bias is an inherent trade-off: strict quality filtering ensures the training data contains only reliable fragmentation patterns, but at the cost of underrepresenting abundant classes prone to co-isolation. In this case we chose a stricter approach to avoid introducing systematic label noise.

S1.2.2 Adduct assignment

In the MITOMICS dataset, lipid molecular species identities with their corresponding precursor m/z values were already provided, but adduct types were not explicitly reported. During preprocessing of the mitomics dataset, adduct types were retro-assigned by comparing the observed precursor m/z against the theoretical precursor m/z computed from the LIPID MAPS exact mass plus each candidate adduct form, using a tolerance of $\pm$0.5 Da.

The retro-assignment therefore does not influence which lipid species is associated with a spectrum. Instead, the adduct is uniquely determined by the measured precursor m/z and the reported lipid species. Operationally, this step reconstructs missing metadata rather than performing an additional identification step and for every spectrum exactly one adduct satisfied the mass relationship, with no competing solutions occurring across the dataset.

We conclude that the adduct retro-assignment step does not introduce labeling ambiguity in the mitomics dataset. The principal source of potential label error for this dataset lies upstream, in the spectral library matching that provided the original molecular species identifications, which were accepted at face value after quality filtering (dot product $\geq$ 900, purity $\geq$ 90).

S1.2.3 Identification matching ambiguity

To evaluate potential label ambiguity introduced during spectrum-to-identification matching, we applied the matching procedure to all 882 mzML files (906,6440 MS2 scans) and recorded cases in which a single scan matched more than one lipid species. Matching used a precursor *m/z* tolerance of $\pm$ 0.5 Dalton, a retention-time tolerance of $\pm$ 0.1 minutes, polarity agreement, and a minimum of three fragment peaks. Among the 113,665 matched scans, only 377 scans (0.33%) produced multiple candidate identifications, arising from two recurring lipid pairs with nearly identical precursor masses (PC 16:0_16:0 vs. PE 18:1_22:4). Fragment-based disambiguation resolved 155 of 197 cases (79%), while the remaining unresolved cases were removed. Overall, 222 scans (0.2% of matched scans; 0.002% of all MS2 scans) were excluded due to unresolvable ambiguity. These results indicate that precursor-level matching was highly specific in this dataset and that ambiguous labels constitute a negligible fraction of the training data, making a meaningful impact on model training unlikely.

S1.3 Thermo

The Thermo dataset consists of targeted LC-MS/MS measurements acquired on a Thermo Exploris 480 instrument. Unlike the other datasets, identification was performed *de novo* during processing. The starting point was a target list of 675 lipid compounds with expected retention times and precursor m/z values in both polarities (635 positive, 434 negative). The raw data comprised 323 sample mzML files (162 positive, 161 negative; 4 blank files excluded) containing 2,569,307 MS2 spectra.

Target list lipid names were converted to the Alex123 database format to obtain expected fragment m/z values for each possible molecular lipid species. Of the 675 target compounds, 498 (73.8%) were found in Alex123; the remaining 177 were excluded. Identification proceeded by comparing each MS2 scan's retention time ($\pm$0.5 min) and precursor m/z ($\pm$1 Da) against the target list. Of the 2,569,307 raw MS2 spectra, 252,802 (9.8%) fell within the RT and m/z window of at least one target compound. Matched spectra were binned at 0.1 Da resolution and compared against expected fragment m/z values; a minimum of 3 matching fragments was required. When multiple molecular species matched, the one with the

most matching fragments was selected, with summed fragment intensity as a tiebreaker. The final dataset contained 28,039 identified spectra, corresponding to an identification rate of 11.1% of targetable MS2 spectra (Table S1.4).

Table S1.4: *Thermo dataset: spectra counts at each processing stage.*

Step	Count	Lost	Notes
Target list compounds	675	—	635 pos, 434 neg
Found in Alex123	498	177	483 pos, 323 neg
Raw MS2 spectra	2,569,307	—	323 files (4 blanks excluded)
MS2 in target window	252,802	2,316,505	$\pm$0.5 min RT, $\pm$1 Da m/z
Identified ($\geq$ 3 fragments)	**28,039**	224,763	11.1% of targetable

The low overall identification rate (1.1% of all MS2) reflects the data-dependent acquisition mode: most MS2 scans triggered on ions unrelated to the target list. Restricting to spectra within the RT and m/z window gives 252,802 candidates, of which 11.1% were positively identified by fragment matching. Unidentified spectra within the target window lacked sufficient fragment evidence, possibly due to low-quality fragmentation, co-isolation of non-target precursors, or isobaric non-lipid compounds.

S1.3.1 Fragment Matching Ambiguity

During preprocessing of the Thermo dataset, sum-level lipid annotations from the target compound list were upgraded to molecular species by matching observed MS2 fragments against in-silico predicted fragments from the Alex123 database. A minimum of three matching fragments was required, and when multiple molecular species candidates exceeded this threshold, the candidate with the most matching fragments was selected, with summed fragment intensity as a tiebreaker. This procedure raises the concern that the winning molecular species may not always be the correct one, particularly when multiple candidates match a similar number of fragments.

We evaluated this ambiguity in three tiers: a theoretical analysis of the candidate space per target compound, aan empirical analysis of the actual fragment matching on all 28,039 identified spectra, and an ablation study training on datasets with vs. without ambiguous Thermo spectra.

Tier 1: Theoretical candidate counts

Of the 498 target compounds found in Alex123 (483 positive mode, 323 negative mode), 35 were already annotated at the molecular species level and therefore had exactly one candidate. The remaining 771 sum-species entries (counted per mode) were expanded by Alex123 into all possible molecular species consistent with the sum formula.

Table S1.5: *Theoretical molecular species candidates per sum-species target compound from Alex123, by lipid class. Only classes with sum-species entries are shown.*

Lipid class	Sum-species entries	Mean candidates	Median	Max
TG	110	215.3	200.5	478
PE	88	52.2	47.0	147
PC	246	46.4	39.0	147
PI	40	38.4	36.5	67
PG	30	38.9	42.0	67
PS	40	35.0	30.5	67
DG	30	33.8	32.5	67
PA	3	25.3	23.0	32
SM	80	19.0	22.0	33
SHexCer	12	10.1	11.0	11
LPC, LPE, LPG, LPI, LPS	92	1.0	1.0	1

The candidate space varies dramatically by lipid class (Table S1.5). Triacylglycerols (TGs) have by far the most candidates, with a mean of 215 molecular species per sum formula and a maximum of 478 (TG 58:6). Diacyl phospholipids (PC, PE, PI, PS, PG) had 35-52 candidates on average. Lyso-lipids were unambiguous by definition, as a single fatty acid chain uniquely determines the molecular species from the sum formula.

Tier 2: Empirical ambiguity on actual spectra

We processed all 327 mzML files (164 positive, 163 negative; blank files excluded) using the matching criteria of retention time $\pm$0.5 min and precursor m/z $\pm$1 Da, and recorded *all* molecular species candidates passing the three-fragment threshold for each spectrum, not just the winner.

Of the 28,039 identified spectra, 12,397 (44.2%) had an unambiguous assignment where exactly one molecular species passed the fragment threshold. The remaining 15,642 spectra (55.8%) were ambiguous, with two or more candidates passing (Table S1.6).

Table S1.6: *Empirical fragment matching ambiguity across all 28,039 Thermo spectra.*

Category	Spectra
Total identified spectra	28,039
Unambiguous (1 candidate)	12,397 (44.2%)
Ambiguous ($\geq$ 2 candidates)	15,642 (55.8%)
Fragment margin distribution (ambiguous spectra only):	
Margin 0 (tied; intensity tiebreaker)	5,501 (35.2%)
Margin 1 fragment	5,590 (35.7%)
Margin 2 fragments	2,306 (14.7%)
Margin $\geq$ 3 fragments	2,245 (14.4%)
By target annotation level:	
Sum-species targets	27,558 spectra, 15,642 ambiguous (56.8%)
Molecular-species targets	481 spectra, 0 ambiguous (0.0%)

Among the ambiguous spectra, 35.2% (5,501) had a fragment margin of zero-meaning the winning and runner-up candidates matched the same number of fragments, and the winner was determined solely by summed fragment intensity. A further 35.7% (5,590) had a margin of one fragment. Only 29.1% of ambiguous spectra had a margin of two or more fragments.

The ambiguity was strongly class-dependent (Table S1.7). TGs were the most affected, with 84.9% of spectra ambiguous and a mean of 21.4 passing candidates per spectrum. This is expected: TG molecular species that share the same sum formula differ only in the distribution of carbon atoms and double bonds across three acyl chains, and these structural isomers produce extensively overlapping fragment ion patterns. The mean fragment margin for TG was only 1.0, indicating that the winning candidate typically led by at most one fragment.

Table S1.7: *Empirical fragment matching ambiguity by lipid class. Classes are ordered by total spectra.*

Lipid class	Spectra	Ambiguous	% ambiguous	Mean candidates	Mean margin
TG	10,669	9,062	84.9	21.4	1.0
PC	7,717	2,223	28.8	1.3	2.7
PI	2,333	1,484	63.6	22.1	3.4
PE	2,463	1,396	56.7	2.4	2.6
PS	2,362	1,357	57.5	2.0	3.2
LPE	861	0	0.0	1.0	3.7
PG	623	90	14.4	1.2	3.0
Cer	358	0	0.0	1.0	3.8
DG	259	29	11.2	1.1	2.9
LPI	146	0	0.0	1.0	4.1
HexCer	123	0	0.0	1.0	3.5
LPS	64	0	0.0	1.0	3.0
SM	33	1	3.0	1.0	2.9
LPG	26	0	0.0	1.0	3.5
SHexCer	2	0	0.0	1.0	3.0

Phosphatidylinositols (PI), predominantly analyzed in negative-ion mode, showed high ambiguity (63.6%) despite having fewer theoretical candidates than TGs. In this mode, spectra are dominated by inositol-phosphate headgroup fragments, concentrating signal in class-level ions while providing comparatively limited information on fatty-acyl composition. PE and PS exhibited moderate ambiguity (56.7–57.5%), consistent with their characteristic but less class-exclusive neutral-loss pathways in positive mode (e.g., loss of 141 Da for PE and 185 Da for PS). In contrast, PC, analyzed in positive-ion mode, showed substantially lower ambiguity (28.8%). The diagnostic phosphocholine fragment at m/z 184.07 strongly constrains class identification, effectively narrowing the hypothesis space early in decoding, while accompanying neutral losses retain information about fatty-acyl composition.

These results indicate that the sum-to-molecular species upgrade introduces substantial labeling uncertainty for TGs and moderate uncertainty for diacyl phospholipids. The Thermo dataset contributes 28,039 spectra (10.4%) to the merged training set of 268,731. Within the Thermo subset, the 9,062 ambiguous TG spectra represent the highest-risk labels. However, TGs comprise a minority of the overall training data, and the fragment-based selection still constrains the label to a biologically plausible molecular species even when ambiguous. We note that this analysis quantifies the *potential* for mislabeling; the actual error rate may be lower, as the winning candidate may nonetheless be correct in many ambiguous cases.

Tier 3: Training with vs. without ambiguous spectra

Thermo label ambiguity has minimal impact on model performance. Of the 28,039 Thermo spectra in the training set, 15,642 (55.8%) had multiple candidate species passing the 3-fragment threshold during sum-to-molecular upgrade. To assess whether this ambiguity introduces harmful label noise, we trained four model variants with progressively filtered training data: the full baseline (270,733 spectra), removal of all ambiguous Thermo spectra (−15,642), removal of only low-confidence Thermo spectra where the fragment margin between winner and runner-up was ≤1 (−11,091), and removal of all Thermo spectra (−28,039). All variants used identical hyperparameters and seed. We evaluated each on the independently-annotated MassBank test set, restricting to the 1,057 spectra whose species+adduct combination was present in all four training sets to ensure a fair comparison. Removing ambiguous Thermo spectra marginally improved exact match accuracy from 24.7% to 27.0% and average custom accuracy from 52.7% to 55.3%. Targeted removal of only low-confidence cases (fragment margin ≤1) yielded the highest average custom accuracy (56.8%). Removing all Thermo spectra yielded intermediate performance (25.9% exact, 54.9% custom), confirming that unambiguous Thermo data remains beneficial while ambiguous labels are mildly detrimental. Overall, the differences across variants are modest (∼2 pp exact match, ∼4 pp custom accuracy), indicating the model is robust to the annotation uncertainty inherent in fragment-based species assignment.

S1.4 Phospholipid Standards

The phospholipid standards dataset consists of MS2 spectra from 54 individually purchased phospholipid standards (PC, PE, PA, PG, PS classes), each measured on three mass spectrometers (Agilent, Bruker, Sciex) in both positive and negative ionization mode with multiple adducts. Each lipid/vendor/mode/adduct combination was stored as a separate mzML file containing replicate MS2 scans acquired across a collision energy ramp. In total, 294 mzML files contained 88,575 raw MS2 spectra (Agilent: 9,894; Bruker: 27,888; Sciex: 50,793).

Profile-mode spectra (Agilent, Bruker) were preprocessed using pyOpenMS: smoothing (Savitzky-Golay filter), baseline correction (TopHat morphological filter), and peak picking (PeakPickerHiRes). Sciex spectra were already centroided and skipped these steps. Individual peaks with near-zero intensity (< 0.005 after normalization) or outside the 50-1600 m/z range were removed. This processing was nearly lossless: only 204 of 88,575 spectra (0.2%) were discarded because all peaks fell below the intensity threshold or outside the m/z range.

Quality filter threshold

The most impactful filtering step was quality-based filtering. Each spectrum received a quality score defined as the median of its normalized peak intensities. A low median (< 0.01) indicates high dynamic range with a few dominant fragment peaks and many small ones-the expected pattern for a genuine fragmentation spectrum. A high median indicates noise-dominated spectra as can be seen in Figure S1.2. Of the 88,371 processed spectra, 80,379 (90.9%) fell in the high-quality category and were retained. The remaining 7,992 spectra were discarded. Overall, 90.7% of raw MS2 spectra were retained (Table S1.9).

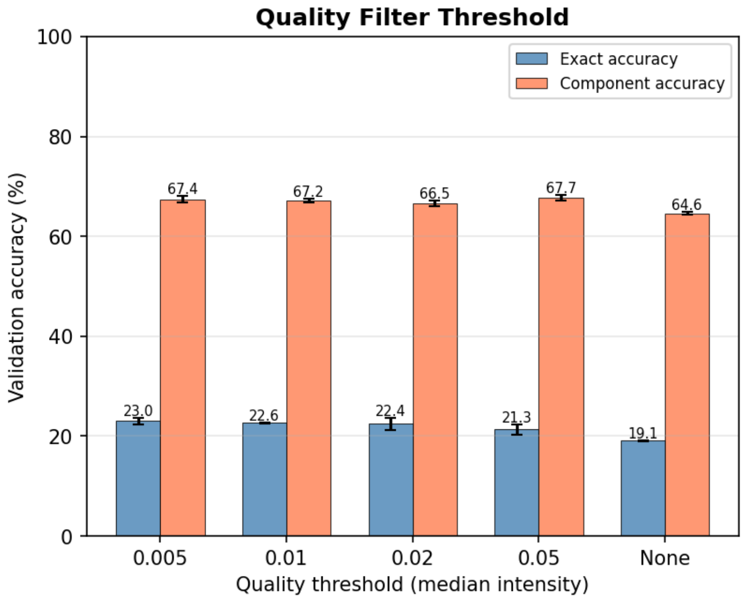

Figure S1.1: *Effect of the quality filter threshold on validation accuracy on the 100-spectra validation set. The quality filter removes spectra whose base-peak-normalized median intensity exceeds the given threshold, targeting low-information spectra. Exact accuracy (blue) measures full lipid nomenclature matches, while component accuracy (orange) measures the mean accuracy across individual lipid components (headgroup, sidechain length, double bonds, etc.). Values represent the mean ± standard deviation of the last three training epochs.*

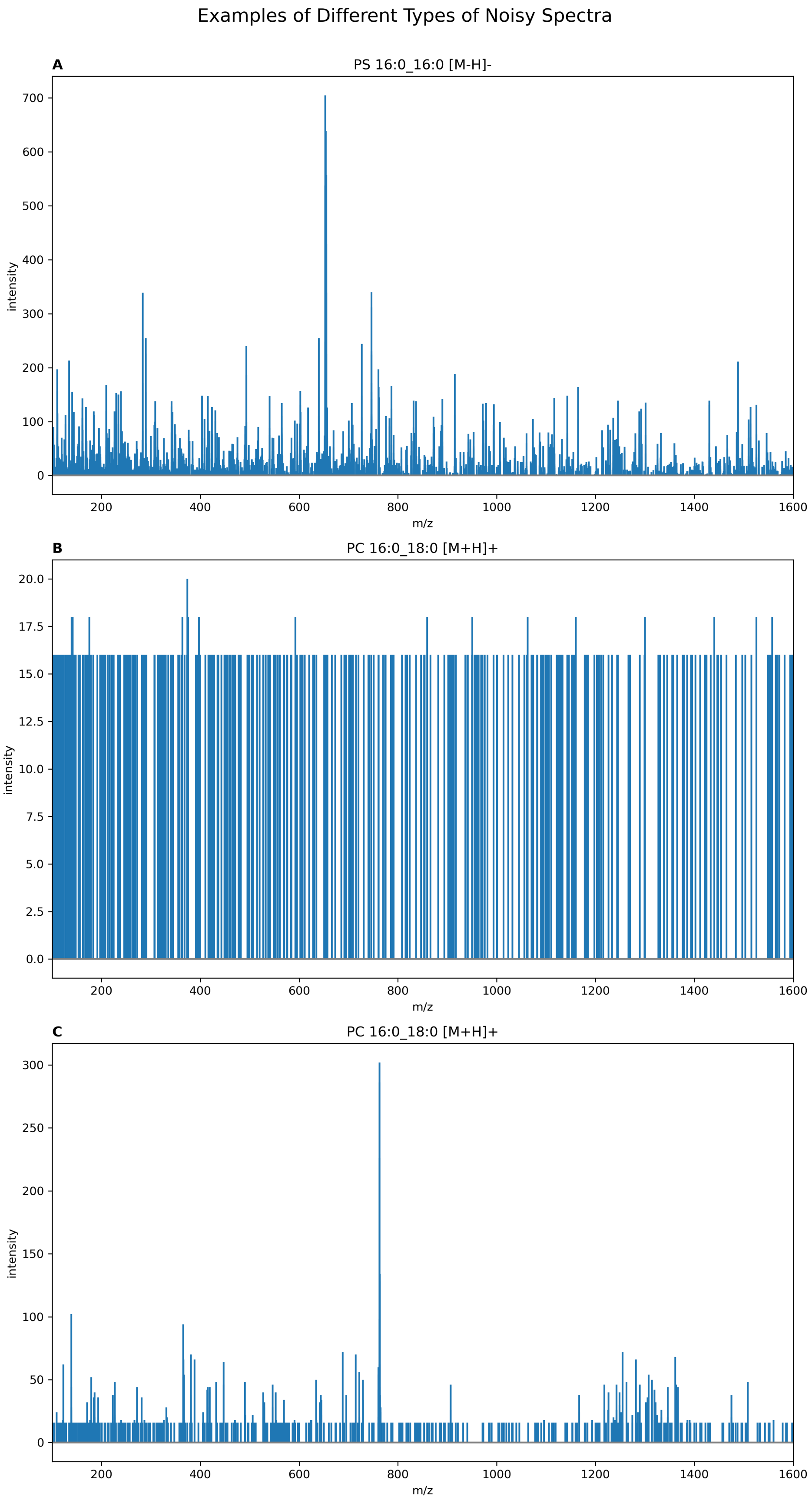

Figure S1.2: *Examples of noisy spectra filtered from the training dataset using the base peak normalized median intensity. A) MS2 spectrum not containing the target lipid due to an accidentally selected precursor mass of 743.5 Da instead of the true precursor mass of 734.5 Da for lipid standard PS 16:0_16:0 [M-H]- (0.022 normalized median intensity). B) MS2 spectrum of standard PC 16:0_18:1 with adduct [M+H]+ and selected precursor mass of 760.6 Da that seems to show only noise (0.800 normalized median intensity). C) A spectrum from the same file as B) with a very low signal-to-noise ratio showing some target compound fragment peaks (0.020 normalized median intensity).*

To find the ideal spectrum quality filter threshold we analyzed the performance of the model on the 100-spectra validation split using a thresholds ranging from 0.005 to 0.05, and also with no threshold applied. From the full dataset of 278,725 spectra, thresholds of 0.005, 0.01, 0.02, and 0.05 retained 265,649 (4.7% removed), 270,733 (2.9%), 272,946 (2.1%), and 274,105 (1.7%), respectively. The strictest threshold (0.005) achieved the highest exact accuracy (23.0%, component: 67.4%), but only marginally outperformed the 0.01 threshold (22.6%, component: 67.2%) (Figure S1.1) while discarding nearly twice as many spectra. Without any quality filtering, both exact and component accuracy dropped noticeably (19.1%, component: 64.6%), confirming that removing low-information spectra is beneficial. We selected a threshold of 0.01 as a practical compromise between filtering efficacy and data retention. Noise filtering was only applied to the phospholipid standard dataset, where spectra were intentionally acquired across a wide collision energy range and therefore included a substantial fraction of low-information spectra. No spectra were discarded from the other datasets beyond the here specified preprocessing.

Table S1.8: *Lipid species that were measured to create the phospholipid standards data source.*

Nr	Lipid Species	Positive Adducts	Negative Adducts
1	PA 14:0_14:0	[M+Na]+	[M-H]-
2	PA 16:0_16:0	[M+Na]+	[M-H]-
3	PA 16:0_18:1	[M+Na]+	[M-H]-
4	PA 16:0_18:2	[M+Na]+	[M-H]-
5	PA 16:0_20:4	[M+Na]+	[M-H]-
6	PA 16:0_22:6	[M+Na]+	[M-H]-
7	PA 17:0_17:0	[M+Na]+	[M-H]-
8	PA 18:0_18:0	[M+Na]+	[M-H]-
9	PC 14:0_14:0	[M+H]+	[M+HCOO]-
10	PC 15:0_15:0	[M+H]+	[M+HCOO]-
11	PC 16:0_16:0	[M+H]+	[M+HCOO]-
12	PC 16:0_18:0	[M+H]+	[M+HCOO]-
13	PC 16:0_18:1	[M+H]+	[M+HCOO]-
14	PC 16:0_18:2	[M+H]+	[M+HCOO]-
15	PC 16:0_20:4	[M+H]+	[M+HCOO]-
16	PC 16:0_22:6	[M+H]+	[M+HCOO]-
17	PC 17:0_17:0	[M+H]+	[M+HCOO]-
18	PC 18:0_18:0	[M+H]+	[M+HCOO]-
19	PC 18:0_18:1	[M+H]+	[M+HCOO]-
20	PC 18:0_18:2	[M+H]+	[M+HCOO]-
21	PC 18:0_20:4	[M+H]+	[M+HCOO]-
22	PC 18:0_22:6	[M+H]+	[M+HCOO]-
23	PC 20:0_20:0	[M+H]+	[M+HCOO]-
24	PC 22:0_22:0	[M+H]+	[M+HCOO]-
25	PE 14:0_14:0	[M+H]+	[M-H]-
26	PE 15:0_15:0	[M+H]+	[M-H]-
27	PE 16:0_16:0	[M+H]+	[M-H]-
28	PE 16:0_18:1	[M+H]+	[M-H]-
29	PE 16:0_18:2	[M+H]+	[M-H]-
30	PE 16:0_20:4	[M+H]+	[M-H]-
31	PE 16:0_22:6	[M+H]+	[M-H]-
32	PE 17:0_17:0	[M+H]+	[M-H]-
33	PE 18:0_18:0	[M+H]+	[M-H]-
34	PE 18:0_18:1	[M+H]+	[M-H]-
35	PE 18:0_18:2	[M+H]+	[M-H]-
36	PE 18:0_20:4	[M+H]+	[M-H]-
37	PE 18:0_22:6	[M+H]+	[M-H]-
38	PG 14:0_14:0		[M-H]-
39	PG 15:0_15:0		[M-H]-
40	PG 16:0_16:0		[M-H]-
41	PG 16:0_18:1		[M-H]-
42	PG 16:0_18:2		[M-H]-
43	PG 16:0_20:4		[M-H]-
44	PG 16:0_22:6		[M-H]-
45	PG 17:0_17:0		[M-H]-
46	PG 18:0_18:0		[M-H]-
47	PS 14:0_14:0	[M+H]+	[M-H]-
48	PS 16:0_16:0	[M+H]+	[M-H]-
49	PS 16:0_18:1	[M+H]+	[M-H]-
50	PS 16:0_18:2	[M+H]+	[M-H]-
51	PS 16:0_20:4	[M+H]+	[M-H]-
52	PS 16:0_22:6	[M+H]+	[M-H]-
53	PS 17:0_17:0	[M+H]+	[M-H]-
54	PS 18:0_18:0	[M+H]+	[M-H]-

Table S1.9: *Phospholipid standards: spectra counts per vendor at each processing stage.*

Vendor	Raw MS2	After processing	High quality	Lost (quality)	% retained
Agilent	9,894	9,751	9,382	369	94.8%
Bruker	27,888	27,888	24,412	3,476	87.5%
Sciex	50,793	50,732	46,585	4,147	91.7%
**Total**	**88,575**	**88,371**	**80,379**	**7,992**	**90.7%**

S1.5 Final Merge

The four processed HDF5 files (phospholipid standards, mitomics, GNPS, Thermo) were concatenated into the final training dataset with no additional filtering. The only post-merge processing was a fatty acid sorting step to ensure consistent side chain ordering across all sources. The final merged dataset contains 268,731 spectra covering 2,303 unique molecular lipid species. This merged dataset was saved as a single HDF file so that the preprocessing steps only had to be performed once and to allow for easy and fast data reading during training. Each spectrum was saved as an HDF5 dataset with the corresponding molecular lipid species, adduct, and precursor mass as metadata attributes.

Table S1.10: *Number of lipid species and spectra for each data source as well as average number of peaks per spectrum per source.*

Source	Nr Lipid Species	Nr Spectra	Mean Nr Peaks
HCE	100	113	285
Thermo	936	28039	18
IOBA	142	195	324
MITOMICS	430	113654	21
Standards	54	80379	4310
PNNL	1631	46340	107

Table S1.11: *Number of lipid species per lipid class for each data source.*

Lipid Class	HCE	Thermo	IOBA	MITOMICS	Standards	PNNL
CAR	0	0	0	607	0	0
CE	0	0	0	275	0	64
CL	0	0	0	1739	0	483
Cer	16	358	2	12962	0	2691
DG	0	259	0	8	0	1674
DGDG	0	0	0	0	0	320
EPC	0	0	0	0	0	2
FA	12	0	0	0	0	0
GalCer	2	0	0	0	0	838
GlcCer	0	0	0	0	0	6
HexCer	3	123	4	0	0	0
IPC	0	0	0	0	0	120
LPC	0	0	0	9894	0	2760
LPE	1	861	0	22675	0	2016
LPG	0	26	0	2238	0	70
LPI	0	146	0	121	0	394
LPS	0	64	0	0	0	144
LacCer	1	0	0	0	0	66
MG	0	0	0	0	0	12
MGDG	0	0	0	0	0	42
PA	1	0	0	250	6444	150
PC	16	7565	72	22003	31125	10153
PE	27	2544	49	12286	22696	6730
PG	5	628	4	27	7329	2880
PI	8	2242	10	4235	0	2736
PS	6	2524	9	811	12785	1716
SHexCer	0	2	0	0	0	0
SM	15	28	7	0	0	1881
SPB	0	0	0	0	0	100
SQDG	0	0	0	0	0	228
TG	0	10669	38	14185	0	8064

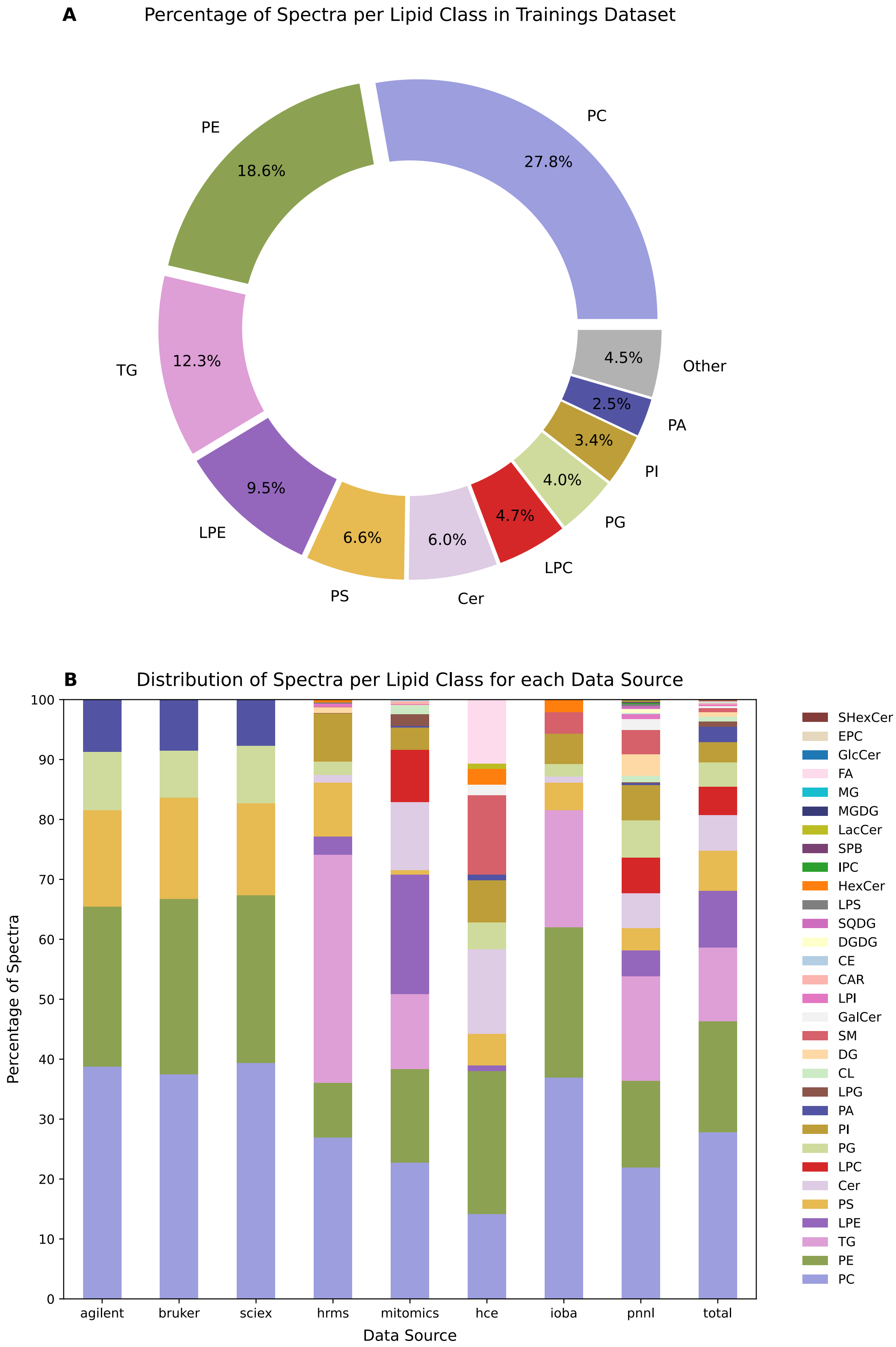

Figure S1.3: *A) Lipid class distribution of spectra in the training dataset. B) Distribution of lipid classes for each source. Variation between the Agilent, Sciex, and Bruker sources is due to the noisy spectra filtering as described in methods section 2.2.2.*

S1.6 Representativeness of training data

Clinical lipidomes are highly diverse, with recent studies quantifying hundreds of distinct molecular species across more than twenty lipid classes and demonstrating pronounced inter-individual variability and sex specificity [34]. Despite this extensive chemical diversity, lipid abundance distributions are strongly skewed: a limited number of classes, particularly cholesteryl esters (CE), phosphatidylcholines (PC), and triacylglycerols (TG), account for the majority of total circulatory lipid content, whereas many other classes occur at substantially lower concentrations. Inter-laboratory consensus measurements of NIST SRM 1950 plasma [6] similarly identify TAGs, PCs, SMs, PEs, and LPCs as the most consistently reported and quantitatively robust lipid classes across laboratories, see Supplement Figure S1.4. Thus, clinical lipidomes combine high molecular diversity with a strongly skewed abundance distribution dominated by a relatively small subset of lipid classes and recurrent acyl-chain motifs. The LipiDetective trainset is class-imbalanced, with >50% of spectra belonging to PC, PE, or TG species, qualitatively mirroring the dominance of these classes in plasma lipidomes.

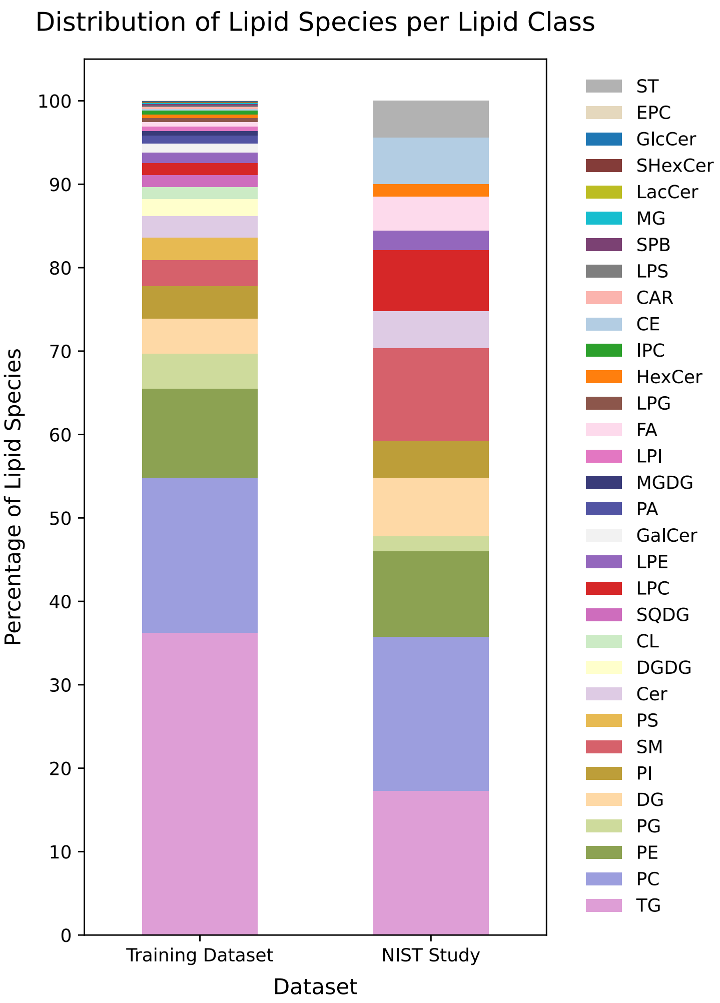

Figure S1.4: Comparison of lipid class distribution in training data vs NIST plasma identifications.

The datasets used to train LipiDetective already reflect a broad range of experimental conditions commonly encountered in lipidomics, including multiple mass spectrometer platforms, collision energies, ionization modes, and sample types. Public datasets such as MITOMICS and GNPS contain spectra derived from complex biological matrices, while the Thermo dataset contains measurements of multiple organs, such as heart, kidney, liver and spleen. However, we note that most spectra originate from research-grade experiments rather than routine clinical diagnostics, and that certain lipid classes such as free fatty acids remain underrepresented, limiting the model's suitability as a dedicated FA identification tool. Additionally, fatty-acyl frequencies are highly skewed. Under fragment ambiguity, this induces a statistical prior toward common acyl chains, explaining substitutions such as TG 22:5_22:6_22:6 being predicted as TG 16:0_22:6_22:6.

Environmental lipidomics experiments, such as those involving microbial communities, plant material, or soil and marine samples, are not explicitly represented in the current training data, as the focus of this study was on lipid species commonly

observed in human and mammalian systems. However, extending the model to environmental lipidomes represents a promising direction for future work. While environmental lipidomics experiments (e.g., microbial, plant, or marine samples) are not directly represented in the datasets used here, the underlying modeling framework is agnostic to sample origin, and we expect that incorporating spectra from environmental studies would further expand LipiDetective's coverage and robustness in future iterations. As such, LipiDetective should be viewed as a foundation model for lipid identification whose representativeness and performance are expected to improve as additional spectra from diverse clinical and environmental studies become available.

Overall, the training corpus is well aligned with clinically dominant lipid classes and canonical acyl chemistries, supporting robust learning of common fragmentation motifs. Nonetheless, three relevant expansion priorities emerge: (i) systematic inclusion of free fatty acids and oxidized lipid mediators as separate prediction tasks, (ii) targeted enrichment of spectra containing rare long-chain PUFA chains to mitigate substitution bias, and (iii) benchmarking strategies that explicitly distinguish between sum-level and molecular-species resolution. These considerations define a clear roadmap for dataset refinement.
