# Supplementary material for: LipiDetective: a deep learning model for the identification of molecular lipid species in tandem mass spectra
Source: Brief Bioinform. 2026 Jul 27;27(4):bbag378. doi: 10.1093/bib/bbag378 (PMC13403187; doi:10.1093/bib/bbag378)
Supplement: Supplementary-material_bbag378 [file supplementary-material_bbag378.zip › LipiDetective_Supplement_2_bbag378.pdf]

Supplement 2: Transformer Architecture Details

S2.1 Input Representation

The transformer encoder receives a fixed-length sequence of discretized *m/z* values extracted from a tandem mass spectrum. Peak intensities are discarded andthe model relies exclusively on fragmentation peak positions.

Preprocessing pipeline

Given a raw MS2 spectrum, the input is constructed as follows:

1. Peaks are binned by truncating *m/z* values to 0.1 Da resolution, summing intensities of coalescing peaks.
2. Peaks with scaled *m/z* $\geq 16{,}000$ are removed.
3. The top $n = 30$ peaks by intensity are selected.
4. The top 30 binned *m/z* values are scaled to integers: $\lfloor m/z \times 10 \rceil$, yielding values in $[0, 16{,}000)$.
5. If fewer than 30 peaks remain, the sequence is zero-padded on the right.
6. The precursor *m/z* (integer-scaled) is inserted at the last position, provided it is within range and not already present as a fragment peak.

The resulting encoder input is an integer tensor of shape $(B, 30)$, where B corresponds to the batch size.

Excluded metadata

No experimental metadata (charge state, collision energy, instrument type) is provided to the encoder. This deliberate minimalism reduces input requirements at inference time and discourages overfitting to acquisition-specific patterns. Instead, the model is encouraged to learn fragmentation regularities that generalize across platforms. Adduct type is predicted as part of the output sequence rather than supplied as input. This enables joint learning of structural identity and ionization mode, allowing the model to internally verify consistency between fragmentation pattern and adduct assignment.

S2.2 Model Architecture

LipiDetective employs a standard encoder-decoder transformer architecture. Table S2.1 summarizes the hyperparameters.

Table S2.1: *Transformer hyperparameters.*

Parameter	Value	Description
d_{model}	32	Embedding dimension
n_{heads}	4	Attention heads (head dim = 8)
n_{layers}	2	Encoder and decoder layers (each)
d_{ff}	256	Feedforward hidden dimension
Dropout	0.1	Applied in attention and FFN
Encoder seq. length	30	Number of input peaks (n_{peaks})
Decoder seq. length	11	Output sequence length (incl. special tokens)
Encoder vocab size	16,000	*m/z* bins (max_mz $\times$ $10^{\text{decimal\_accuracy}}$)
Decoder vocab size	243	Lipid nomenclature tokens

S2.2.1 Encoder

The input vocabulary consists of discrete m/z tokens obtained by truncating fragment m/z values to one decimal place within a maximum m/z of 1600 and converting them to integer indices (e.g. 123.4 → 1234), yielding 16,000 possible input tokens (`nn.Embedding`(16,000„32)). This design choice highlights a fundamental trade-off between m/z precision and embedding quality: each additional decimal place increases the vocabulary size by an order of magnitude. Using two decimal places, for example, would expand the input vocabulary to approximately 160,000 distinct tokens, substantially reducing the frequency with which individual tokens are observed and weakening the statistical signal available for learning meaningful embeddings. With sufficiently large and diverse training data, higher m/z precision may become feasible or even advantageous; in the present setting, however, it would further fragment the input space and result in sparser token observations.

At inference time, fragment peaks above 1,600 m/z fall outside the embedding vocabulary and are discarded, as no learned representation exists for them. Below this threshold, every possible one-decimal m/z value maps to a valid index, so no unknown-token mechanism is required. The `InputEncoder` then multiplies the retrieved embeddings by $\sqrt{d_{\text{model}}}$ following [26], and adds fixed sinusoidal positional encodings of length n. The resulting representations are processed by a two-layer `TransformerEncoder`.

S2.2.2 Decoder

The output vocabulary of 243 tokens encodes lipid shorthand notation, comprising lipid class identifiers (e.g., PC, PE), fatty acid chains (carbon number and degree of unsaturation), ether linkage and functional group modifiers, adducts, separators, and special control tokens (`<SOS>`, `<EOS>`, `<PAD>`). Table S2.2 summarizes the vocabulary composition.

Target tokens are embedded via a separate `nn.Embedding`$(243, 32)$ with sinusoidal positional encodings of length 11 and processed by a two-layer transformer decoder that attends to the encoder output via cross-attention. A final linear projection ($32 \rightarrow 243$) maps decoder hidden states to logits over the output vocabulary.

Table S2.2: *Output vocabulary composition (243 tokens).*

Indices	Category	Count	Examples
0–2	Special tokens	3	`<PAD>`, `<SOS>`, `<EOS>`
3–4	FA separators	2	`_` (sn-position unknown), `/` (sn-position known)
5–16	Adducts	12	$[M+H]^+$, $[M\text{-}H]^-$, $[M+NH_4]^+$, $[M+H\text{-}H_2O]^+$, ...
17–66	Lipid classes	50	PC, PE, TG, SM, Cer, DG, LPC, ...
67–68	Ether linkages	2	O- (ether), P- (plasmalogen)
69–76	Functional groups	8	;O, ;O2, ;O3, ;O4, ;O5, ;S, ;G, ;T
77–242	Fatty acid chains	166	2:0 through 50:2 (carbon:double bonds)

A lipid name is parsed into structural components via regular expressions and mapped to token indices. The target sequence follows the pattern:

$$\texttt{<SOS>}\ \underbrace{\text{CLASS}\ \overbrace{\text{[BOND] FA [FG] SEP}}^{\times 1}\ \cdots\ \overbrace{\text{[BOND] FA}_n\ \text{[FG]}}^{\times n}}_{\text{lipid name components}}\ \text{ADDUCT}\ \texttt{<EOS>}\ \underbrace{\texttt{<PAD>}\ \cdots}_{\text{to length 11}}$$

where brackets denote optional components. For example, `PC 16:0_18:1 [M+H]+` is tokenized as:

$$[1,\ 54,\ 127,\ 3,\ 142,\ 7,\ 2,\ 0,\ 0,\ 0,\ 0]$$

corresponding to `<SOS>` (1), `PC` (54), `16:0` (127), `_` (3), `18:1` (142), `[M+H]+` (7), `<EOS>` (2), followed by four `<PAD>` (0) tokens.

No unknown (`<UNK>`) token exists. If the model cannot identify a spectrum, it will often predict the `<EOS>` token immediately, yielding an empty prediction. The vocabulary is a fixed set covering all lipid species, adducts, and structural motifs present in the training data. At inference, the decoder generates tokens exclusively from this closed set; this means LipiDetective cannot generate tokens absent from the output vocabulary; however, it can produce novel lipid species combinations by recombining known fatty acid and class tokens, enabling generalization to unseen lipid species. Chemically implausible combinations are discouraged through learned token co-occurrence patterns rather than explicit constraints.

S2.2.3 Padding and Masking

MS2 spectra vary in the number of detected fragment peaks. After selecting the top n peaks, spectra with fewer than n peaks are padded using a <PAD> token. Standard attention masks are applied to prevent padded positions from contributing to the attention computation in both encoder and decoder layers.

During training, the full label sequence of length 11 (`<SOS>`, lipid tokens, `<EOS>`, and padding) is split into a decoder input `tgt_input` = `labels[:, :-1]` (positions 0-9, excluding `<EOS>`) and a prediction target `tgt_expected` = `labels[:, 1:]` (positions 1-10, excluding `<SOS>`). The decoder therefore operates on sequences of length 10.

Three masks govern attention during the forward pass:

1. **Source padding mask** (shape $B \times 30$): `True` where *m/z* $= 0$ (padded positions). Passed to both the encoder's `src_key_padding_mask` and the decoder's `memory_key_padding_mask`, ensuring that neither encoder self-attention nor decoder cross-attention attend to padded peaks.
2. **Target padding mask** (shape $B \times 10$): `True` where the decoder input token is `<PAD>` (index 0). Used only during teacher-forced training.
3. **Causal mask** (shape 10×10): Upper-triangular boolean matrix preventing the decoder from attending to future positions. Used only during teacher-forced training; unnecessary during autoregressive inference.

S2.3 Training and Inference

PyTorch Lightning [35] was used for multi-GPU training on a computing cluster. The cluster consists of multiple compute nodes equipped with two or three GPUs. The specific GPU models vary across nodes, including NVIDIA's Titan Xp, Titan V, and A40.

The model is trained with teacher forcing using cross-entropy loss. Optimization uses Adam with an initial learning rate of 4×10^{-3}, decayed by a factor of 0.9 every 2 epochs via `StepLR`, over 15 epochs with a batch size of 512.

Predictions are generated autoregressively using beam search with a beam width of 3:

1. The encoder processes the input spectrum once, producing a memory tensor.
2. The decoder is seeded with `<SOS>` (token 1).
3. At each of the 10 subsequent positions, the decoder produces a probability distribution over the 243-token vocabulary. Three candidate beams are maintained and expanded at each step, then pruned back to the top 3 by cumulative log-probability.
4. The beam with the highest total log-probability is selected as the final prediction.

A *top-3* variant returns all three beam candidates with their probabilities, used for providing ranked alternative identifications.
