# Supplementary material for: LipiDetective: a deep learning model for the identification of molecular lipid species in tandem mass spectra
Source: Brief Bioinform. 2026 Jul 27;27(4):bbag378. doi: 10.1093/bib/bbag378 (PMC13403187; doi:10.1093/bib/bbag378)
Supplement: Supplementary-material_bbag378 [file supplementary-material_bbag378.zip › LipiDetective_Supplement_3_bbag378.pdf]

Supplement 3: Ablation Studies and Design Rationale

To systematically evaluate how each design decision contributes to LipiDetective's predictive performance, we conducted a series of ablation studies spanning model architecture, preprocessing, training data composition, and cross-instrument generalization. Together, these experiments characterize the sensitivity of the model to its key hyperparameters, quantify the robustness of predictions across random initializations and instrument platforms, and provide empirical justification for the architectural and preprocessing choices described in the main text. Additionally, this section discusses the alternatives considered and the reasoning behind the chosen design.

S3.1 Architecture Parameters

We performed a comprehensive hyperparameter search using Ray Tune with the ASHA early stopping scheduler across 375 configurations. Eight hyperparameters were varied simultaneously: embedding dimension (d_{model}), number of attention heads, number of transformer layers, feed-forward hidden dimension, dropout rate, learning rate, batch size, and number of input peaks. All runs used the same 100-spectra-count validation split and a maximum of 25 training epochs. ASHA terminated 222 underperforming runs early (typically at epoch 10), concentrating compute on the 159 most promising configurations. The complete results table for all 375 runs is provided in Supplement 6 nd Figure S3.2 shows the per-run accuracy distributions stratified by each hyperparameter.

Larger batch sizes (256-512), moderate dropout (0.1), higher learning rates (0.003-0.004), and larger feed-forward dimensions (256-512) consistently yielded the highest accuracies. Very small embedding dimensions ($d_{\text{model}} \leq 16$) and deep architectures (6 layers) were predominantly terminated early, confirming that these configurations are unsuitable for this task. The best overall configuration achieved 32.4% validation accuracy with $d_{\text{model}} = 72$, 4 attention heads, 2 layers, $d_{\text{ffn}} = 512$, dropout 0.1, learning rate 0.001, batch size 256, and 70 input peaks. The top configurations showed similar accuracy within a narrow range of approximately 2 percentage points, suggesting that model performance has begun to saturate with respect to architectural choices. Further improvements likely depend more on training data diversity and coverage than on architecture.

The production model ($d_{\text{model}} = 32$, 4 heads, 2 layers) achieved 32.3% validation accuracy (within 0.1 percentage points of the best configuration) while attaining a lower validation loss (0.383 vs. 0.387). Despite using an embedding dimension 2.25× smaller, it ranks second overall, representing a favorable trade-off between model compactness and predictive performance. We therefore retained this architecture for all subsequent experiments.

Table S3.1: *Hyperparameter values chosen after tuning and used for all presented runs.*

Hyperparameter	Optimum
Learning rate	0.004
Learning rate step	2
Dropout	0.1
Batch size	512
Epochs	15
Number of peaks	30
Decimal places	1
Embedding dimension	32
Number of heads	4
Number of layers	2
Dimension of Feedforward Network	256

Accuracy-Loss Discrepancy

We observed that the architectures with the lowest validation loss did not always achieve the highest validation accuracy (Figure S3.1). This discrepancy arises because the validation accuracy metric is a lipid-specific discrete measure (exact match of predicted nomenclature), while the loss is a continuous per-token cross-entropy. A model can improve its per-token probability estimates (lower loss) without necessarily producing more correct complete lipid predictions (higher accuracy). The best configuration ($d_{\text{model}} = 72$, 4 heads, 2 layers) achieves 32.4% validation accuracy, while the production model reaches 32.3% with a lower validation loss (0.383 vs. 0.387), demonstrating that the compact architecture matches the accuracy of substantially larger models.

S3.2 Input Representation

LipiDetective represents MS2 spectra as a set of discretized fragment masses rather than as continuous peak lists with associated intensities. Fragment *m/z* values are binned and mapped to learned embedding vectors. This tokenized formulation allows the model to learn position-specific mass representations (analogous to word embeddings) rather than relying on a single linear projection of floating-point inputs. The 0.1 Da resolution balances mass accuracy typical of low-to mid-resolution instruments with vocabulary tractability.

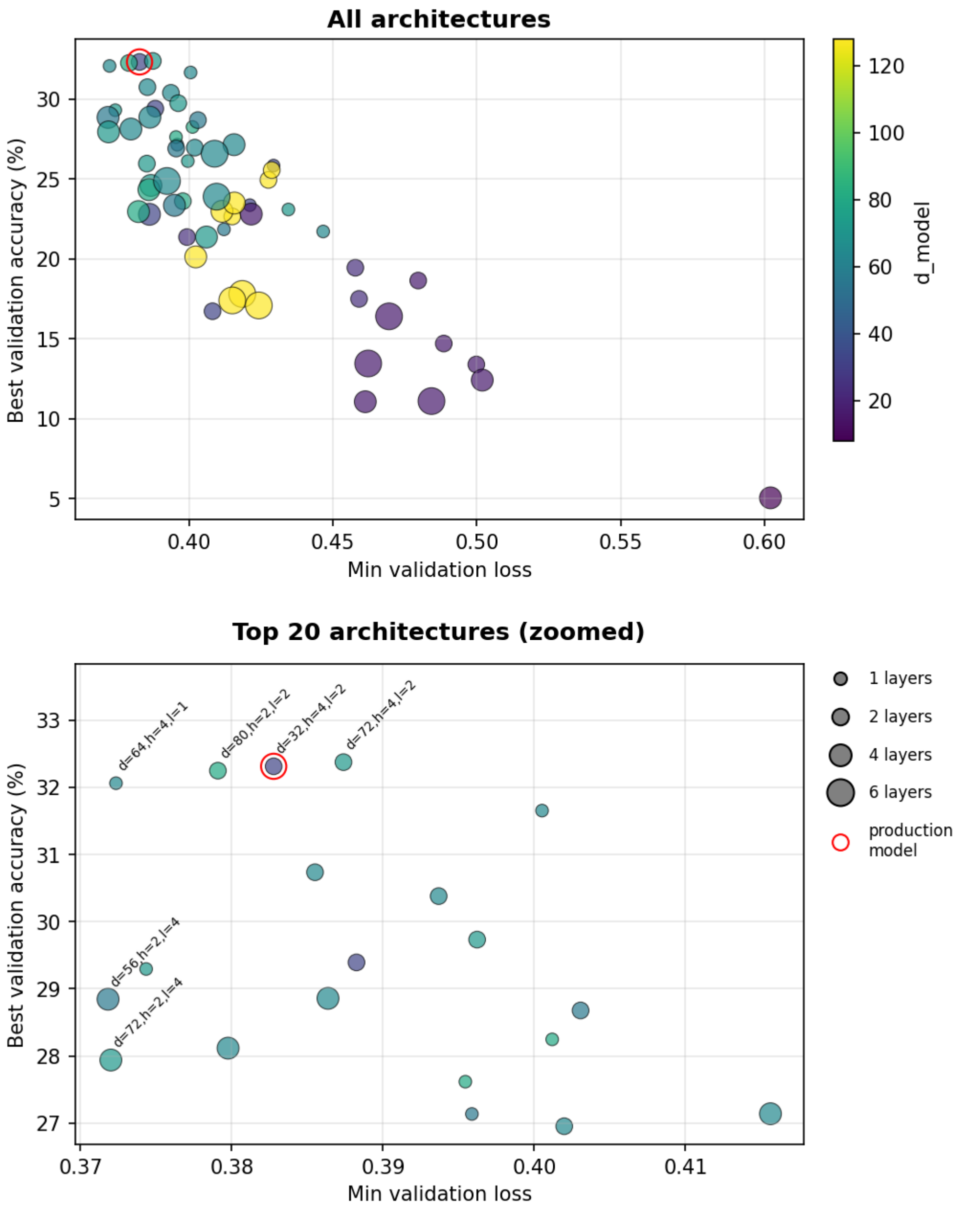

Figure S3.1: *Architecture ablation: validation accuracy versus validation loss across 375 hyperparameter configurations. Each point represents one architecture configuration, with marker size indicating the number of transformer layers, color encoding the embedding dimension (d_{model}), and the production model ($d_{\mathrm{model}} = 32$, 4 heads, 2 layers) highlighted in red. Top: Overview of all configurations. Bottom: Zoomed view of the top 20 configurations.*

Only fragment *m/z* positions are provided to the encoder; peak intensities are discarded after top-N selection. While intensities encode relative abundance, they vary substantially across instruments and collision energies. By focusing on fragmentation patterns—*which* bonds break rather than *how much* of each fragment is produced—the model becomes more robust to instrument-dependent intensity variation.

To evaluate the influence of these preprocessing hyperparameters systematically, we conducted ablation studies the decimal precision and number of input peaks (see Figure S3.3), keeping all other settings constant ($d_{\mathrm{model}} = 32$, 4 heads, 2 layers, 15 epochs, batch size 512, $k = 6$, seed 42).

S3.2.1 Number of input peaks

We varied the number of input peaks from 15 to 70, training separate models for each setting. Exact accuracy was relatively stable across 15-50 peaks, ranging from $18.7 \pm 1.5\%$ (35 peaks) to $22.2 \pm 0.7\%$ (20 peaks), while component accuracy remained between 65.4% and 66.7%. At 70 peaks, however, performance collapsed entirely (0% exact, $15.9 \pm 2.0\%$ component), indicating that the model fails to learn meaningful representations when the input is dominated by low-intensity noise peaks. The production model uses 30 peaks, which achieved $22.2 \pm 0.6\%$ exact accuracy (component: $66.7 \pm 0.2\%$) and represents a stable operating point before the noise-dominated regime.

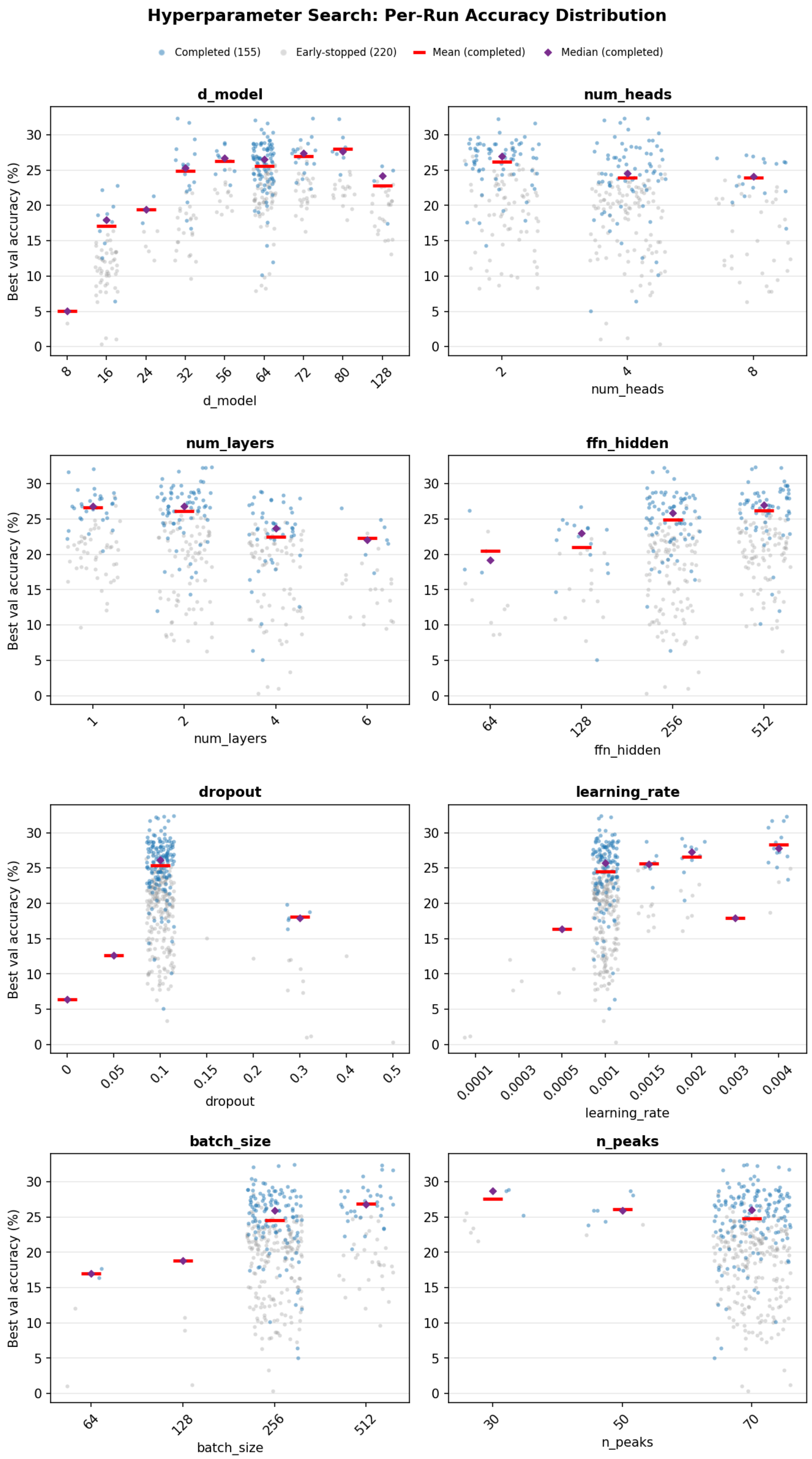

Figure S3.2: Per-run validation accuracy distributions across eight hyperparameters varied during the architecture search (375 runs total). Each dot represents one run's best validation accuracy across all training epochs. Blue dots indicate runs that completed all 25 epochs (155 runs); grey dots indicate runs terminated early by the ASHA scheduler (220 runs), which halts underperforming configurations at epoch 10 to concentrate compute on promising candidates. Red dashes and purple diamonds mark the mean and median accuracy of completed runs, respectively, providing a summary of converged performance unbiased by early stopping.

S3.2.2 Decimal precision

We varied the m/z discretization from 0 to 3 decimal places, corresponding to input vocabulary sizes of 1,600 to 1,600,000 tokens. Accuracy decreased monotonically with increasing precision: 0 decimal places achieved $26.0 \pm 1.0\%$ exact accuracy (component: $68.4 \pm 0.5\%$), while 1 decimal place achieved $21.2 \pm 0.8\%$ (component: $66.1 \pm 1.3\%$). Higher precision degraded rapidly: 2 decimal places dropped to $10.7 \pm 1.2\%$ exact (component: $57.9 \pm 0.4\%$), and 3 decimal places to $9.4 \pm 0.5\%$ (component: $53.9 \pm 0.4\%$). Each additional decimal place increases the vocabulary by an order of magnitude, fragmenting the input space and reducing the frequency with which individual tokens are observed. The production model uses 1 decimal place ($|V_{\text{enc}}| = 16{,}000$), which offers a practical compromise: it preserves sufficient m/z resolution for fragment discrimination while keeping the vocabulary tractable for embedding learning.

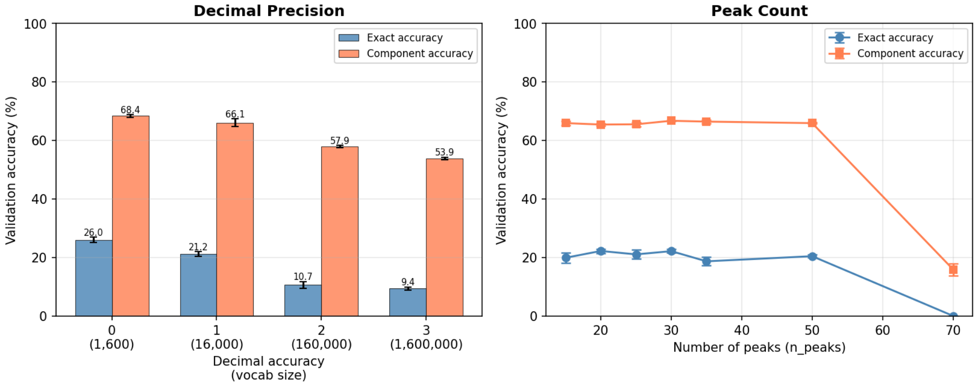

Figure S3.3: *Preprocessing ablation: effect of m/z decimal precision (**left**) and number of input peaks (**right**) on validation accuracy. Exact accuracy (blue) measures full lipid nomenclature matches; component accuracy (orange) measures the mean accuracy across individual lipid components. Values represent the mean of the last three training epochs; error bars indicate the standard deviation across those epochs. **Left:** Increasing the decimal precision expands the input vocabulary by an order of magnitude per decimal place (1,600 to 1,600,000 tokens), progressively fragmenting the embedding space and degrading accuracy. The production model uses 1 decimal place (vocabulary size 16,000). **Right:** Exact accuracy is stable across 15-50 input peaks (~19-22%), while 70 peaks causes a complete collapse (0% exact), indicating that including too many low-intensity noise peaks overwhelms the model. The production model uses 30 peaks.*

S3.3 Model Seed Variance

To assess run-to-run variance with a fixed validation split, we trained five models with identical architecture ($d_{\text{model}} = 32$, 4 heads, 2 layers) and hyperparameters, varying only the random seed (7, 42, 123, 256, 999). Each model was trained for 15 epochs on the training split and evaluated on the 100-spectra-lipid-species validation set (3,537 spectra). To reduce epoch-to-epoch fluctuations, we report the mean accuracy over the last three training epochs for each seed (Figure S3.4). Across the five seeds, exact-match accuracy was $21.2 \pm 1.1\%$ (mean $\pm$ std across seeds), and component accuracy was $68.1 \pm 0.9\%$. The low cross-seed standard deviation ($\sim$1 percentage point) indicates that model performance is robust to random weight initialization and training stochasticity.

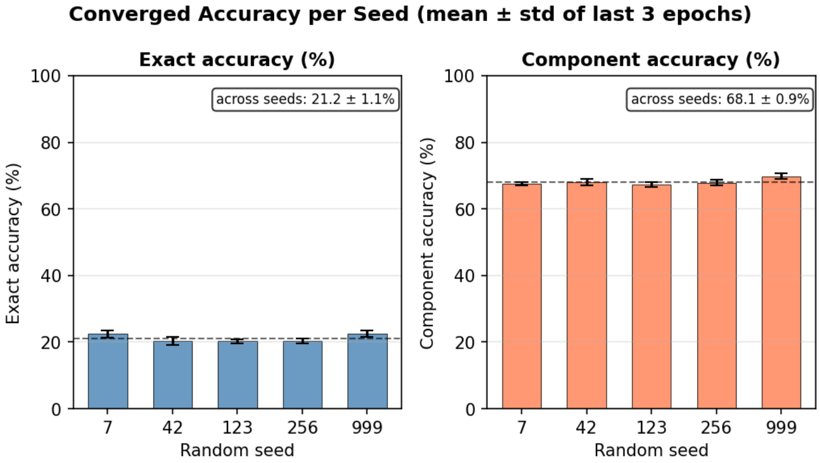

Figure S3.4: *Converged validation accuracy across five random seeds on the held-out validation split. Each bar represents one seed's mean accuracy over the last three training epochs; error bars indicate the standard deviation across those epochs, reflecting epoch-to-epoch stability at convergence. The dashed line marks the grand mean across all five seeds. **Left:** Exact-match accuracy (full lipid nomenclature match), $21.2 \pm 1.1\%$ across seeds. **Right:** Component accuracy (mean per-token accuracy across lipid name components), $68.1 \pm 0.9\%$ across seeds.*

S3.4 Comparison with MZmine on Phospholipid Standards

S3.4.1 Motivation

To contextualize LipiDetective's performance against an established tool, we compared its predictions with MZmine 4 [36, 37], a widely used open-source LC-MS data processing platform. MZmine was chosen for this comparison because of its accessibility, intuitive graphical interface, and well-documented lipid annotation workflow. Within minutes a user can install MZmine, load mzML files, and obtain lipid annotations. This comparison is not intended as a head-to-head benchmark but rather as a practical illustration of how a rule-based approach and a data-driven approach differ in their failure modes when applied to the same set of reference spectra.

A key challenge in computational lipidomics is the absence of a widely accepted gold-standard benchmark dataset containing confidently validated molecular lipid species across diverse classes and acquisition platforms. While public repositories such as MassBank [38] provide curated spectra, they are not designed to evaluate full workflow performance in realistic lipidomics discovery settings, nor do they allow direct comparison to standard library-driven identification pipelines applied to the same raw data. Furthermore, public repository spectra are often not available in the mzML format required by most processing tools, making it difficult to run competing methods on identical input.

For this comparison we therefore used the Agilent phospholipid standards dataset, which provides ground-truth molecular lipid identities, is available as mzML files compatible with both LipiDetective and MZmine, and covers five lipid classes across multiple instruments and collision energies. We used the LipiDetective model trained entirely without any Agilent data, ensuring a genuine leave-one-instrument-out evaluation rather than a comparison on data the model has already seen.

S3.4.2 Experimental Setup

Dataset

97 mzML files from Agilent QTOF measurements of 54 phospholipid standards (PA, PC, PE, PG, PS) across positive and negative ionization modes. Each file contains MS/MS spectra at multiple collision energies for a single lipid standard (direct infusion, not LC-MS). Ground-truth lipid identities are known from the standards.

LipiDetective

The transformer encoder-decoder model was trained on the full training set *excluding all Agilent data* (leave-one-instrument-out evaluation-the model has never seen any Agilent spectra during training). Per-scan predictions were obtained for all 9,751 MS/MS spectra and aggregated to per-standard majority votes for comparison at the file level.

MZmine 4

All 97 mzML files were loaded as a batch into MZmine 4. The default MZmine wizard settings were used (mass detection, chromatogram building, feature detection, alignment, lipid annotation via the "matchedlipid" method). A second run was performed with all lipid classes explicitly enabled in the lipid annotation module; results were identical to the default run. MZmine produces one annotation per detected chromatographic feature; features were mapped back to their source mzML files via the full feature table.

S3.4.3 Results

Table S3.2 summarizes the overall comparison across all 97 lipid standard files. LipiDetective achieves a 93.4% exact match rate (correct lipid class and side chain composition) compared to 18.3% for MZmine. In the disagreement analysis, there are 72 standards where LipiDetective is correct but MZmine is wrong or missing, and zero cases where MZmine is correct but LipiDetective is not.

Table S3.2: *Overall comparison of LipiDetective and MZmine on 97 Agilent phospholipid standard files.*

Metric	LipiDetective	MZmine
Standards with any annotation	91/97	71/97
Exact match (class + side chains)	85/97	13/97
Class-level match	88/97	53/97
Exact match rate (of annotated)	93.4%	18.3%

Table S3.3 shows the per-class breakdown. LipiDetective correctly identifies standards from all five lipid classes, while MZmine's rule-based annotation produces no correct identifications for PA or PS.

S3.4.4 Key Observations

LipiDetective identifies all five lipid classes, while MZmine's rule-based lipid annotation does not produce correct annotations for PA or PS in its default configuration. Even with all lipid classes explicitly enabled, PA and PS remain undetected.

Table S3.3: *Per-class comparison of LipiDetective and MZmine on 97 Agilent phospholipid standard files.*

Class	N	LipiDetective exact	MZmine exact	MZmine class	MZmine detected
PA	8	8/8	0/8	0/8	1/8
PC	38	31/38	7/38	26/38	30/38
PE	26	25/26	2/26	19/26	23/26
PG	9	9/9	4/9	8/9	9/9
PS	16	12/16	0/16	0/16	8/16

Of the 71 standards where MZmine detected a feature, only 13 received correct molecular-level annotations (exact class and side chains). In most cases MZmine identified the correct lipid class but assigned incorrect side chain compositions (e.g., PC 16:0_18:0 annotated as PC 18:0/18:0, or PE 16:0_20:4 annotated as PE 15:0/15:0).

MZmine annotates all PG standards as BMP, a structural isomer of PG. Since BMP and PG share the same molecular formula and produce similar fragmentation patterns, this is a known challenge for rule-based identification. LipiDetective correctly identifies all 9 PG standards, which comes with a caveat as LipiDetectives training data contains PG but not BMP spectra.

LipiDetective's failure modes are concentrated in specific conditions. It fails on PC 17:0_17:0 $[M+HCOO]^-$ (1% accuracy-an unusual odd-chain PC with formate adduct), PS 16:0_16:0 $[M-H]^-$ (0%), PS 16:0_18:2 in both polarities (0-2%), and PE 18:0_18:0 $[M+H]^+$ (16%). These failures correspond to species or adduct combinations underrepresented in training. The following section examines whether these failures can be attributed to low spectral quality or to systematic model limitations.

S3.4.5 Error Analysis and Spectral Quality

To understand whether LipiDetective's mispredictions arise from low-quality input spectra or from systematic model limitations, we analyzed the 1,017 incorrect predictions (10.4% of 9,751 total) along two dimensions: the type of error made and the spectral quality of the underlying MS/MS scan.

Error categories

Table S3.4 categorizes all mispredictions by error type. The dominant failure mode is predicting the correct lipid class but assigning incorrect side chains (42.9% of errors), typically differing by one degree of unsaturation (e.g., 18:2→18:1). The second most common failure is an empty prediction (16.9%), where the model's beam search does not produce a valid output-effectively an abstention. Notably, 9.3% of errors are *lyso-lipid predictions*, where the model predicts a single-chain lipid species (e.g., LPC 16:0) instead of the correct two-chain species (e.g., PC 16:0_20:4). Since lyso-lipids have substantially lower precursor masses than their diacyl counterparts (∼496 Da for LPC 16:0 vs. ∼782 Da for PC 16:0_20:4), these errors indicate that the model does not always leverage precursor mass information effectively. Headgroup confusion among structurally related classes (PA↔PG↔PS in $[M-H]^-$ mode) accounts for a further ∼10% of errors.

Table S3.4: *Categorization of LipiDetective's 1,017 mispredictions on the Agilent test set.*

Error type	Count	% of errors
Correct class, wrong side chains	436	42.9
Empty prediction (model abstains)	172	16.9
Lyso-lipid prediction (lost one chain)	95	9.3
Wrong headgroup class	267	26.3
Polarity/adduct mismatch	47	4.6

Spectral quality strongly predicts mispredictions

For each MS/MS scan, we extracted three quality indicators from the source data: the median normalized base peak intensity (NBPI; higher values indicate a noisier spectrum with more peaks of comparable intensity), the total ion current (TIC), and the number of raw peaks. As shown in Table S3.5, incorrectly predicted spectra are substantially noisier (13-31× higher NBPI by median and mean, respectively), weaker (2-6× lower TIC), and sparser (2-4× fewer peaks) than correctly predicted spectra. Error rates also increase with collision energy: 9.7% of spectra acquired at CE 10 eV are mispredicted, rising to 17.5% at CE 50 eV, consistent with over-fragmentation destroying diagnostic ions at high collision energies.

Standard-level analysis

Examining the worst-performing standards individually reveals that most failures are explained by poor spectral quality:

Table S3.5: Spectral quality comparison between correctly and incorrectly predicted spectra.

	NBPI		TIC		Raw peaks	
	Median	Mean	Median	Mean	Median	Mean
Correct (n = 8,734)	4.7×10^{-5}	5.5×10^{-4}	3.1×10^{6}	16.8×10^{6}	4,281	5,923
Wrong (n = 1,017)	6.2×10^{-4}	1.7×10^{-2}	0.5×10^{6}	7.4×10^{6}	991	3,046
Ratio (wrong / correct)	13×	31×	0.17×	0.44×	0.23×	0.51×

- PS 16:0_16:0 $[M-H]^-$ (0% accuracy): all 102 spectra have an NBPI of ∼0.048 (approximately 1,000× higher than the dataset median), indicating uniformly noisy spectra. The model produces empty predictions for every scan, effectively recognizing that no reliable identification is possible.
- PC 17:0_17:0 $[M+HCOO]^-$ (1% accuracy): NBPI of ∼0.067 across all spectra. This odd-chain PC with formate adduct combines an unusual lipid species with uniformly poor spectral quality, and the model's predictions scatter across unrelated lipid classes (PI, CL, PS).
- PC 22:0_22:0 $[M+HCOO]^-$ (41% accuracy): similarly high NBPI of ∼0.068. Very long-chain fatty acids (22:0) are rare in the training data, and the noisy spectra provide insufficient diagnostic information.

However, not all failures are quality-driven:

- PC 18:0_18:2 $[M+H]^+$ (60% accuracy): both correct and incorrect predictions come from spectra with near-identical, excellent quality (NBPI $\sim 10^{-6}$). Of the 41 errors, 34 predict PC 18:0_18:1 instead of PC 18:0_18:2 - a single-unsaturation confusion that reflects a genuine model limitation rather than input quality.
- PE 18:0_18:0 $[M+H]^+$ (16% accuracy): all 32 wrong predictions confidently (0.979 average confidence) identify PE 17:0_17:0. While the wrong spectra are noisier (28× higher NBPI), the systematic nature and high confidence of the error suggest the model has learned a spurious association rather than simply failing on noisy input.

In summary, the majority of LipiDetective's mispredictions on this dataset can be attributed to low spectral quality (noisy, weak, or over-fragmented spectra) that would challenge any identification approach. A smaller subset of errors reflects genuine model limitations, particularly in resolving closely related fatty acid side chains differing by a single degree of unsaturation and in fully leveraging precursor mass information to distinguish diacyl from lyso-lipid species.

S3.4.6 LipiDetective as Quality Control for Rule-Based Annotations

Beyond standalone comparison, LipiDetective can serve as a quality control layer for rule-based annotation pipelines such as MZmine. To evaluate this, we compared the per-standard majority-vote prediction from LipiDetective against MZmine's annotation for each of the 97 lipid standard files and classified every standard into one of six scenarios (Table S3.6).

Table S3.6: Outcome when LipiDetective is used to flag or supplement MZmine annotations. "Agree" means LipiDetective's majority-vote prediction matches MZmine's annotation at the molecular species level (class and side chains, treating BMP as PG).

MZmine status	Scenario	N
Annotated (n = 65)	Both agree, both correct	13
	LD disagrees, MZmine wrong (true flag)	52
	LD disagrees, MZmine correct (false flag)	0
Missing (n = 32)	LD fills gap correctly	23
	LD fills gap incorrectly	1
	Both tools missing	8

Implications for practical workflows

On this dataset, the two tools make entirely non-overlapping errors: MZmine's misannotations arise from incomplete fragmentation rule coverage (no rules for PA or PS) and limited side-chain resolution in its rule-based matching, while LipiDetective's errors concentrate on low-quality spectra and rare species that MZmine typically does not annotate at all. As a result, the 13 standards where both tools agree are all correctly identified, and LipiDetective correctly fills 23 of the 32 annotation gaps left by MZmine.

It is important to note that this analysis uses single-lipid-per-file standards, where per-file majority voting is straightforward. In a typical LC-MS experiment, each file contains hundreds or thousands of different lipid species, and the comparison would operate at the level of individual MZmine features matched to individual LipiDetective predictions for the corresponding MS/MS scans. Nevertheless, the core principle demonstrated here transfers: because rule-based and data-driven approaches have complementary failure modes, their agreement on a given identification provides a stronger signal of correctness than

either tool alone. One possible workflow would be to run both tools on the same dataset, retain all annotations on which they agree as high-confidence identifications, flag all disagreements for manual review, and use LipiDetective to provide candidate annotations where MZmine produces no identification. This would allow users to focus their manual validation effort on the subset of ambiguous cases rather than reviewing the entire annotation list.

S3.4.7 Limitations of This Comparison

Several important caveats apply to this comparison:

1. **MZmine is designed for LC-MS data with chromatographic separation.** These direct-infusion standard files lack retention time information, which means MZmine's chromatogram building and feature detection operate outside their intended use case. MZmine's performance on proper LC-MS lipidomics data with chromatographic separation would likely be substantially better.
2. **Default settings were used.** An expert MZmine user with optimized parameters, custom fragmentation libraries, or additional post-processing steps might achieve better results. We deliberately used default settings to represent the out-of-the-box experience, but this may underestimate MZmine's full capabilities.
3. **MZmine and LipiDetective solve fundamentally different problems.** MZmine is a complete LC-MS processing pipeline (peak picking, alignment, quantification, annotation), while LipiDetective is a specialized MS/MS-to-lipid-identity predictor. MZmine's lipid annotation is one module within a much larger workflow; comparing only the annotation step does not reflect MZmine's value as an integrated platform.
4. **LipiDetective has a training-data advantage.** While Agilent data was held out, the model has seen these lipid species from other instruments (Bruker, Sciex), giving it an advantage over a purely rule-based tool that must match fragmentation patterns without prior exposure to similar spectra.
5. **Standards do not reflect biological complexity.** The test set consists exclusively of single-lipid standards with known identities. In real biological samples, co-elution, isobaric interferences, and complex matrices present additional challenges that neither tool's performance on standards can predict.

S3.4.8 Conclusion

On this dataset of phospholipid reference standards, LipiDetective substantially outperforms MZmine's default lipid annotation in both coverage (91 vs. 71 standards annotated) and accuracy (93.4% vs. 18.3% exact match rate). The most striking differences are LipiDetective's ability to identify PA and PS-which MZmine's fragmentation rules do not cover, and its superior side-chain resolution.

Importantly, the two tools make fundamentally different types of errors: MZmine's misannotations arise from feature-level aggregation and missing fragmentation rules, while LipiDetective's failures concentrate on low-quality spectra and rare species. Because these failure modes are largely non-overlapping, agreement between the two tools provides a substantially stronger signal of correctness than either tool alone. On this dataset, the 13 standards where both tools agree are all correctly identified, and using LipiDetective to fill gaps where MZmine produces no annotation recovers 23 additional correct identifications. This suggests that LipiDetective could serve as a complementary validation layer within existing lipidomics workflows.

However, this comparison should be interpreted with the caveats listed above: MZmine is a versatile LC-MS platform not optimized for this specific evaluation scenario, and its ease of use and broad functionality make it an invaluable tool for the lipidomics community regardless of this narrow comparison.

In general, we advocate for the creation of a community-maintained, open gold-standard benchmark dataset for lipid identification covering diverse lipid classes, adduct types, instrument platforms, and collision energies in a standardized format with validated annotations and metadata. Such a resource would enable systematic, reproducible comparison of all lipid identification tools, accelerating progress in the field.

S3.5 Comparative Evaluation of Neural Network Architectures

The development of LipiDetective involved a systematic exploration of different neural network architectures, each representing a distinct approach to the problem of lipid identification from tandem mass spectra. This iterative process informed the design decisions that led to the current transformer-based encoder-decoder architecture. Below, we describe the architectures evaluated and provide a rationale for the final model choice.

S3.5.1 Problem Formulation

Three fundamentally different problem formulations were explored for predicting molecular lipid species from MS2 spectra:

1. **Regression**: The model predicts three continuous mass values corresponding to the headgroup and two fatty acid side chains. The predicted masses are then mapped to the nearest known lipid component to determine the lipid species.

2. **Classification**: The model treats lipid identification as a discrete label prediction task. Two variants were explored. Variant 1: Single multi-class model directly predicts the complete molecular lipid species as one class from a fixed vocabulary of all species in the training set. Variant 2: The problem is decomposed into three separate classification tasks: one model predicts the headgroup class, while two additional models independently predict the two fatty acid side chains. The final lipid species is then constructed by combining the predicted components.
3. **Sequence generation**: The model generates lipid nomenclature token by token using an encoder-decoder architecture with autoregressive decoding.

S3.5.2 Architectures Evaluated

Random Forest (RF)

As a non-deep-learning baseline, a random forest classifier was evaluated in two configurations: a single classifier predicting the full molecular lipid species, and a triple classifier predicting the headgroup and two side chains independently. The input features consist of binned spectral intensities.

Feedforward Neural Network (FFN)

A fully connected feedforward network with three hidden layers (256, 128, 64 neurons) was implemented as the initial baseline model. The input consists of the n highest-intensity peaks represented as m/z-intensity pairs, concatenated with the precursor mass and polarity. The network outputs three normalized mass values via MSE regression. Hyperparameter optimization identified a learning rate of 0.001 with a batch size of 32 as optimal. The inclusion of a dropout layer (p=0.5) reduced overfitting when evaluating on held-out lipid species, but could not eliminate it entirely.

Convolutional Neural Network (CNN)

A three-layer CNN with 2D convolutions and max-pooling was implemented to exploit potential local patterns in the peak structure of mass spectra. The convolutional layers are followed by three fully connected layers, producing the same three-value regression output as the FFN. The CNN showed reduced overfitting compared to the FFN when validated on held-out lipid species, suggesting that the convolutional architecture captures spectral patterns more robustly.

Long Short-Term Memory Network (LSTM)

An LSTM-based encoder was implemented to process the spectral peaks as an ordered sequence. The architecture comprised three stacked LSTM layers with a hidden size of 256. Because a single representation of the entire spectrum was required rather than per-peak outputs, only the final hidden state was retained and passed to a linear layer for lipid species prediction. The top 30 highest-intensity peaks were first mapped to embedding vectors of dimension 64 before being provided to the LSTM.

Transformer Encoder-only

Several transformer-based architectures were evaluated for the regression and classification tasks, including encoder-only variants and the so-called tri-transformer approach. The tri-transformer architecture consisting of three independent transformer encoders, each trained to predict one specific component. The original transformer architecture consists of an encoder and a decoder component. As the lipid identification via regression or classification requires a single output rather than autoregressive sequence generation, the decoder module was not utilized. Instead, the encoded spectrum representation was passed to a feedforward network that directly predicted either the continuous masses (regression setting) or the discrete class labels (classification setting) corresponding to the headgroup and fatty acid side chains.

Transformer Encoder-Decoder

The transformer architecture forms the core of LipiDetective and has been described extensively. The encoder uses multi-head self-attention to process all spectral peaks simultaneously, learning which peaks are informative for identification. The decoder autoregressively generates lipid nomenclature tokens conditioned on the encoder's spectrum representation.

S3.5.3 Evaluation Protocol

To begin with, models were trained and evaluated on the phospholipid standards dataset, which provides a well-defined ground-truth reference suitable for initial pilot studies. The dataset is relatively balanced and comprises 54 phospholipid standards spanning five headgroup classes (PA, PC, PE, PG, PS). These standards were measured on multiple mass spectrometry platforms (Agilent, Bruker, Sciex), yielding 80,379 MS^2 spectra acquired across a range of collision energies.

To rigorously assess generalization to unseen lipid species, we employed lipid-species-level validation splits: 9 lipid species (approximately 2 per headgroup class) were held out entirely from training and used exclusively for validation. This ensures that the validation set contains only lipid species not seen during training, testing the model's ability to generalize learned fragmentation rules rather than memorize specific spectra.

The importance of this splitting strategy is demonstrated by a control experiment with the random forest classifier: when using a random split that allows spectra of the same lipid species to appear in both training and validation sets (data

leakage), the triple classifier achieves 93.5% accuracy. With proper lipid-species-level splits, accuracy drops to 6.1%, revealing that the apparent high performance was entirely due to memorization of collision-energy variants of the same lipid rather than learning of transferable fragmentation patterns (Table S3.7).

Table S3.7: *Effect of validation split strategy on random forest performance. "Leakage" denotes random splits where the same lipid species can appear in both train and validation sets. "Species-level" denotes splits where held-out lipid species are entirely absent from training data.*

Model	Leakage Split	Species-Level Split
RF Single Classifier	94.9%	0.0%
RF Triple Classifier	93.5%	6.1%
RF Triple Regressor	89.9%	0.02%

The random forest classifier's complete failure on unseen lipid species (0-6%) establishes that this task requires models capable of learning abstract fragmentation rules, not simply memorizing spectral patterns. The leakage control experiment (93.5% with random splits vs 6.1% with species-level splits) further underscores the importance of rigorous evaluation protocols in spectral identification tasks.

S3.5.4 Comparison LSTM vs Transformer

The initial comparison between LSTM and Transformer architectures was performed using 10-fold cross-validation with lipid-species-level splits on the same reference dataset of phospholipid standards. Table S3.8 shows the results across all evaluated architectures. Two cross-validation strategies were employed: splitting by collision energy (testing interpolation to unseen collision energies of known lipid species) and splitting by lipid species (testing generalization to entirely unseen lipids). All models achieved high accuracy (>84%) when validated on unseen collision energies of known lipid species, confirming that spectral patterns are sufficiently consistent across collision energies for interpolation and likely equates to leakage. Generalization to unseen lipid species proved substantially more challenging, with accuracies dropping to 12-53% depending on the architecture.

The LSTM performed worst in both settings (84.8% / 12.8%), while transformer-based models consistently outperformed it. The most significant improvement came from the tri-transformer architecture, which splits the prediction into three independent sub-problems (headgroup, side chain 1, side chain 2), each handled by a separate transformer encoder. This decomposition improved lipid species accuracy from 27.2% (single transformer) to 42.6%. Reformulating the tri-transformer output as classification rather than regression further improved performance to 53.5%, confirming that discrete prediction of lipid components is more effective than continuous mass regression for this task.

Table S3.8: *Architecture comparison via 10-fold cross-validation on the phospholipid standards dataset. Accuracy is reported as mean ± standard error of the mean across folds. All models use the regression formulation except where noted.*

Model	Collision Energy CV (%)	Lipid Species CV (%)
LSTM	84.8 ± 2.9	12.8 ± 2.8
Transformer	93.1 ± 2.5	27.2 ± 2.8
Transformer (CLS token)	95.1 ± 1.0	23.1 ± 4.7
Transformer (Light Attention)	83.7 ± 3.1	23.2 ± 5.7
BERT (pre-trained)	94.5 ± 1.5	26.8 ± 5.0
Tri-Transformer (regression)	94.8 ± 0.7	42.6 ± 5.5
Tri-Transformer (classification)	$\mathbf{98.6 \pm 0.2}$	$\mathbf{53.5 \pm 5.2}$

Direct architecture comparison within the LipiDetective framework.

To complement the earlier cross-validation results, we performed a direct comparison of the feedforward, convolutional, and transformer architectures within the current LipiDetective framework, using the same 54-species phospholipid standards dataset with identical lipid-species-level validation splits (9 held-out species). The feedforward and convolutional networks were evaluated using the regression formulation, while the transformer used the encoder-decoder architecture with autoregressive token generation. All models were trained with seed 42 and results are reported as the mean over the last three training epochs (30 epochs in total for FFN and CNN, 15 for the Transformer).

S3.5.5 Advantages of the Transformer Architecture

The progressive evaluation of architectures reveals several key insights that motivated the final design of LipiDetective:

Generalization is the primary challenge

All architectures readily learn to identify lipid species encountered during training-even the simple FFN achieves near-perfect accuracy when validated on unseen collision energies of known lipids. The critical differentiator is performance

Table S3.9: Direct architecture comparison on the phospholipid standards dataset. The transformer uses the sequence generation formulation (encoder-decoder with autoregressive decoding). The FFN and CNN use the regression formulation (predicting three component masses). All models were evaluated with identical lipid-species-level validation splits (9 held-out species from 54 total). Accuracy is averaged over the last 3 epochs of a single run with seed 42.

Model	Task	Parameters	Val Accuracy (%)
FFN (3 hidden layers)	Regression	92.5K	1.4
CNN (3 conv + 3 FC layers)	Regression	11.4K	0.2
Transformer Encoder-Decoder	Seq. generation	621.8K	19.8

on entirely unseen lipid species, where architectures diverge dramatically. This confirms that the central challenge in ML-based lipid identification is learning transferable fragmentation rules, not pattern matching against known spectra.

Problem decomposition improves generalization

The tri-transformer's improvement over the single transformer (42.6% vs 27.2% on unseen species) demonstrates that decomposing lipid identification into sub-problems-predicting headgroup and side chains independently-helps the model learn component-specific fragmentation rules that transfer better to novel lipid species. This insight informed the design of the current encoder-decoder architecture, where the token-based output vocabulary implicitly decomposes the lipid into its constituent components.

Attention-based encoders outperform recurrent and feedforward alternatives

MS2 spectra are inherently unordered sets of fragment ions with long-range dependencies between diagnostically relevant peaks (e.g., headgroup losses, fatty acid fragments, and precursor-related ions). The transformer encoder's self-attention mechanism is well suited to this setting: it captures relationships between distant peaks without assuming locality or sequential order, unlike CNNs (fixed local receptive fields) or recurrent models (sequential processing). This global context is critical for lipid identification, where the PC headgroup peak at 184 m/z may be located far from the corresponding fatty acid loss peaks. Furthermore, transformers naturally handle variable-length inputs and provide interpretable attention patterns that are valuable for model inspection in a chemical context.

Limitations of regression and classification

The regression formulation has an inherent limitation: it requires the predicted mass to be precise enough to distinguish between lipid components that differ by as little as $\sim$2 Da (e.g., fatty acids differing by one degree of unsaturation, such as FA 18:0 at 284.48 Da and FA 18:1 at 282.46 Da). Small prediction errors can therefore lead to incorrect nearest-neighbor assignments. Furthermore, both the regression and classification approaches assume a fixed output structure (in this case headgroup + two side chains). While this is suitable for many glycerophospholipids, extending the framework to more structurally complex lipids (e.g., triacylglycerols or cardiolipins) would require increasing the number of predicted components and, in the decomposed setting, introducing additional dedicated models. This increases architectural complexity, reduces efficiency, and does not scale well as structural diversity grows.

In the decomposed classification variant, where separate models independently predict the headgroup and individual fatty acid side chains, the models operate without knowledge of each other's predictions. Consequently, they cannot enforce global chemical consistency or precursor mass constraints, potentially resulting in combinations of components that are chemically implausible or incompatible with the observed precursor mass. Moreover, because each component is learned independently, class imbalance effects may be amplified, as frequently occurring fatty acids or headgroups can dominate individual predictors without being counterbalanced by joint structural context. Additionally, the single-label classification variant is restricted to lipid species observed during training, preventing prediction of unseen combinations of headgroups and fatty acids.

Sequence Generation as a Scalable Modeling Approach

In contrast, the sequence generation formulation directly produces the lipid nomenclature string token by token, allowing flexible modeling of varying structural compositions and enabling novel component combinations without architectural modifications. The transformer architecture offers three key advantages: (1) the self-attention mechanism can capture long-range dependencies between peaks across the full m/z range, (2) the encoder produces interpretable attention weights that correlate with chemically meaningful fragment peaks, and (3) the sequence generation formulation naturally scales to arbitrary lipid vocabularies.

The autoregressive generation of tokens, in which each token is conditioned on all previously generated tokens, enables the decoder to learn inter-component dependencies. For example after generating `Cer`, the model has learned to expect / as the separator and to anticipate functional groups consistent with the ceramide class. Framing lipid identification as autoregressive sequence generation rather than classification over a fixed set of known species enables *compositional generalization*: the model has the possibility of predicting lipid species not seen during training by recombining known structural components (e.g., a headgroup observed with one set of fatty acids applied to a novel combination). This

compositional approach also scales naturally with vocabulary expansion as adding a new fatty acid chain requires only a single new token rather than enumerating all species containing that chain.

Absolute fragment intensities vary strongly across instruments, collision energies, and matrix contexts. Encoding raw intensities could therefore introduce dependence on experimental scaling effects and increase the risk of learning instrument-specific artifacts. By encoding relative-intensity rank via positional ordering, the model captures fragment prominence while remaining invariant to absolute intensity differences, supporting generalization across acquisition settings.

Collectively, these considerations led to the adoption of the encoder–decoder transformer architecture with autoregressive decoding as the final design of LipiDetective.
