# Supplementary material for: LipiDetective: a deep learning model for the identification of molecular lipid species in tandem mass spectra
Source: Brief Bioinform. 2026 Jul 27;27(4):bbag378. doi: 10.1093/bib/bbag378 (PMC13403187; doi:10.1093/bib/bbag378)
Supplement: Supplementary-material_bbag378 [file supplementary-material_bbag378.zip › LipiDetective_Supplement_4_bbag378.pdf]

Supplement 4: Complementary results tables and figure

Table S4.1: *Number of different lipid species, lipid classes, and fatty acids in each validation split by spectra count.*

Val Split	Nr Lipid Species	Nr Lipid Classes	Nr Fatty Acids
10 Spectra	93	16	42
100 Spectra	36	14	22
500 Spectra	16	6	15
1000 Spectra	7	5	6

Table S4.2: *Last 10 predictions recorded for the validation run with the 100 spectra split.*

Nr	Prediction	Label	Component Accuracy	Correct
1	TG 14:0_14:0_16:0 [M+NH4]+	TG 14:0_16:0_16:0 [M+NH4]+	0.8	FALSE
2	PE P-18:0_20:3 [M+H]+	PE P-18:0_20:3 [M+H]+	1.0	TRUE
3	PC 18:1_22:6 [M+H]+	PC 18:1_22:5 [M+H]+	0.75	FALSE
4	PE O-18:0_18:1 [M-H]-	PE 17:1_18:1 [M-H]-	0.75	FALSE
5	TG 18:1_18:1_20:3 [M+NH4]+	TG 16:0_18:1_22:4 [M+NH4]+	0.6	FALSE
6	PG 18:1_20:4 [M-H]-	PG 18:1_20:4 [M-H]-	1.0	TRUE
7	DG 18:1_18:1 [M+NH4]+	DG 18:1_18:1 [M+NH4]+	1.0	TRUE
8	PE P-18:0_18:2 [M-H]-	PE 17:1_18:1 [M-H]-	0.5	FALSE
9	PG 18:1_20:4 [M-H]-	PG 18:1_20:4 [M-H]-	1.0	TRUE
10	DG 18:1_18:2 [M+NH4]+	DG 18:1_18:1 [M+NH4]+	0.75	FALSE

Table S4.3: *Last 10 mispredictions recorded for the validation run with the DG lipid class split.*

Nr	Prediction	Label	Component Accuracy
1	TG 18:3_18:3_21:0 [M+NH4]+	DG 18:2_18:4 [M+NH4]+	0.25
2	TG 18:1_18:2_22:0 [M+NH4]+	DG 18:1_18:2 [M+NH4]+	0.75
3	TG 20:5_20:5_20:5 [M+NH4]+	DG 20:5_20:5 [M+NH4]+	0.75
4	TG 18:1_18:2_18:2 [M+NH4]+	DG 18:1_18:2 [M+NH4]+	0.75
5	TG 18:1_18:2_18:2 [M+NH4]+	DG 18:1_18:2 [M+NH4]+	0.75
6	TG 18:1_18:2_19:1 [M+NH4]+	DG 18:1_18:2 [M+NH4]+	0.75
7	TG 8:0_18:1_28:2 [M+NH4]+	DG 18:1_18:2 [M+NH4]+	0.5
8	DGDG 18:1_18:2 [M+NH4]+	DG 18:1_18:2 [M+NH4]+	0.75
9	LPE 16:0 [M-H]-	DG 18:1_18:2 [M+NH4]+	0.0
10	Cer 18:0;O2/16:0	DG 18:0_18:1 [M+NH4]+	0.25

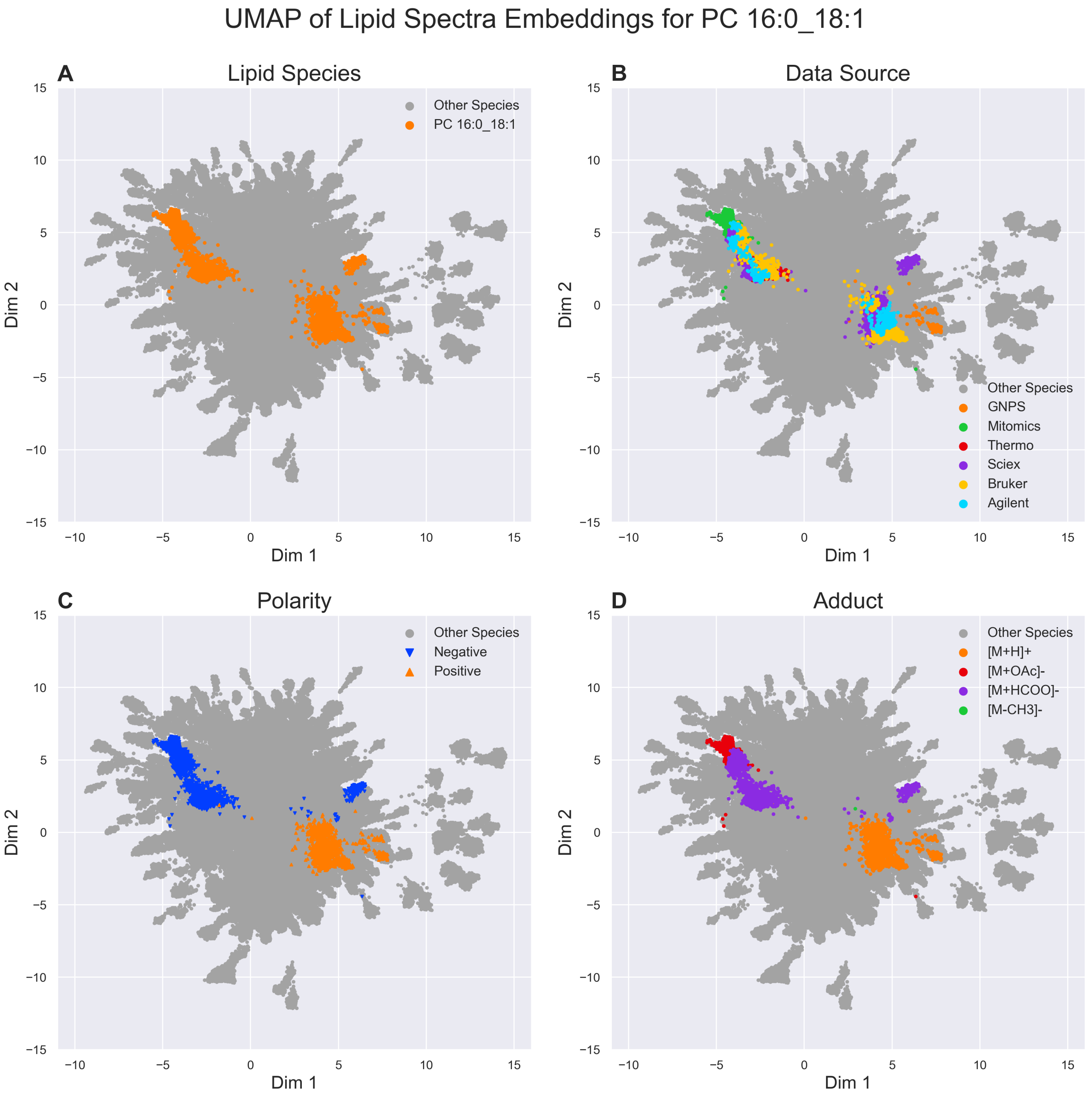

Figure S4.1: *UMAP of spectrum embeddings for A) PC 16:0_18:1 colored by B) source, C) polarity, and D) adduct.*

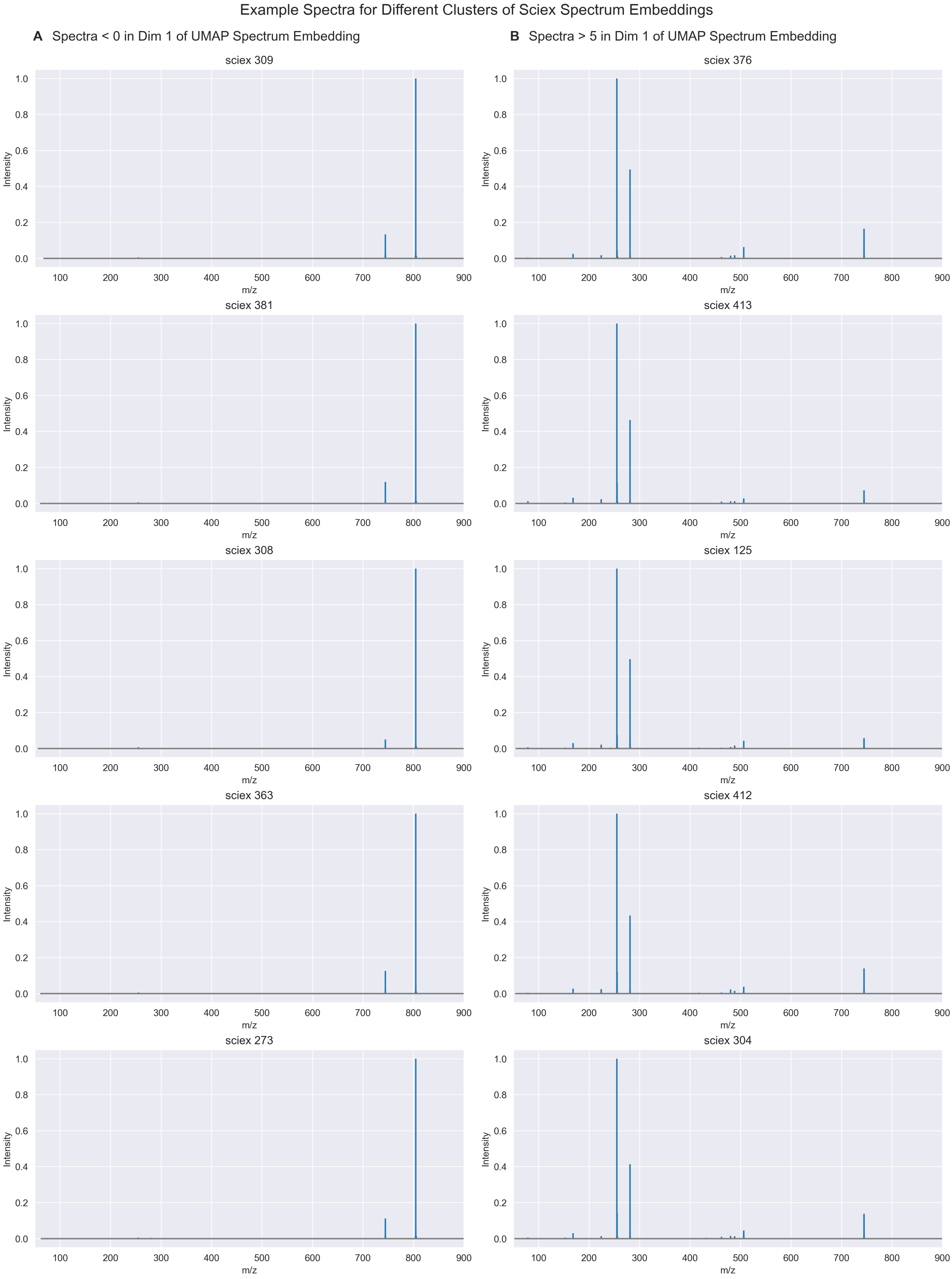

Figure S4.2: Example spectra for the two different sciex clusters in the UMAP embedding with A) Dim1 < 0 and B) Dim1 > 5.

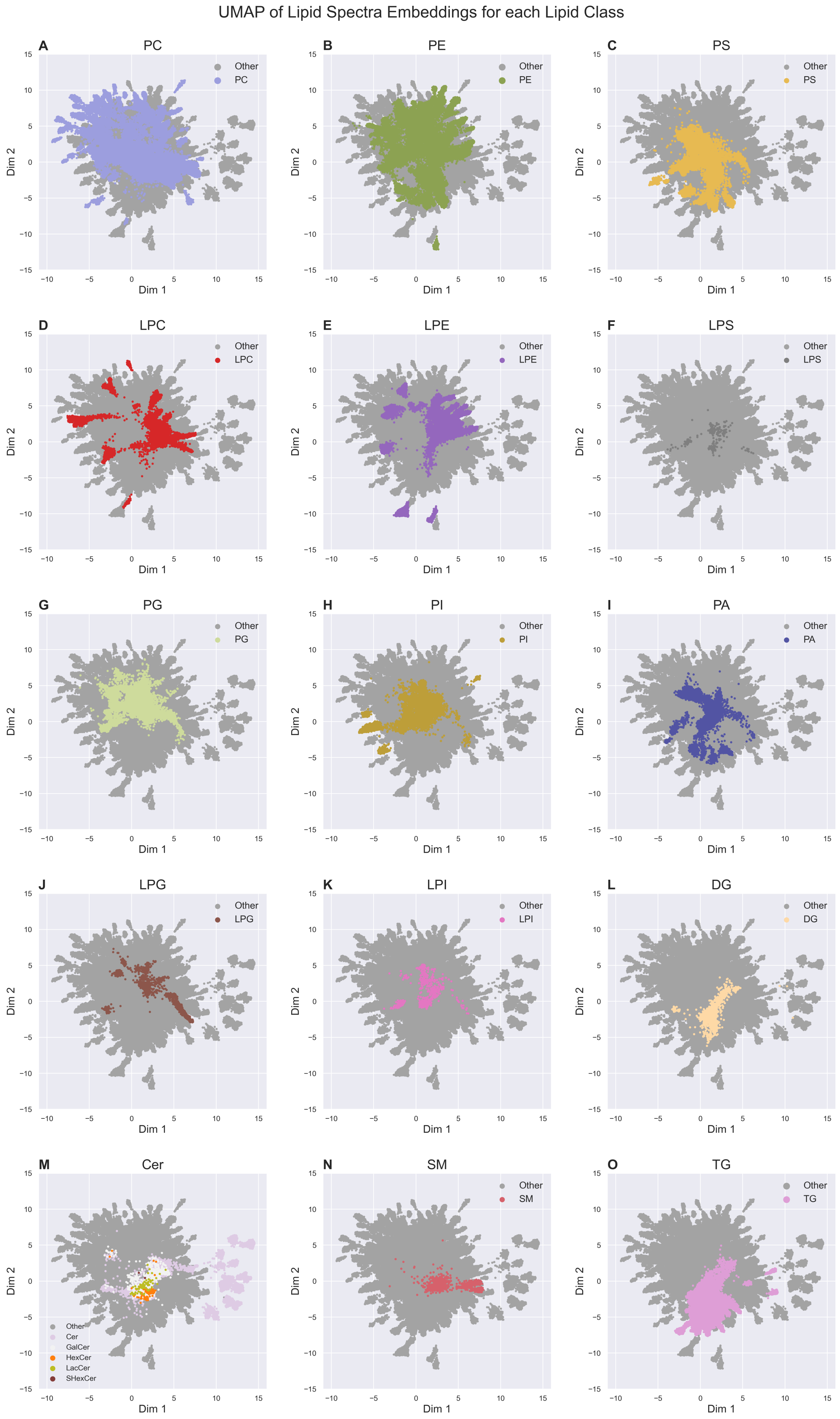

Figure S4.3: *Umap of embeddings colored by lipid classes.*

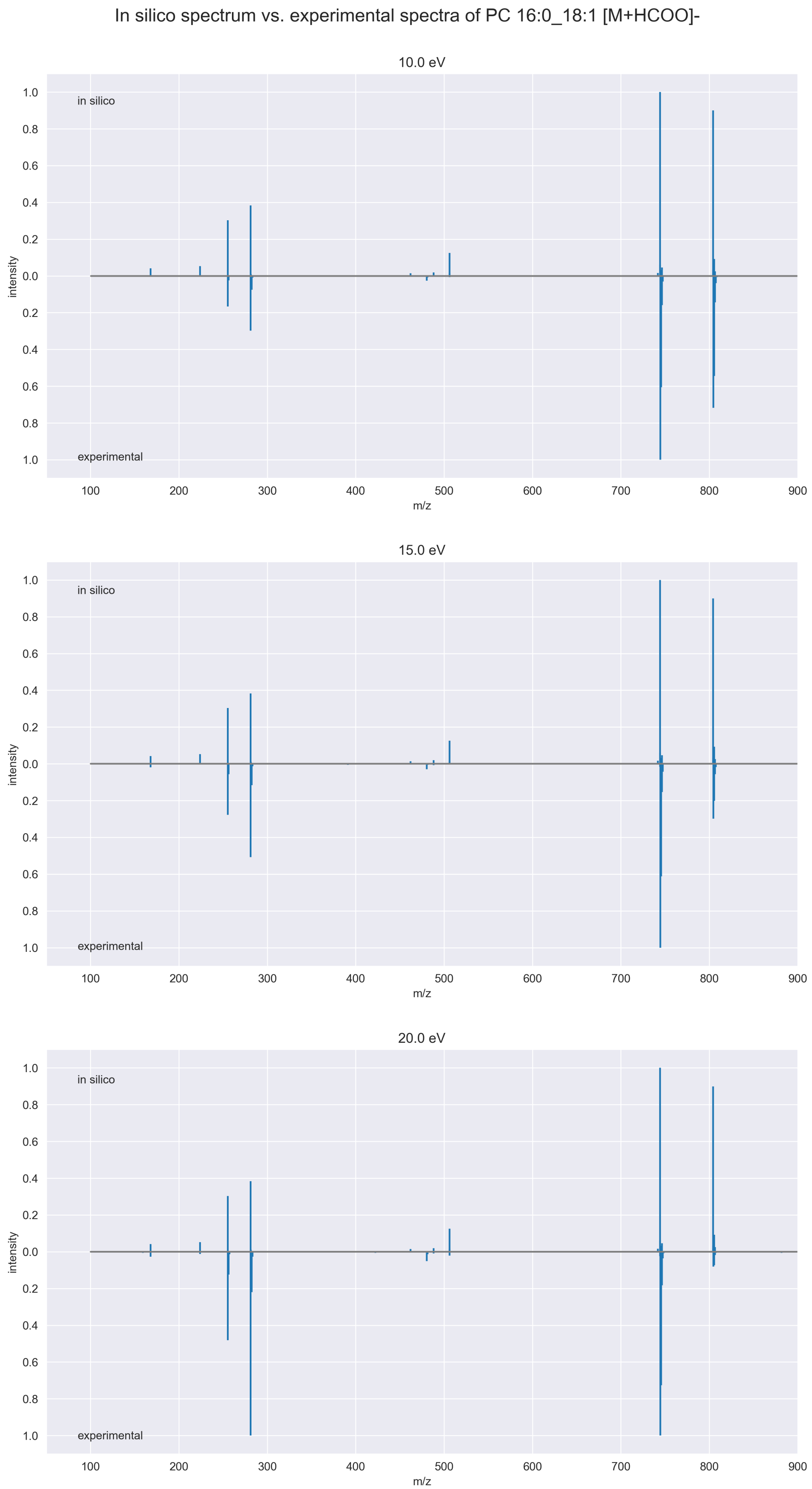

Figure S4.4: *Comparison between predicted in silico spectrum for PC 16:0_18:1 and experimental spectra at different collision energies of A) 10 eV, B) 15 eV and C) 20 eV.*

S4.1 Integrated gradients of mispredictions

Interesting information can be gained from using integrated gradients on a misprediction. One misprediction that caught our attention involved a spectrum of `TG 22:5_22:6_22:6 [M+NH4]+` from PNNL, which the model predicted as `TG 16:0_22:6_22:6 [M+NH4]+`, thereby substituting the first fatty acid `22:5` with `16:0`. Given that this substitution would imply a substantially different precursor mass, this case prompted us to examine whether the model sufficiently incorporated precursor mass information into its decision process. Looking at the integrated gradients (Supplement Figure S4.5) one can see that this prediction was mainly influenced by the peak at 313.2 m/z.

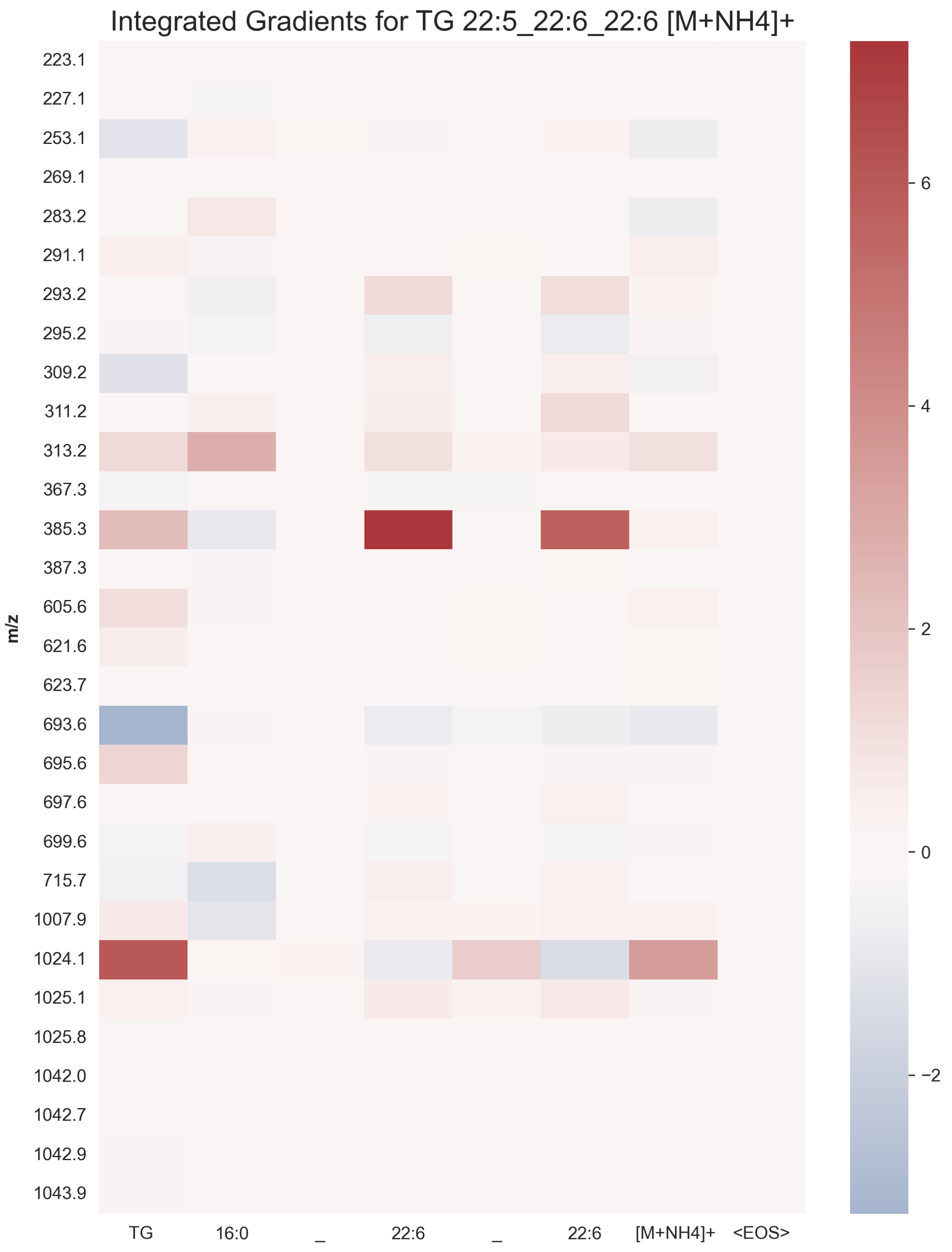

Figure S4.5: *Integrated gradients for misprediction of true label TG 22:5_22:6_22:6 as TG 16:0_22:6_22:6.*

When cross-referencing with the Alex[123] database, this peak corresponds precisely to the `22:5` fatty acid fragment. At first glance, it seems surprising that the model would choose this as an informative peak to predict `16:0`. However, when looking at the expected fragments for `TG 16:0_22:6_22:6 [M+NH4]+` in the Alex[123] database, the exact same peak

appears at 313.2 m/z for the fragment `FA 16:0(+C3H6O2)`. These peaks would be differentiable by the second decimal place, as they correspond to 313.25 m/z for `FA 22:5` and 313.27 m/z for `FA 16:0(+C3H6O2)`. However, LipiDetective currently only uses m/z values with a precision of two decimal points. A simple solution for this issue would then be to increase the number of decimal places for the m/z values fed into the model. Unfortunately, this notably decreased performance on the validation splits. One likely reason for this is the relatively small dataset size, resulting in the model's inability to adjust to the enormous increase of possible input features from 16,000 to 160,000 possible peaks. Additionally, when increasing the decimal place, each peak appears less frequently, and the learned peak embeddings are likely not quite as informative.

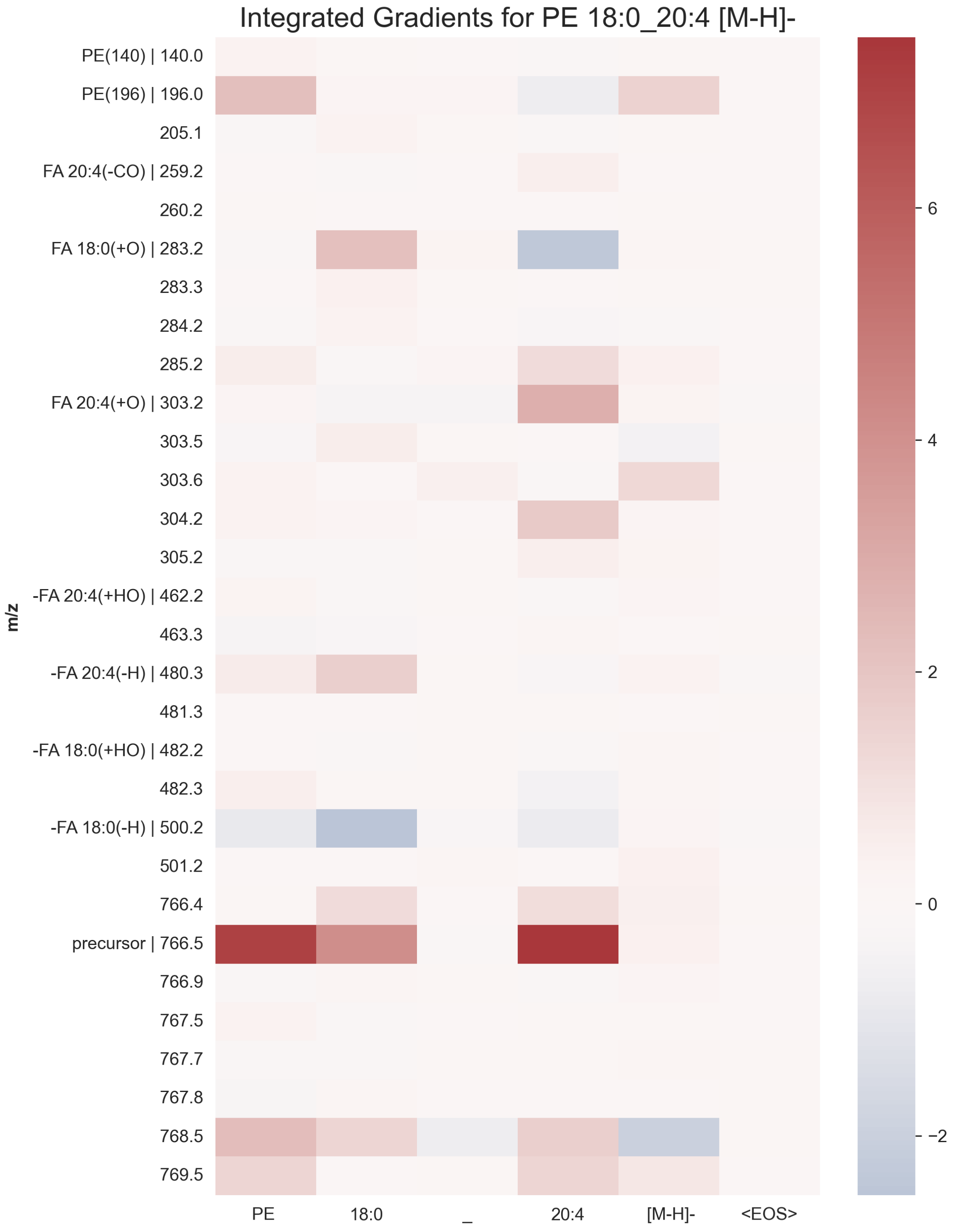

Figure S4.6: *Example of integrated gradients for PE 18:0_20:4 [M-H]-.*

It is also surprising that the model mispredicted the fatty acid position as it does seem to consider the precursor mass at 1024.1 m/z. However, this attribution appears to primarily influence the lipid class token, adduct and the structural tokens defining the overall composition, including the second underscore, which implicitly determines that a third fatty acid must

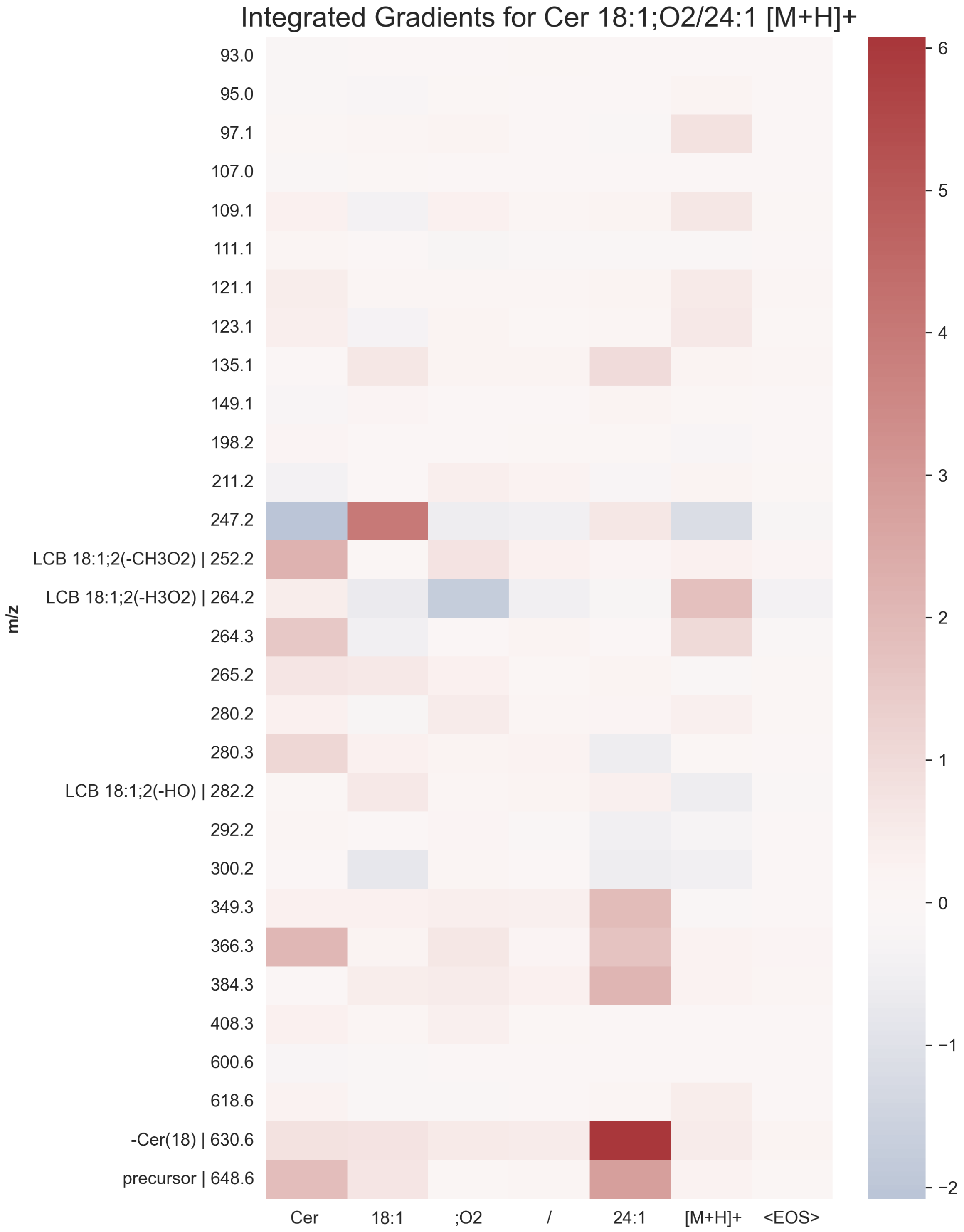

Figure S4.7: *Example of integrated gradients for Cer 18:1;O2/24:1 [M+H]+*

follow. In contrast, the precursor mass contributes comparatively little to the prediction of the individual fatty acid identities, suggesting that these are inferred predominantly from fragment evidence rather than from the global mass constraint. This is where the imbalance of the dataset is relevant again, as it leads to a bias towards the more commonly occurring fatty acids. For TGs in the training data, the fatty acid `16:0` occurs 12,794 times in the first position compared to `22:5`, which occurs only two times. When disregarding the position, `16:0` occurred 21,131 times compared to only 583 times for `22:5`. This means the model is much more likely to predict a more common fatty acid such as `16:0` if it can find reasonably fitting peaks. This could also be remedied to a certain extent by expanding the dataset to cover more uncommon fatty acids.

Additional integrated gradient visualizations for the most frequently occurring lipid species of the PE, Cer, and DG classes are shown in Supplementary Figures S9–S11.

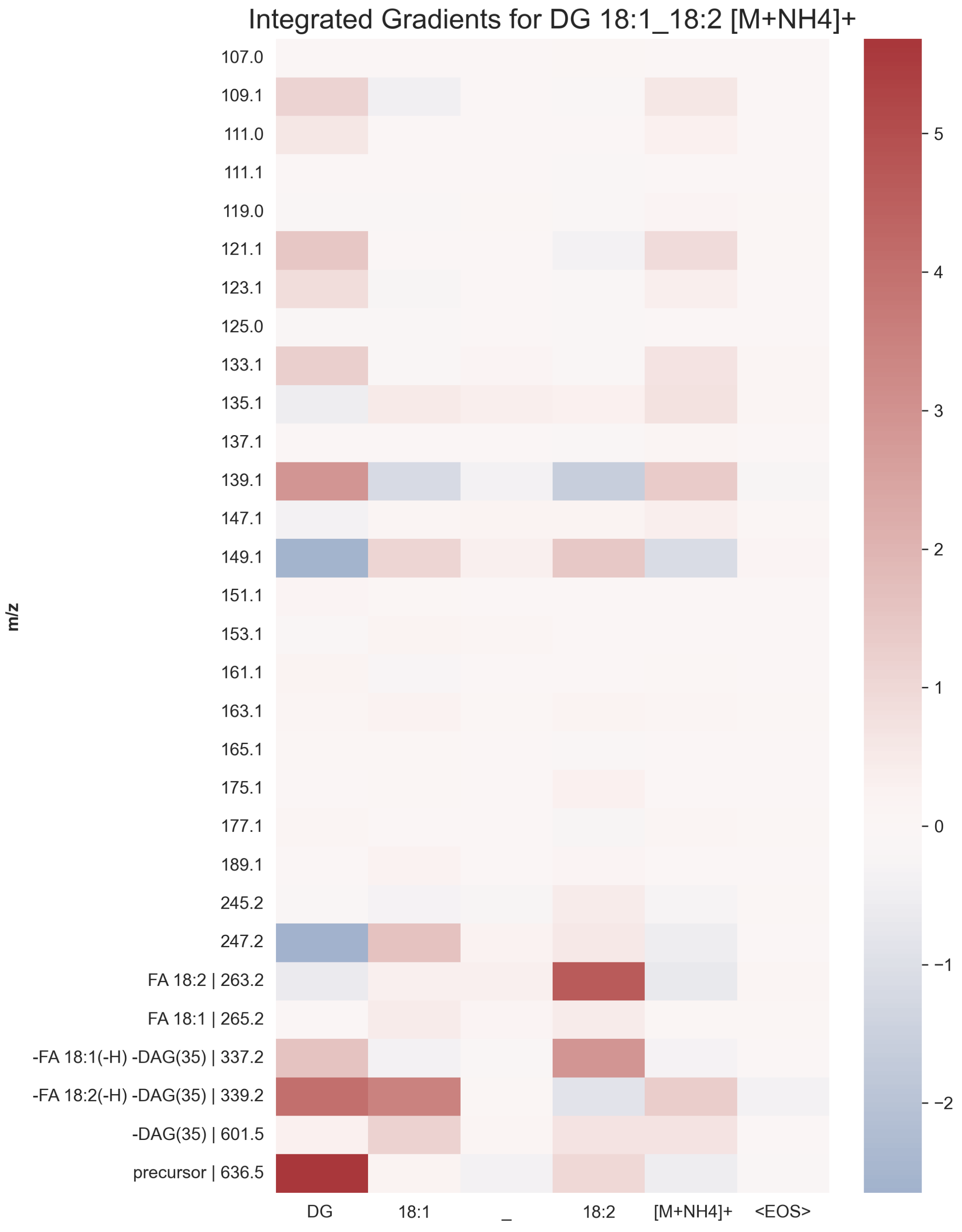

Figure S4.8: *Example of integrated gradients for DG 18:1_18:2 [M+NH4]+*

S4.2 Robustness

S4.2.1 Cross-Instrument Generalization

To evaluate instrument generalization, we performed leave-one-source-out experiments for 5 data sources. For each experiment, a model was trained on all data excluding one source and tested on the held-out source. To ensure a fair comparison across sources with different species coverage, we report accuracy on seen species only (species$\times$adduct pairs present in training data): Agilent (92.4%, n=9,382), Bruker (78.2%, n=24,412), Sciex (84.8%, n=43,024), IOBA-NHC (46.9%, n=128), HCE (56.5%, n=62). Across all sources, seen-species exact-match accuracy averaged 71.7 $\pm$ 19.3%. The highest accuracy was achieved on agilent (92.4%, 9,382 seen spectra), while the lowest was on IOBA-NHC (46.9%, 128 seen spectra).

The leave-one-source-out results demonstrate that LipiDetective can generalize across instrument platforms, though the degree of transfer varies substantially with the characteristics of the held-out source. Notably, the Agilent, Bruker, and Sciex sources consist exclusively of phospholipid standards, covering only 5 lipid classes and approximately 54 species each, whereas the IOBA and HCE sources span a broader range of lipid classes (9 and 13, respectively) and species (142 and 100). The high accuracy on Agilent data (92.4%) is particularly notable because this represents a true zero-shot instrument transfer-the model had never encountered Agilent spectra during training-and suggests that the fragmentation patterns of phospholipid standards are sufficiently conserved across instruments to support reliable identification. The strong performance on Sciex (84.8%) and Bruker (78.2%) is especially encouraging given that these two sources together contribute approximately 67,000 spectra to the training set; holding out either one removes a substantial fraction of the training data, yet the model still achieves high accuracy on the remaining instrument's spectra. In contrast, the lower accuracy on the IOBA (46.9%) and HCE (56.5%) sources reflects a combination of their very small sample sizes (128 and 62 seen spectra, respectively) and their greater lipid diversity: these sources include lipid classes and species that are underrepresented in the remaining training data, making the identification task inherently harder. With so few spectra, individual misclassifications also have an outsized effect on the reported accuracy.

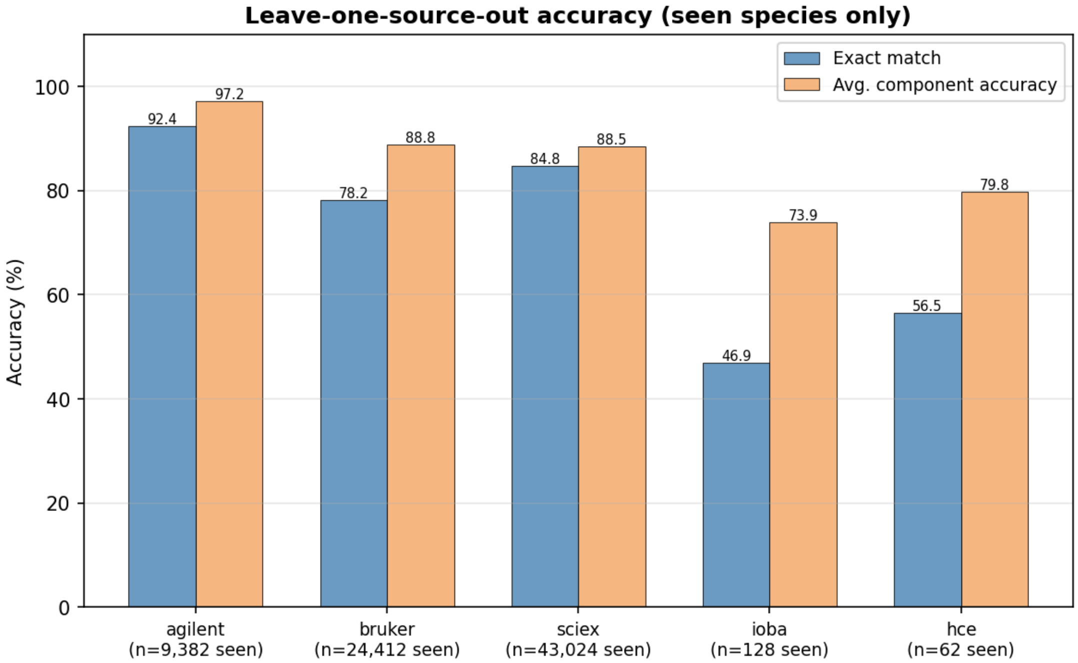

Figure S4.9: *Leave-one-source-out accuracy on seen species. For each of five data sources, a model was trained on all remaining data and evaluated on the held-out source, restricted to species$\times$adduct pairs present in the training set.* ***Exact match*** *(blue) requires the full predicted lipid nomenclature to match the ground truth;* ***Average component accuracy*** *(orange) reports the mean per-component accuracy across lipid name components (headgroup, fatty acyl chains, adduct), giving partial credit for partially correct predictions.*

S4.2.2 Collision Energy Impact

To assess the impact of collision energy on prediction accuracy, we used the model trained on all non-Agilent data from the leave-one-instrument-out experiment and evaluated the held-out Agilent spectra stratified by their collision energies ranging from 10.0 to 50.0 eV in 2.5 eV increments. Accuracy exhibited a characteristic dependence on collision energy, peaking in the 20-35 eV range (94.2% mean exact accuracy) and declining at both extremes: low collision energies ($\leq$15 eV, 91.6%) likely produce insufficient fragmentation for unambiguous identification, while high collision energies ($\geq$42.5 eV, 88.9%) lead to over-fragmentation that degrades diagnostic fragment patterns. The highest per-bin accuracy of 95.6% was observed at 32.5 eV, while the lowest of 85.7% occurred at 50.0 eV. Mean confidence scores correlated with accuracy, decreasing from 0.98 at mid-range energies to 0.85 at 50.0 eV, indicating that the model's uncertainty estimates reflect prediction difficulty.

These results reveal a characteristic inverted-U relationship between collision energy and identification accuracy, consistent with the known physics of collision-induced dissociation. The sweet spot of 20-35 eV coincides with the energy range most commonly used in lipidomics workflows, suggesting that LipiDetective is well-calibrated for typical experimental conditions. The strong correlation between model confidence and accuracy further suggests that the softmax-derived confidence scores can serve as a practical quality filter: spectra acquired at suboptimal collision energies, where accuracy drops, are flagged by correspondingly lower confidence scores, enabling users to set a confidence threshold that preferentially retains reliable identifications.

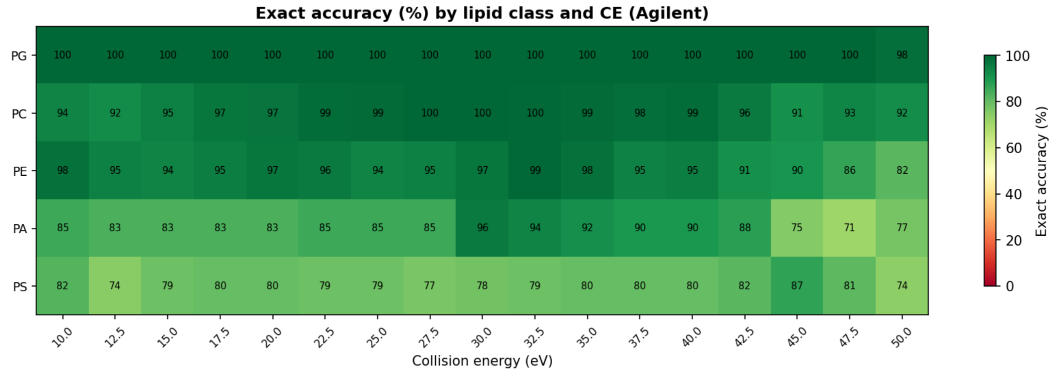

Figure S4.10: *Exact-match accuracy (%) by lipid class and collision energy on held-out Agilent phospholipid standards. Each cell reports the percentage of spectra correctly identified at a given collision energy (columns, 10.0-50.0 eV in 2.5 eV steps) for each lipid class (rows).*

S4.3 Per-class Metrics and Confusion Patterns on the Internal Validation Set

To provide a detailed per-class characterization of model performance on the internal validation set, we report precision, recall (sensitivity), F1 score, false discovery rate (FDR = 1 - Precision), and specificity for each lipid class. Metrics are computed from the 100-spectra count split (3,537 validation spectra across 100 held-out lipid species spanning 14 classes) at the final training epoch. The class-level confusion matrix is constructed by parsing the lipid class from each predicted and true species name, ensuring that all predictions-including empty outputs (`<EOS>`) and malformed sequences-are accounted for.

S4.3.1 Per-class Precision, Recall, F1, FDR, and Specificity

Table S4.4 summarizes per-class metrics sorted by F1 score. CAR achieves perfect classification (F1 = 100%), reflecting its distinctive acylcarnitine fragmentation pattern. TG (F1 = 97.1%), PC (86.2%), and PE (86.2%) also achieve strong performance, consistent with their large representation in the training data and characteristic fragmentation signatures. DG (83.3%) and SM (80.3%) show high precision (>91%) but lower recall, indicating that the model is conservative in predicting these classes, when it does predict them, it is usually correct, but it misses some instances.

Among the weaker classes, PG (F1 = 59.2%) and PS (49.9%) show roughly balanced precision and recall, suggesting systematic confusion between structurally similar glycerophospholipid classes. The lyso-lipid classes LPG (F1 = 3.3%) and LPS (15.7%) perform poorly despite high precision for LPS (100%), because the model rarely predicts these classes (recall <10%), likely due to their limited training representation relative to their full-chain counterparts.

FDR is below 10% for 8 of 14 classes, indicating that most positive predictions are reliable. The highest FDR values are observed for LPG (86.7%) and PS (51.2%), where the model frequently confuses these classes with structurally related lipids. Specificity exceeds 95% for all classes, reflecting the multi-class nature of the task: the model correctly identifies non-members of each class in the vast majority of cases.

S4.3.2 Class-level Confusion Matrix

Supplementary Figure S4.11 presents the row-normalized class-level confusion matrix, showing the percentage of spectra from each true class (rows) predicted as each class (columns). The matrix includes two special columns: `<EOS>` (the model produced an empty prediction, i.e., only the end-of-sequence token) and `Other` (the model predicted a malformed or unrecognized sequence).

The diagonal is strong for CAR (100%), TG (100%), PC (90%), and PE (95%), confirming that these classes are reliably identified. The most prominent off-diagonal confusion occurs between structurally related glycerophospholipid classes: PS spectra are frequently misclassified as PE (35%) and PC (7%), consistent with the shared glycerophosphate backbone and similar fragmentation patterns in negative ionization mode. PG shows confusion with PE (19%) and PS (9%), reflecting the structural similarity of these anionic phospholipids. Cer spectra are occasionally confused with SM (10%) and GalCer (5%), which share the ceramide backbone but differ in headgroup.

The lyso-lipid classes show a distinctive pattern: LPS is predominantly misclassified as PS (87%), LPG as PG (44%) and PE (36%), and LPI as PI (64%). This systematic confusion with the corresponding full-chain class suggests that the model

recognizes the headgroup fragmentation but struggles to distinguish single-chain from dual-chain species, likely because lyso-lipids produce a subset of the fragments generated by their diacyl counterparts.

Table S4.4: *Per-class precision, recall (sensitivity), F1 score, false discovery rate (FDR), and specificity on the internal validation set (100-spectra count split, 3,537 spectra). Metrics are computed from the class-level confusion matrix at the final training epoch. Classes are sorted by F1 score in descending order.*

Class	Support	Precision	Recall	F1	FDR	Specificity
CAR	195	100.0%	100.0%	100.0%	0.0%	100.0%
TG	686	94.6%	99.7%	97.1%	5.4%	98.6%
PC	496	83.1%	89.5%	86.2%	16.9%	97.0%
PE	483	78.5%	95.4%	86.2%	21.5%	95.9%
DG	296	92.2%	76.0%	83.3%	7.8%	99.4%
SM	296	91.4%	71.6%	80.3%	8.6%	99.4%
Cer	189	74.9%	82.0%	78.3%	25.1%	98.4%
GalCer	191	94.3%	52.4%	67.3%	5.7%	99.8%
PG	107	59.4%	58.9%	59.2%	40.6%	98.7%
PS	194	48.8%	51.0%	49.9%	51.2%	96.9%
LPC	95	75.6%	32.6%	45.6%	24.4%	99.7%
LPI	95	100.0%	21.1%	34.8%	0.0%	100.0%
LPS	106	100.0%	8.5%	15.7%	0.0%	100.0%
LPG	108	13.3%	1.9%	3.3%	86.7%	99.6%

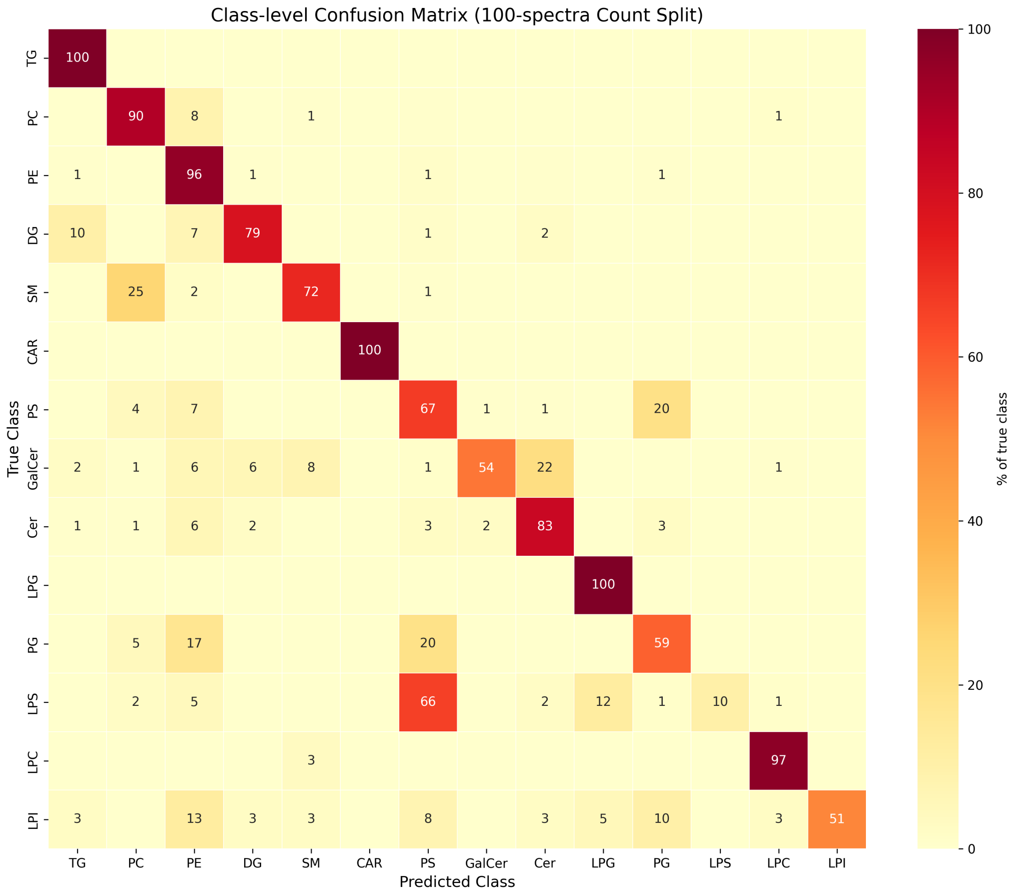

Figure S4.11: *Row-normalized class-level confusion matrix on the internal validation set (100-spectra count split, 3,537 spectra, final training epoch). Values indicate the percentage of spectra from each true class (rows) predicted as each class (columns). The `<EOS>` column indicates empty predictions; `Other` indicates malformed outputs. Cells with values below 0.5% are left blank for readability. Classes are sorted by support (number of validation spectra) in descending order.*
