# Supplementary material for: LipiDetective: a deep learning model for the identification of molecular lipid species in tandem mass spectra
Source: Brief Bioinform. 2026 Jul 27;27(4):bbag378. doi: 10.1093/bib/bbag378 (PMC13403187; doi:10.1093/bib/bbag378)
Supplement: Supplementary-material_bbag378 [file supplementary-material_bbag378.zip › LipiDetective_Supplement_5_bbag378.pdf]

Supplement 5: MassBank External Test Set Evaluation

To assess generalization beyond our curated training data, we evaluate LipiDetective on 1,549 spectra drawn from MassBank [38], an independent public repository of experimentally acquired tandem mass spectra. This test set spans 12 lipid classes, both positive and negative ionization modes, and multiple instrument platforms. The presented results cover two conditions:

- **Raw**: all 1,549 MassBank spectra, regardless of instrument type.
- **Quality-filtered**: 393 spectra acquired on beam-type CID instruments only (QTOF, TOF, QFT), excluding ion trap-based fragmentation platforms (LC-ESI-ITFT, LC-ESI-IT, LC-ESI-QQ).

The quality filter is motivated by the fact that ion trap CID instruments apply resonance excitation that subjects ions to the one-third rule, suppressing low-mass fragments that might be critical for lipid class identification. Because the training data is dominated by beam-type CID spectra, the model has learned to rely on fragmentation patterns absent from ion trap spectra.

S5.1 Test Set Class Distribution

Figure S5.1 shows the lipid class distribution for both conditions. The raw test set is dominated by PE (22.0%) and PC (18.9%), followed by Cer (8.8%), LPC (8.4%), and PI (6.8%). The quality filter substantially shifts the class balance: Cer rises from 8.8% to 22.4%, TG from 4.6% to 18.8%, while PE drops from 22.0% to 2.3%. This shift occurs because certain classes are disproportionately represented on specific instrument platforms-notably, PE and PC are heavily represented on the Thermo LTQ Orbitrap (ion trap CID), which is excluded from the quality-filtered set.

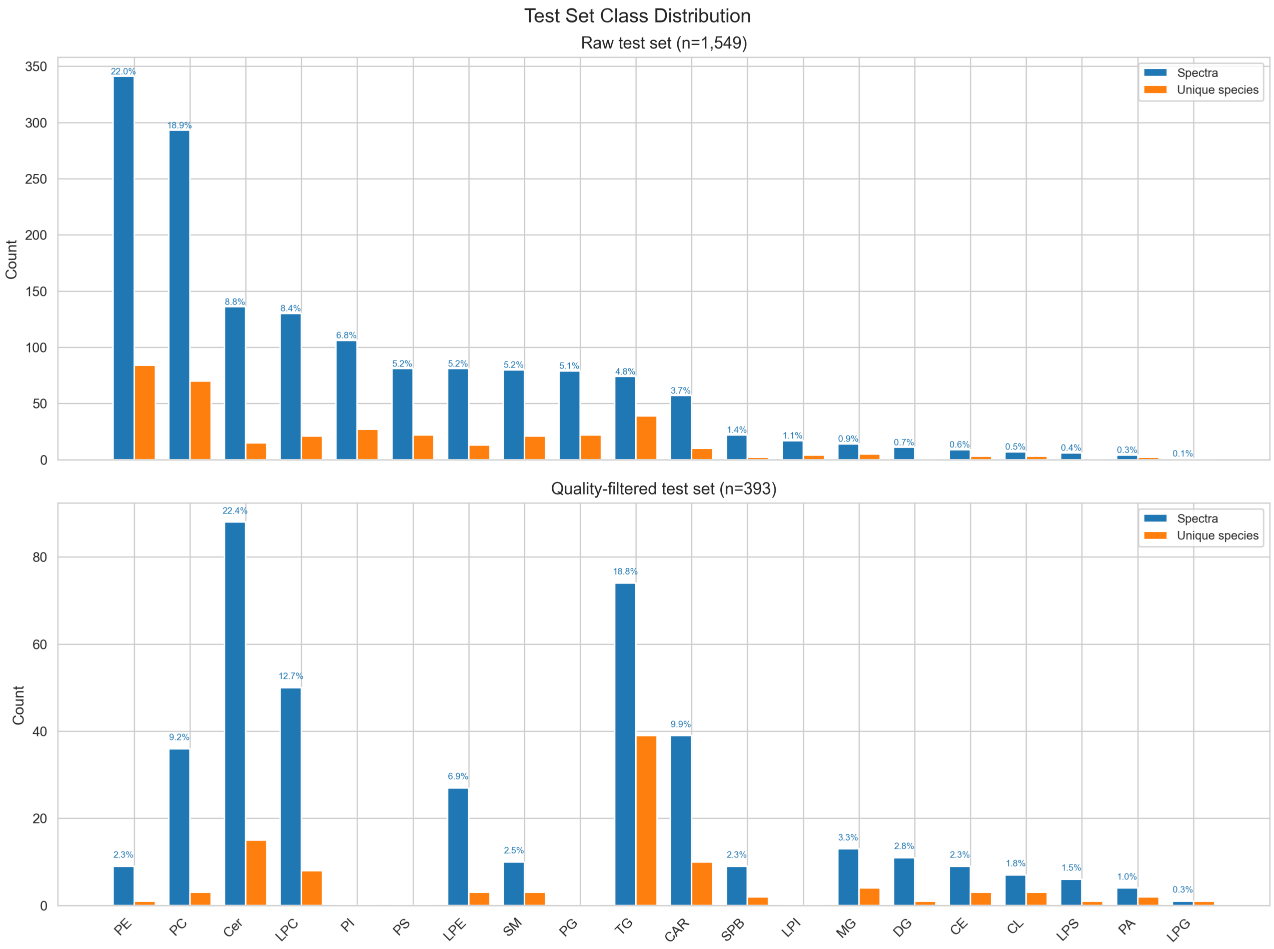

Figure S5.1: Lipid class distribution in the MassBank external test set. Top: raw test set (1,549 spectra across 12 classes). Bottom: quality-filtered test set (393 spectra, beam-type CID instruments only). Blue bars indicate the number of spectra; orange bars indicate the number of unique molecular species. Percentages above bars refer to the proportion of total spectra.

S5.2 Performance by Mass Spectrometer

Figure S5.2 summarizes performance by instrument platform across all 1,549 spectra. The top panel shows accuracy metrics: beam-type CID instruments (QTOF, TOF) consistently outperform ion trap platforms. The Sciex TripleTOF 6600 achieves the highest exact-match accuracy (∼70%), while the Thermo LTQ Orbitrap-which contributes 1,075 of the

1,549 spectra-achieves only ∼8%. The bottom panel shows macro-averaged class-level precision, recall, and F1 scores per instrument. Beam-type CID instruments achieve high macro-averaged F1 scores (Agilent 6530, Sciex TripleTOF 6600, Bruker maXis plus), while the Thermo LTQ Orbitrap achieves only ∼30% macro-averaged F1. However, these per-instrument F1 scores should be interpreted cautiously: lipid class composition varies substantially across instruments (e.g., the Sciex TripleTOF 6600 contributes only 1 class, making high F1 trivial, whereas the LTQ Orbitrap spans 9 classes including structurally similar phospholipids). The performance gap therefore reflects a combination of the ion trap CID mechanism, which suppresses low-mass headgroup-diagnostic fragments via the one-third rule, and differences in class composition and complexity across instrument platforms.

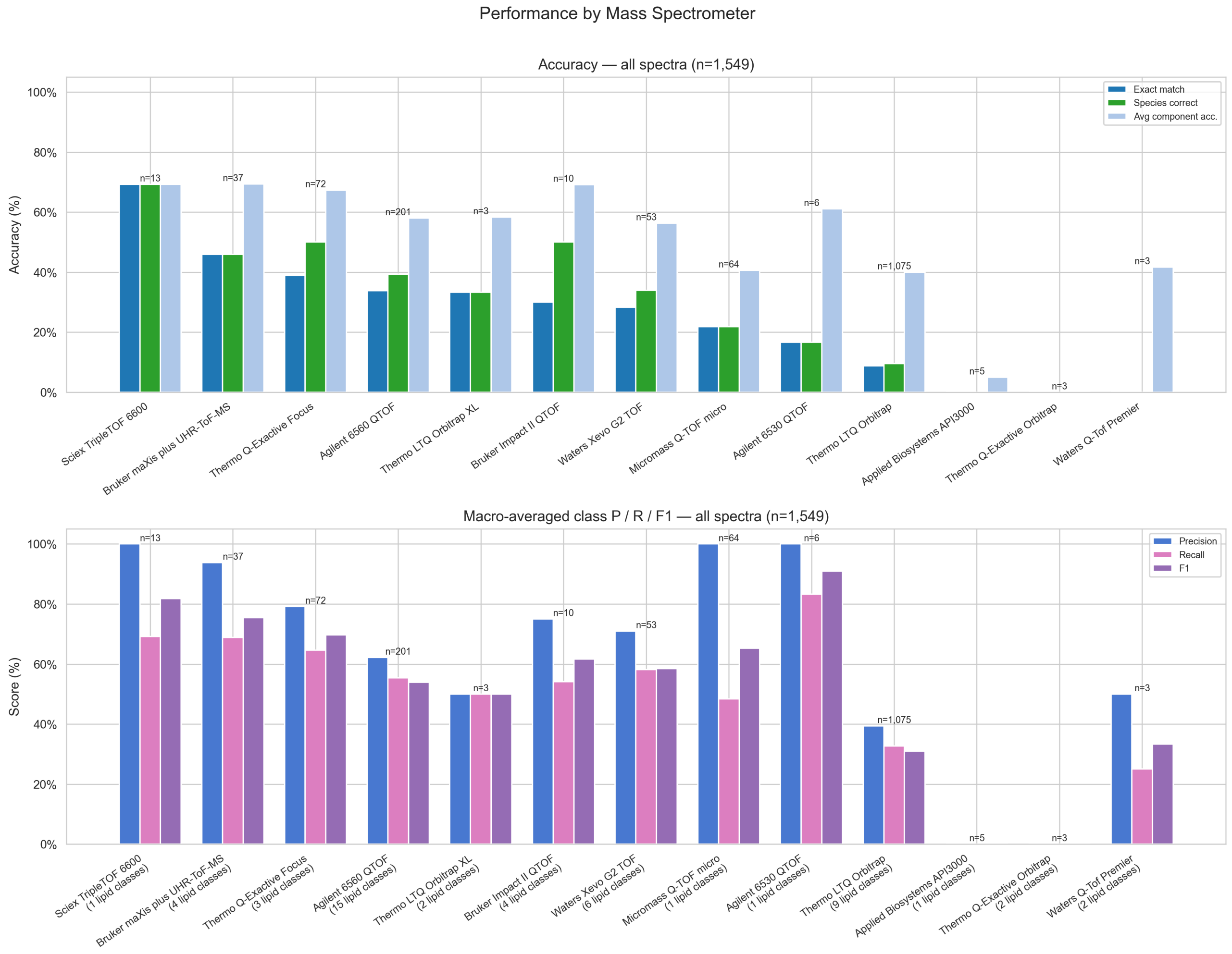

Figure S5.2: Performance by mass spectrometer on the MassBank test set (all 1,549 spectra). Top: accuracy metrics per instrument-exact match (full nomenclature correct), species correct (correct lipid species ignoring adduct), and average component accuracy. Bottom: macro-averaged lipid class-level precision, recall, and F1 score per instrument, computed across all lipid classes present on each instrument. Instruments are sorted by exact-match accuracy in the top panel with the bottom panel matching the sorting for comparison purpose). Only instruments with ≥3 spectra are shown; sample sizes are annotated above each bar group.

S5.3 Per-class Precision, Recall, F1 Score, and Species Accuracy

To address imbalanced class representation, we report per-class precision, recall, F1 scores, and species-level accuracy for all lipid classes with ≥10 spectra in the seen subset (exact species-adduct combination observed during training). Figure S5.3 shows that quality filtering substantially improves performance across all classes. In the raw test set, CAR achieves the highest F1 (94%), followed by TG (64%) and Cer (62%). After quality filtering, LPE and CAR both reach 100% F1, and CAR, LPC, and Cer all exceed 80%. Species-level accuracy, which requires the model to predict the correct molecular species including sidechain composition, is consistently lower than class-level F1, particularly for structurally diverse classes such as TG (24% species accuracy vs. 68% F1 in the filtered set).

S5.4 Lipid Class Confusion Matrix

Supplementary Figure S5.4 presents row-normalized confusion matrices for the seen subset. In the raw test set, the diagonal is strong for CAR (100%), Cer (95%), TG (95%), and LPE (83%), but substantial off-diagonal mass appears for PC, PE, and PG-classes whose spectra share similar phospholipid fragmentation signatures. PE spectra are frequently misclassified as PE itself retains only 41% on-diagonal, with confusion spread across PC (3%), PG (16%), and PI (4%). PG shows

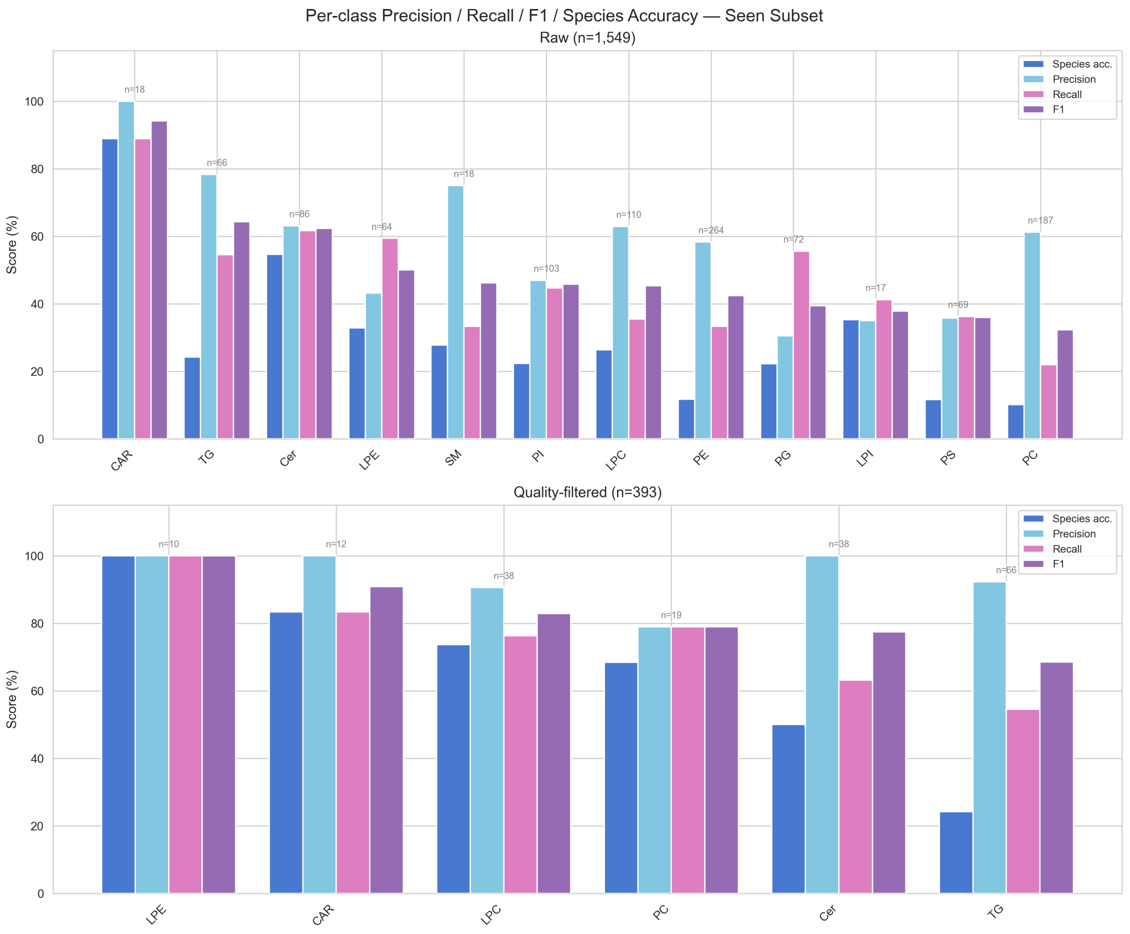

Figure S5.3: *Per-class precision, recall, F1 score, and species accuracy on the MassBank test set (seen subset only, classes with ≥10 spectra). Top: raw test set. Bottom: quality-filtered test set. Classes are sorted by F1 score in descending order. Numbers above bars indicate the number of spectra per class.*

a similar pattern, with 63% on-diagonal and 21% confused with PE. Quality filtering dramatically reduces inter-class confusion: CAR, LPE, and TG all reach 100% on-diagonal accuracy, Cer reaches 89%, LPC 88%, and PC 83%. The improvement for PC and LPC is particularly notable, as the characteristic phosphocholine headgroup fragment at 184.07 Da (for $[M+H]^+$) becomes detectable with beam-type CID but is suppressed below the one-third rule cutoff in ion trap spectra.

S5.5 Accuracy by Training Overlap

We stratify spectra by whether the exact species-adduct combination was observed during training (*seen*; n=1,089 raw, n=213 filtered), the species was observed but with a different adduct (*novel adduct*; n=227 raw, n=104 filtered), or the species was entirely absent from the training set (*unseen*; n=233 raw, n=76 filtered). Figure S5.5 shows that quality filtering nearly triples exact-match accuracy for the seen subset (from 21.6% to 54.5%) and roughly doubles average component-level accuracy (from 50.9% to 71.0%). For novel-adduct spectra, exact-match accuracy increases from 4.8% to 9.6%, while average component accuracy rises from 29.8% to 43.8%. Unseen spectra remain challenging in both conditions (2.1% and 1.3% exact match, respectively), though their average component accuracy (34.1% raw, 32.0% filtered) suggests the model partially identifies structural features even for species outside its training distribution.

S5.6 Confidence Calibration

To evaluate the reliability of the model's confidence scores, we construct reliability diagrams that compare binned predicted confidence to actual exact-match accuracy (Supplementary Figure S5.6). The histograms below each reliability curve show the distribution of spectra across confidence bins. In the raw test set, the model is substantially overconfident: confidence scores in the 60-80% range correspond to actual accuracy below 25%, yielding an expected calibration error (ECE) of 37.1%. Confidence scores are spread relatively uniformly across all bins, indicating that the model assigns high confidence even to spectra from incompatible instruments. After quality filtering, calibration improves considerably-high-confidence predictions (>80%) achieve accuracy above 60%, and the ECE drops to 18.2%. The confidence distribution also shifts rightward, with the majority of spectra concentrated in the 90-100% bin. This suggests that the confidence score can serve as a useful filter for downstream analyses when instrument compatibility is ensured, but should not be trusted uncritically for ion trap spectra.

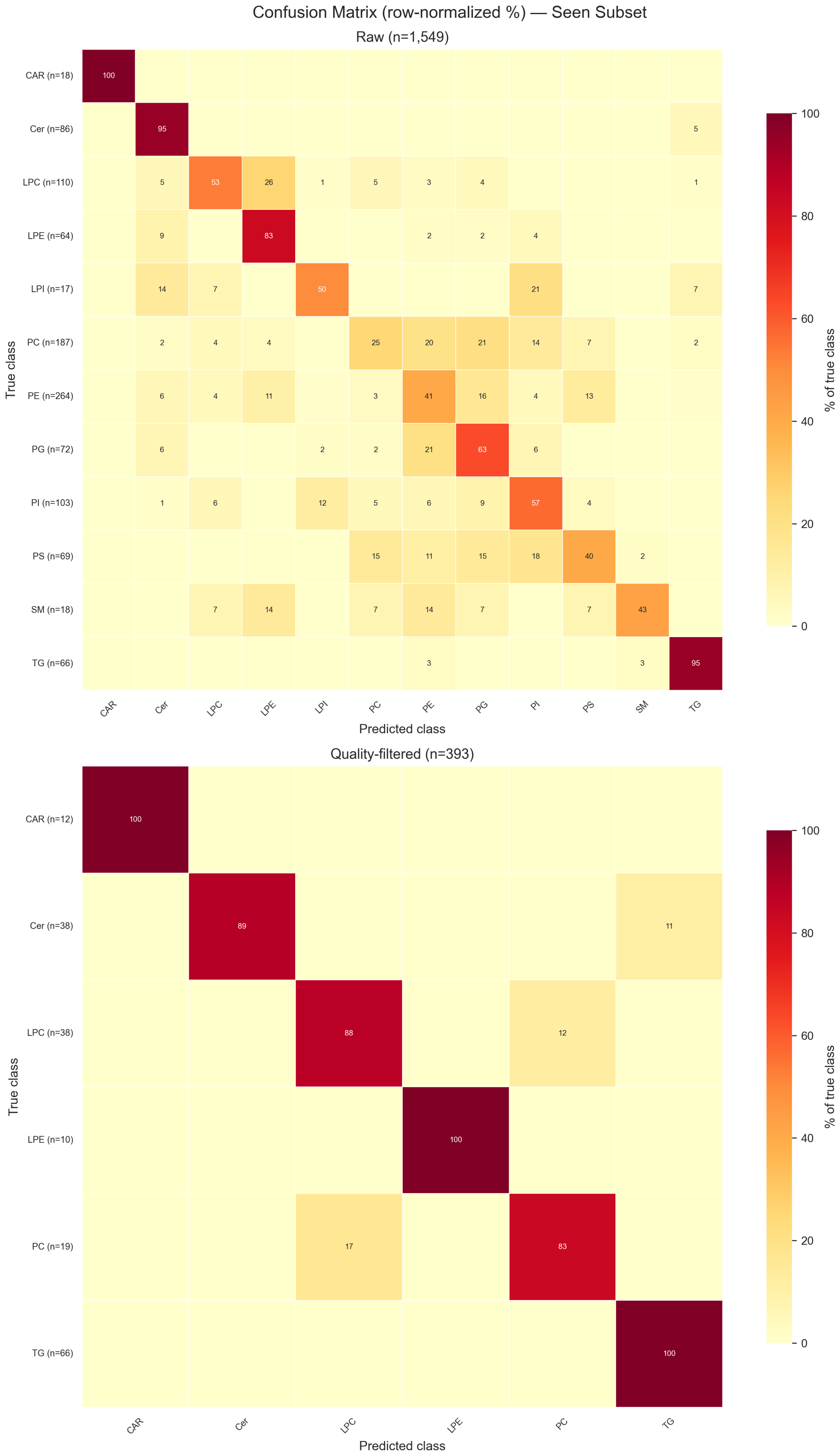

Figure S5.4: *Row-normalized confusion matrices on the MassBank test set (seen subset, classes with ≥10 spectra). Values indicate the percentage of spectra from each true class (rows) predicted as each class (columns). Top: raw test set. Bottom: quality-filtered test set. Cells with values below 0.5% are left blank for readability.*

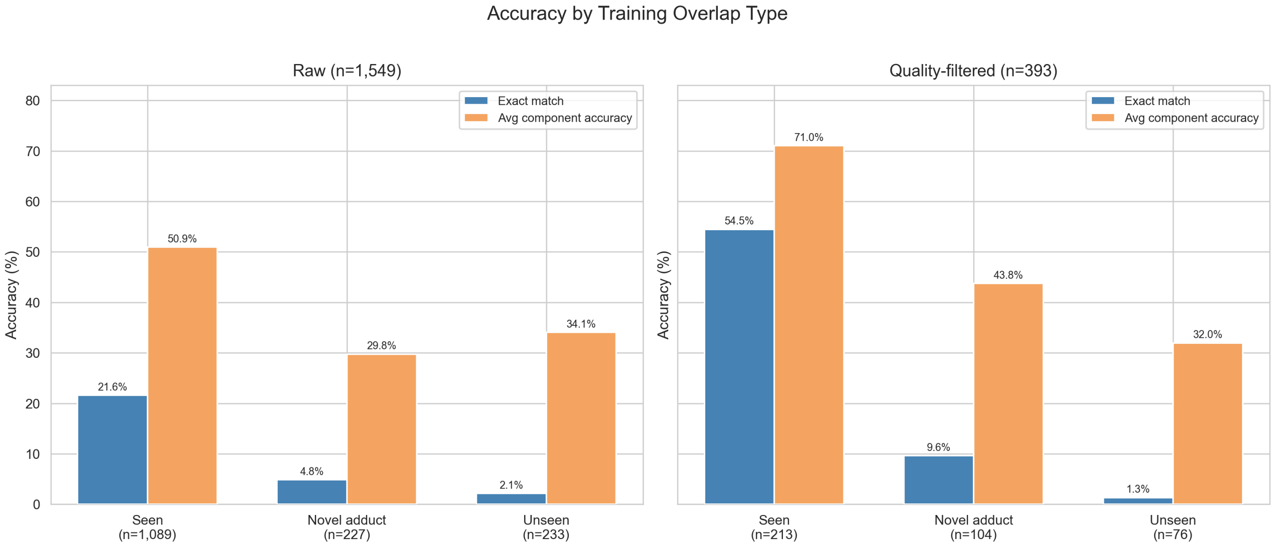

Figure S5.5: *Accuracy stratified by training overlap on the MassBank test set. Left: raw test set. Right: quality-filtered test set. "Seen" indicates the exact species-adduct pair was in the training set; "Novel adduct" indicates the species was seen but with a different adduct; "Unseen" indicates the species was absent from training. Sample sizes are indicated below each group label.*

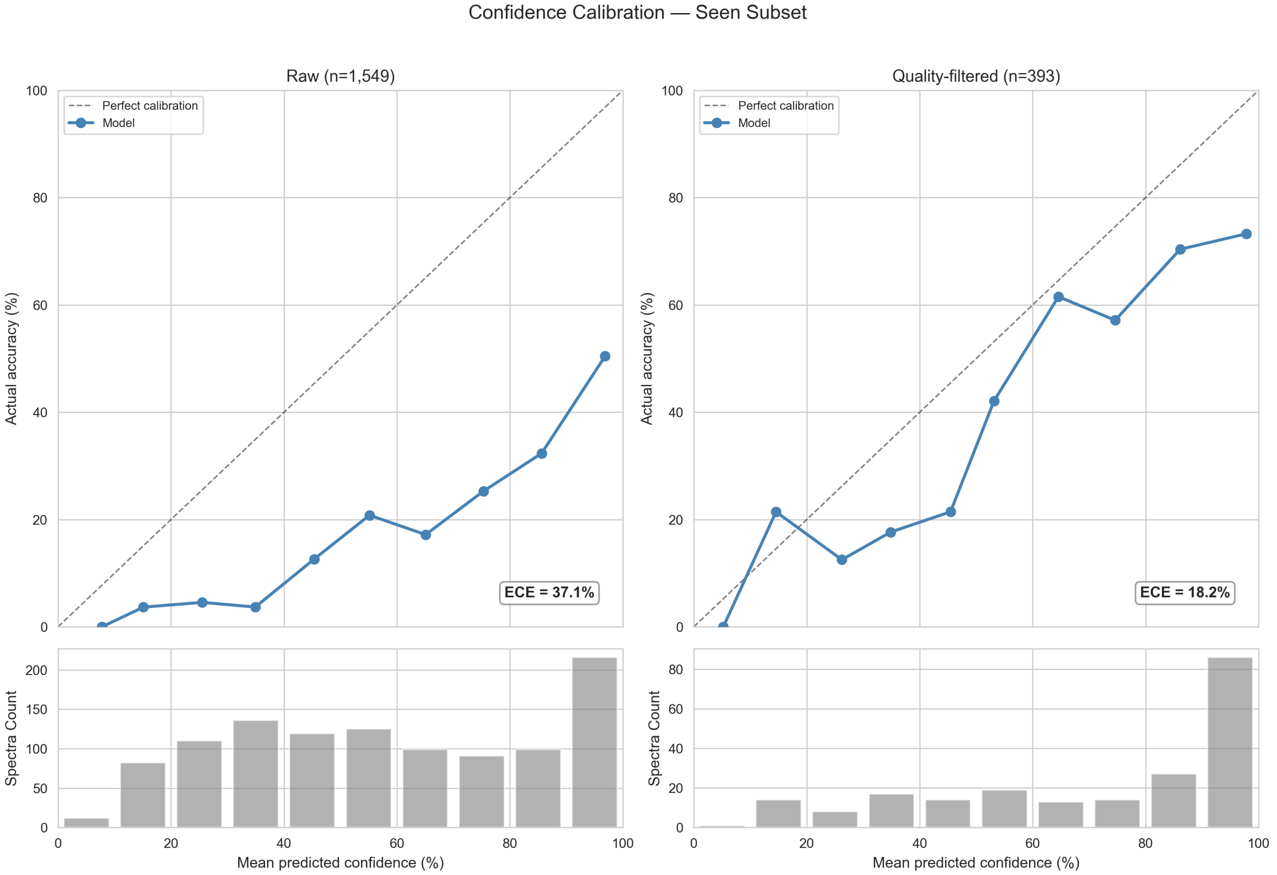

Figure S5.6: *Confidence calibration on the MassBank test set (seen subset). Top row: reliability diagrams comparing binned predicted confidence (x-axis) to actual exact-match accuracy (y-axis); the dashed line indicates perfect calibration. The expected calibration error (ECE) is annotated in each panel. Bottom row: histograms showing the number of spectra per confidence bin. Left: raw test set. Right: quality-filtered test set.*

S5.7 Accuracy by Chain Length, Total Carbons, and Unsaturation

To assess whether prediction difficulty varies with lipid structural complexity, we stratify accuracy by three structural features (Supplementary Figure S5.7): individual sidechain carbon count (each spectrum contributes one observation per acyl chain), total carbon count summed across all chains, and total number of double bonds. For individual chain lengths, the most common biological fatty acids (16 and 18 carbons) show the highest accuracy in the quality-filtered set, with

exact-match accuracy reaching ∼60% and average component accuracy exceeding 70%. Shorter chains (≤12 carbons, typical of CAR species) and longer chains (≥22 carbons, typical of sphingolipids) show more variable performance. For total carbon count, accuracy tends to decrease for species with >40 total carbons, consistent with the combinatorial increase in possible sidechain compositions for multi-chain lipids. For unsaturation, saturated and mono-unsaturated species (0-1 double bonds) are generally predicted more accurately than polyunsaturated species, reflecting the greater structural ambiguity when multiple double bonds can be distributed across chains in different configurations.

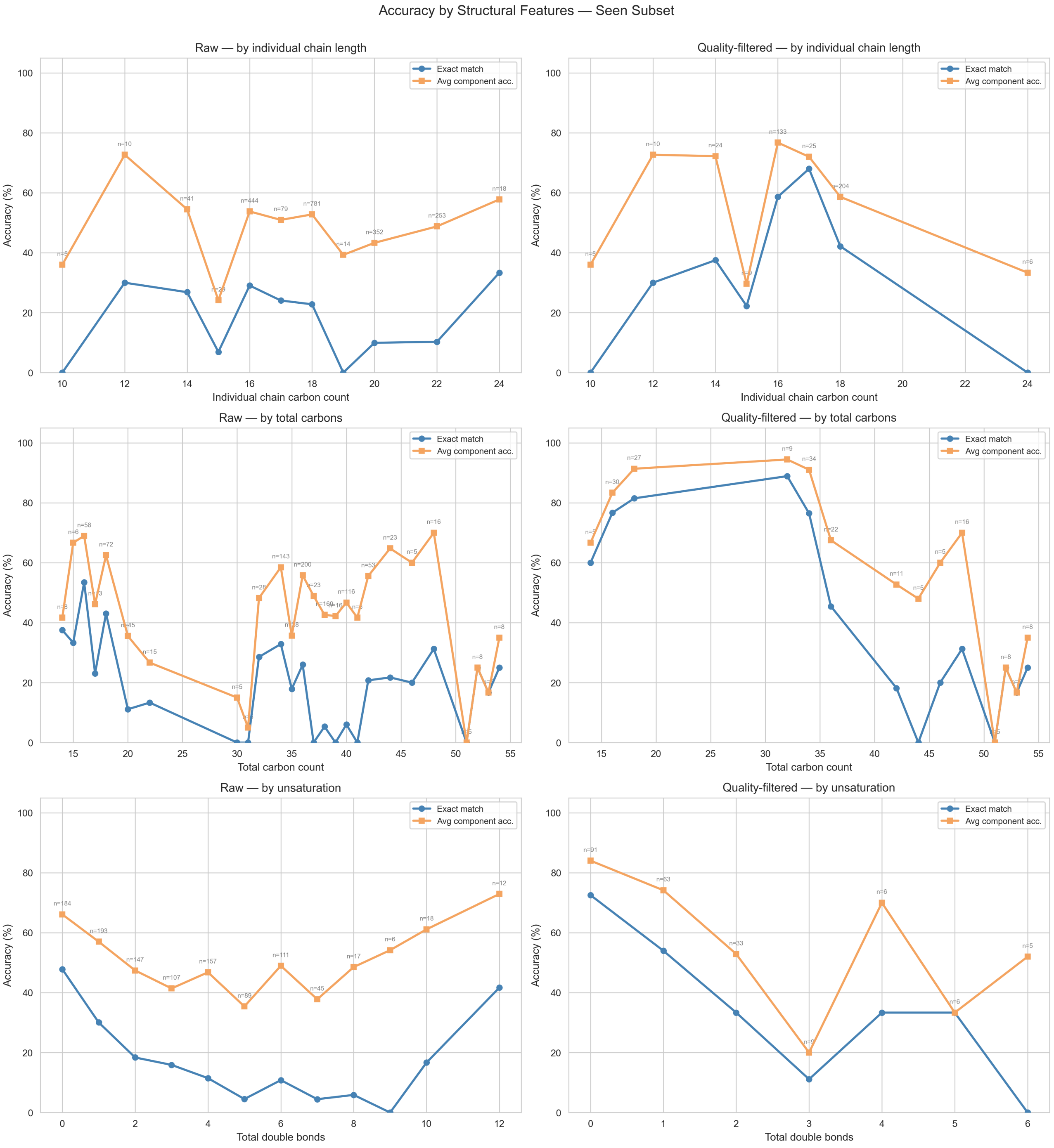

Figure S5.7: Accuracy stratified by structural features on the MassBank test set (seen subset). Left column: raw test set; right column: quality-filtered test set. Top row: accuracy by individual sidechain carbon count (each spectrum contributes one data point per acyl chain). Middle row: accuracy by total carbon count across all chains. Bottom row: accuracy by total number of double bonds. Only bins with ≥5 spectra are shown; sample sizes are annotated above each data point.

S5.8 Accuracy by Adduct Type

We stratify accuracy by the adduct ion observed in each spectrum (Supplementary Figure S5.8). For the seen subset, $[M+H]^+$ achieves the highest exact-match accuracy in both conditions (61% raw, 63% filtered), consistent with the rich fragmentation patterns produced by protonated molecular ions. $[M-H]^-$ shows lower exact-match accuracy (19% raw, 55% filtered) despite being the most abundant adduct (n=620 seen), likely because negative-mode spectra produce fewer

class-diagnostic fragments for some lipid classes. [M+OAc]$^-$ performs poorly in the raw set (3% exact match) but improves to 27% after quality filtering-these spectra are heavily represented on ion trap instruments. When all spectra are considered (including novel-adduct and unseen species), additional adduct types become visible: [M+HCOO]$^-$ (formate, n=15) achieves $\sim$57% average component accuracy despite being entirely absent from the training set, suggesting the model can partially generalize to structurally similar adducts. [M+Na]$^+$ and [M+Cl]$^-$, also novel adducts, show substantially lower performance (4% and 0% exact match, respectively), as sodium and chloride adducts produce fundamentally different fragmentation patterns.

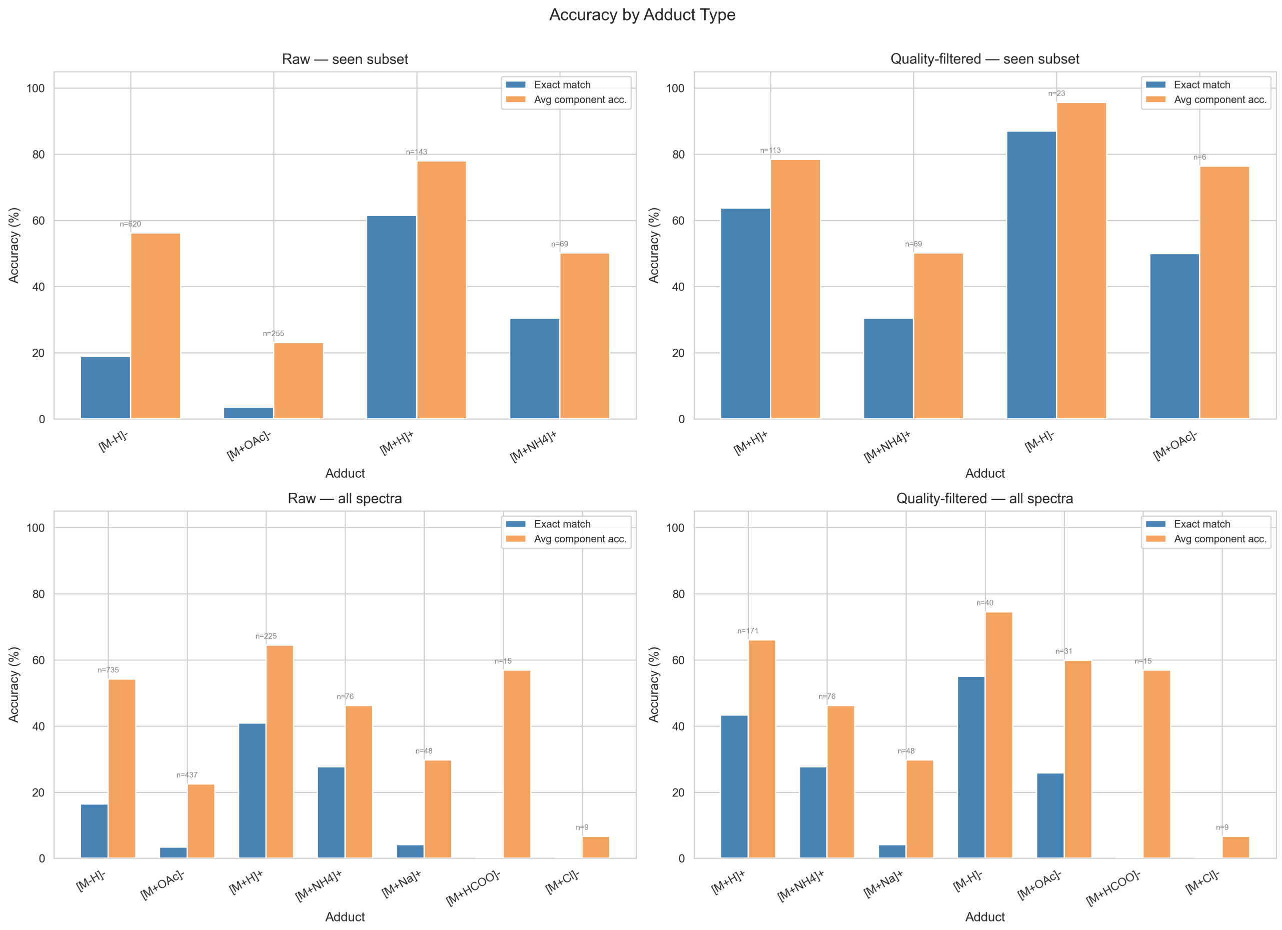

Figure S5.8: *Accuracy stratified by adduct type on the MassBank test set. Left column: raw test set; right column: quality-filtered test set. Top row: seen subset only (species-adduct pair observed during training). Bottom row: all spectra including novel-adduct and unseen species. Only adducts with $\geq$3 spectra are shown. Sample sizes are annotated above each bar group.*

S5.9 Analysis of limitations and practical guidance

While the results overall demonstrate the feasibility of transformer-based lipid identification from tandem mass spectra, several failure modes and boundary conditions should be considered when applying LipiDetective in practice. Many of these limitations stem from the composition of the current training set and can be addressed by expanding it, which is a priority for future versions of LipiDetective.

Instrument compatibility

LipiDetective was trained predominantly on beam-type CID spectra and performs substantially worse on ion trap instruments, which might fail to retain low-mass diagnostic fragments. Users working with ion trap data should interpret predictions with caution or consider retraining the model on instrument-specific reference data.

Adduct coverage

The model's performance is strongly dependent on the specific lipid class-adduct combinations encountered during training. While most common adducts are individually represented in the training data, the training set creates near-exclusive associations between certain adducts and lipid classes (e.g., [M+OAc]$^-$ almost exclusively with PC). When the model encounters a class-adduct combination absent from training, even if both the class and the adduct are individually well-known, performance degrades substantially. This limitation is particularly relevant for adducts that produce markedly different fragmentation patterns depending on the lipid class, as the model cannot generalize fragmentation rules across adduct types it has not observed for a given class.

Spectra with few informative fragments

Spectra containing very few informative fragment peaks, such as those acquired at extremely low collision energies or under poor signal-to-noise conditions, provide insufficient diagnostic information for reliable identification. In the MassBank evaluation, spectra with fewer than 10 peaks achieved 14.2% accuracy compared to 32.3% for spectra with 200+ peaks. Users should treat predictions from sparse or noisy spectra with increased caution, particularly when the confidence score is low.

Out-of-distribution lipid classes and species

For lipid species entirely absent from training, exact-match accuracy drops to 1-2%. However, the model's compositional output vocabulary enables partial identification even in this regime: average component accuracy for unseen species reaches $\sim$34%, indicating that the model can often correctly identify individual structural components (e.g., a known headgroup or fatty acid chain) even when the full species prediction is incorrect. For lipid classes not represented in the training vocabulary at all, the model may produce hallucinated predictions from rare training classes rather than indicating uncertainty. Users should be aware that predictions for lipid classes or species far outside the training distribution are unreliable.

Co-fragmented and chimeric spectra

As with most identification tools, LipiDetective assumes that each MS2 spectrum is dominated by a single lipid precursor. Co-fragmentation of multiple species or mixed spectra may lead to inconsistent or biologically implausible predictions, particularly when fragments from different lipids are present. Lipid classes or fatty acid motifs that are poorly represented or absent from the training dataset are more likely to be mispredicted or biased toward more common species. Component-wise accuracy analyses indicate that rare fatty acids and highly unsaturated chains are particularly affected by this limitation. To try to account for such ambiguity, LipiDetective can optionally return the top three candidate predictions together with their associated probability scores. In cases of co-fragmentation or uncertain spectra, the distribution of probabilities across multiple candidates can provide a useful indication of ambiguity, enabling users to apply additional filtering or manual validation steps.

Interpreting confidence scores

LipiDetective reports a beam search probability score for each prediction. On the internal validation set (beam-type CID instruments), these scores are reasonably well-calibrated. However, on external data or instrument types not well-represented in training, the model tends toward overconfidence: in the MassBank evaluation, predictions with 60-80% reported confidence corresponded to actual accuracy below 25% (ECE = 37.1% on the raw test set vs. 18.2% after quality filtering. High-confidence predictions ($>$80%) on compatible instruments are substantially more reliable, but users should not interpret the probability score as a calibrated posterior probability, particularly for instrument types or adducts underrepresented in training. When LipiDetective's prediction disagrees with conventional rule-based or spectral-library tools, users should consider the instrument type, adduct, and confidence score before deciding which identification to trust. Discrepancies are most likely to arise for instrument types where LipiDetective's training data is sparse, in which case the conventional tool's identification should generally be preferred.
